# Bond-length distributions for ions bonded to oxygen: metalloids and post-transition metals

**DOI:** 10.1107/S2052520617017437

**Published:** 2018-01-12

**Authors:** Olivier Charles Gagné, Frank Christopher Hawthorne

**Affiliations:** a Geological Sciences, University of Manitoba, 125 Dysart Road, Winnipeg, Manitoba R3T 2N2, Canada

**Keywords:** bond lengths, metalloids, post-transition metals, lone-pair electrons, lone-pair stereoactivity, oxides, oxysalts

## Abstract

Bond-length distributions have been examined for 33 configurations of the metalloid ions and 56 configurations of the post-transition metal ions bonded to oxygen, for 5279 coordination polyhedra and 21 761 bond distances for the metalloid ions, and 1821 coordination polyhedra and 10 723 bond distances for the post-transition metal ions. For the metalloid and post-transition elements with lone-pair electrons, the more common oxidation state between *n* *versus* *n*+2 is *n* for Sn, Te, Tl, Pb and Bi and *n*+2 for As and Sb. There is no correlation between bond-valence sum and coordination number for cations with stereoactive lone-pair electrons when including secondary bonds, and both intermediate states of lone-pair stereoactivity and inert lone pairs may occur for any coordination number > [4]. Variations in mean bond length are ∼0.06–0.09 Å for strongly bonded oxyanions of metalloid and post-transition metal ions, and ∼0.1–0.3 Å for ions showing lone-pair stereoactivity. Bond-length distortion is confirmed to be a leading cause of variation in mean bond lengths for ions with stereoactive lone-pair electrons. For strongly bonded cations (*i.e.* oxyanions), the causes of mean bond-length variation are unclear; the most plausible cause of mean bond-length variation for these ions is the effect of structure type, *i.e.* stress resulting from the inability of a structure to adopt its characteristic *a priori* bond lengths.

## Abstract 2

Bond-length distributions are examined for 33 configurations of the metalloid ions and 56 configurations of the post-transition metal ions bonded to oxygen. Lone-pair stereoactivity is discussed.

## 1. Introduction   

This paper is the third in a series [Gagné & Hawthorne (2016*a* ▸, 2018 ▸); see also Gagné (2018 ▸) in this issue] on the bond-length distributions of ions bonded to oxygen in crystals, and will focus on the metalloid and post-transition metal ions. For a detailed introduction and rationale for this work and a description of the data-collection and data-filtering methods, see Gagné & Hawthorne (2016*a* ▸). In this series, we examine the distribution of bond lengths for 135 ions bonded to oxygen in 462 configurations using 180 331 bond lengths extracted from 9367 refined crystal structures; these data involve most ions of the periodic table and all coordination numbers in which they occur. Working with a large amount of data allows examination of subtle differences between the shapes of various distributions (*e.g.* bond-length distributions, mean bond-length distributions) which reflect differences in their structural and/or electronic behaviour. The factors that affect bond lengths are of general interest to all who work on crystal structures and their properties, and a comprehensive analysis of all the data should lead to increased understanding of those factors. Moreover, knowledge of possible variation in bond lengths is important in evaluating computational results on structural arrangements by setting expectations and limits as to what bond lengths may be observed between ion pairs, and are also useful in identifying unusual stereochemical features in new crystal structures.

Here, we report the data and bond-length distributions for nine metalloid ions and 11 post-transition metal ions bonded to O^2−^: we report 33 configurations of the metalloid ions as a function of coordination number when bonded to O^2−^ (21 761 bond lengths and 5279 coordination polyhedra from 2575 crystal structure refinements), and 56 configurations for the post-transition metals (10 723 bond lengths and 1821 coordination polyhedra from 1143 crystal structure refinements). This article covers some strongly bonded oxyanions (*e.g.* BO_3_, SiO_4_) and ions with stereoactive lone-pair electrons (*e.g.* Sn^2+^, Tl^+^), and complements our discussion of these types of ions for non-metals bonded to O^2−^ (Gagné & Hawthorne, 2018 ▸).

## 2. Lone-pair stereoactivity   

Of the 135 ions for which we have gathered data in our bond-length dispersion analysis, we observe 14 cations with lone-pair electrons bonded to O^2−^, and 11 ions with stereoactive lone-pair electrons bonded to O^2−^. For the ions with stereoactive lone-pair electrons, seven ions are non-metals, three ions are metalloids and four ions are post-transition metals. For a thorough discussion of lone-pair stereoactivity and a general analysis for the 11 ions with stereoactive lone-pair electrons bonded to O^2−^, we refer the reader to the second paper of this series (Gagné & Hawthorne, 2018 ▸); here, we reiterate some important points, and give a more detailed discussion of lone-pair stereoactivity for the metalloids and post-transition metals later in text.

Lone-pair stereoactivity is associated with the *n* *s* ^2^ *n* *p* ^0^ electron configuration of *p*-block cations and the formation of highly anisotropic coordination polyhedra. Lone-pair stereoactive ions typically form short (strong) bonds in one hemisphere of their coordination shell, and long (weak) bonds in the other; these are commonly called ‘primary’ and ‘secondary’ bonds (Alcock, 1972 ▸). Lone-pair stereoactivity has successfully been explained *via* atomic orbital arguments (see below), but we note that it has also been rationalized using strictly Lewis acid–base arguments by Brown & Faggiani (1980 ▸) and Brown (1988 ▸, 2011 ▸) with some success.

Orgel (1959 ▸) first described the origins of the stereochemical behaviour for *ns* ^2^ *np* ^0^ cations based on the mixing of the non-bonding *s* and *p* orbitals of these cations in non-cubic environments. Orgel argued that the *sp*-hybridized orbitals, where the stereoactive lone-pair electrons reside, can only form at non-centrosymmetric sites due to the parity constraint of these orbitals, and that this can only be achieved *via* large distortions of the coordination polyhedra. Bersuker (1984 ▸) explained the occurrence of lone-pair stereoactivity *via* an energetically favourable interaction between the highest occupied molecular orbital (HOMO) of the cation and the lowest unoccupied molecular orbital (LUMO) of the anion; this was supported by many investigations in the years following (*e.g.* Lefebvre *et al.*, 1987 ▸, 1998 ▸; Watson & Parker, 1999 ▸; Watson *et al.*, 1999 ▸; Seshadri & Hill, 2001 ▸; Waghmare *et al.*, 2003 ▸; Stoltzfus *et al.*, 2007 ▸). From these findings, Walsh *et al.* (2011 ▸) gave a revised model of lone-pair stereoactivity with explicit dependence on the anion, where strong interactions between the cation *s* and anion *p* orbitals result in a high-energy antibonding state, which, *via* distortion of the crystal structure, may interact with the empty cation *p* orbitals to form an electronic state where the lone pair resides.

We note that although the VSEPR model (Gillespie & Nyholm, 1957 ▸; Gillespie, 1972 ▸) is commonly used to illustrate the bonding geometry of ions with stereoactive lone-pair electrons, it provides no driving mechanism for lone-pair stereoactivity/inactivity, and fails to explain the many cases for which lone-pair electrons are inactive, *i.e.* high-symmetry environments.

## 3. Coordination number   

Whereas coordination number may be defined in simple terms, *e.g.* the number of counterions bonded to an ion, the decision to consider atom pairs as ‘bonded’ is not obvious in many situations. This is particularly true for ions with stereoactive lone-pair electrons, as their coordination polyhedra are prone to large distortions, can form secondary bonds (up to ∼4 Å in length), and may be observed in a wide spectrum of ‘intermediate states’ between stereoactivity and inactivity of the lone-pair electrons (Galy *et al.*, 1975 ▸).

Gagné & Hawthorne (2015 ▸, 2016*a* ▸) provided arguments for including the longer interatomic distances of the first coordination shell for lone-pair stereoactive ions and for the larger alkali and alkaline earth metals as ‘bonded’, by analyzing (1) trends in the bond-valence parameters of these ions, and (2) the gap between the first and second coordination shell. This analysis is summarized in the previous paper of this series (Gagné & Hawthorne, 2018 ▸). As we did for the non-metal ions with lone-pair stereoactive electrons, here we derive coordination polyhedra using the method described by Gagné & Hawthorne (2016*a* ▸), which leads to the inclusion of all interatomic distances in the first coordination shell of the cations. This method leads to observed coordination numbers up to [12] for four lone-pair stereoactive cations, Tl^+^, Pb^2+^, Bi^3+^ and Te^4+^, and to coordination numbers up to [14] for Ba^2+^, [15] for K^+^, [18] for Rb^+^, and [20] for Cs^+^ (Gagné & Hawthorne, 2016*a* ▸).

The inclusion of the ‘longer interatomic distances’ follows the work of Alig & Trömel (1992 ▸) as well as that of Preiser *et al.* (1999 ▸) who provided theoretical evidence that some of the longer cation–anion distances (up to 4 Å) may contribute to weak but significant chemical bonding *via* the calculation of electrostatic fluxes.

## 4. Sample size   

Dealing with a very large amount of data has allowed us to critically evaluate the reproducibility of our results as a function of sampling. We described the effects of sample size (*e.g.* the presence of outliers, non-random sampling) in the first paper of this series (Gagné & Hawthorne, 2016*a* ▸), as well as the effect of sample size on grand mean bond length (and its standard deviation), skewness, and kurtosis for ^[6]^Na^+^ bonded to O^2−^. We reported the effect of sample size on these values for ^[4]^S^6+^ and ^[6]^I^5+^ bonded to O^2−^ in the second paper of this series (Gagné & Hawthorne, 2018 ▸). Here, we do a similar analysis for ^[4]^Si^4+^ and for ^[8]^Bi^3+^. This analysis is done to sample bond strengths not covered by Gagné & Hawthorne (2016*a* ▸, 2018 ▸), as Gagné & Hawthorne (2018 ▸) showed dependence of grand mean bond length, skewness and kurtosis values on bond strength and multi-modality of the bond-length distribution. Here we sample similar but weaker bonds for Si—O (mean bond valence 1 v.u.) compared to ^[4]^S^6+^—O^2−^ (mean bond valence 1.5 v.u.), and for ^[8]^Bi^3+^—O^2−^ (0.375 v.u.) compared with ^[6]^I^5+^—O^2−^ (0.83 v.u.) for lone-pair stereoactive cations. We report the sample sizes as a function of the number of coordination polyhedra.

**Figure 1:**
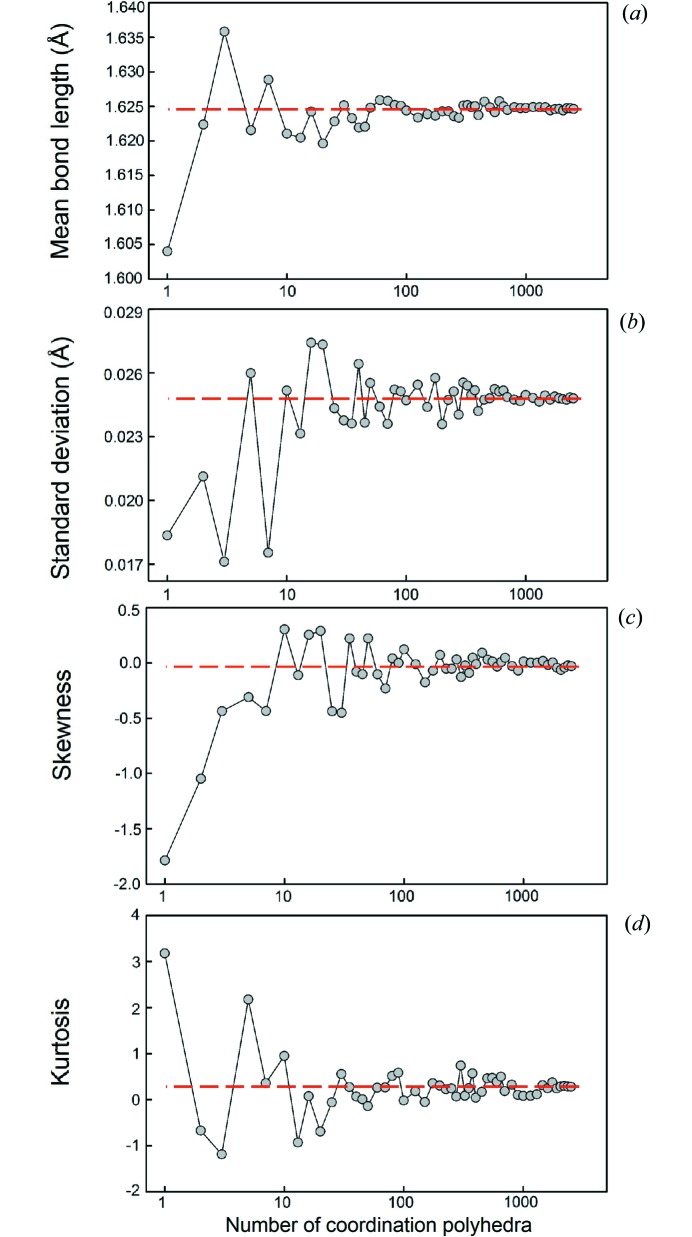
The effect of sample size on (*a*) mean bond length, (*b*) standard deviation of the mean bond length, (*c*) skewness, and (*d*) kurtosis for ^[4]^Si^4+^. The dashed line shows the value for the parent distribution.

**Figure 2:**
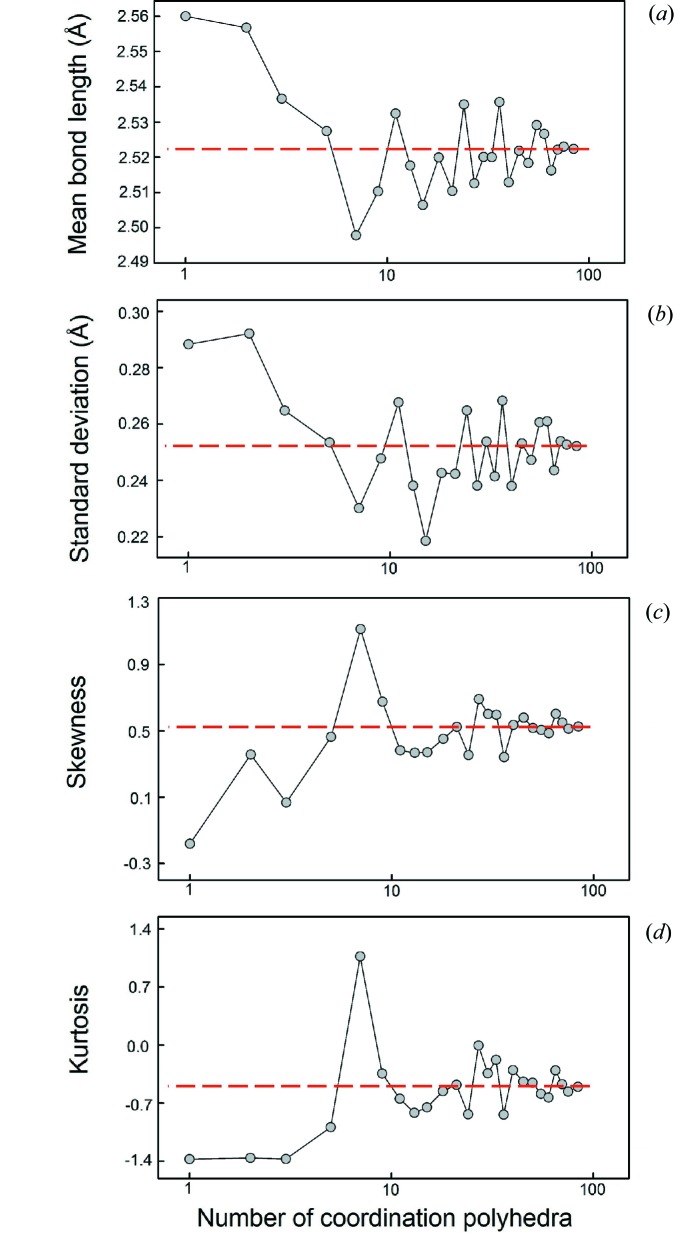
The effect of sample size on (*a*) mean bond length, (*b*) standard deviation of the mean bond length, (*c*) skewness, and (*d*) kurtosis for ^[8]^Bi^3+^. The dashed line shows the value for the parent distribution.

Fig. 1 ▸ shows that for ^[4]^Si^4+^, variation of less than ±0.005 Å in grand mean bond length is observed for sample sizes greater than 25 coordination polyhedra, while reliable values of skewness (±0.2) and kurtosis (±0.6) are obtained for sample sizes greater than 70 coordination polyhedra. For ^[8]^Bi^3+^ (Fig. 2 ▸), variability of less than ±0.005 Å is observed for 70 or more coordination polyhedra. However, it is possible that an appropriate sample size requires more than 70 coordination polyhedra but is limited here by the size of the parent distribution. Reliable values of skewness and kurtosis are obtained for sample sizes greater than seven coordination polyhedra.

Thus ^[8]^Bi^3+^ compares very well with ^[6]^I^5+^ (∼40 coordination polyhedra for the same level of agreement for grand mean bond lengths, and only two coordination polyhedra for skewness and kurtosis; Gagné & Hawthorne, 2018 ▸) despite significantly weaker bond strengths, due to the overwhelming effect of lone-pair stereoactivity on the bond-length distributions of these ions. For ^[4]^Si^4+^, more data is needed than for ^[4]^S^6+^ (approximately five coordination polyhedra; Gagné & Hawthorne, 2018 ▸) for a reliable value of the grand mean bond length, probably due to the formation of relatively weaker bonds. However, significantly less data are needed for ^[4]^Si^4+^ in comparison to ^[4]^S^6+^ (∼300 coordination polyhedra; Gagné & Hawthorne, 2018 ▸) to obtain reliable values of skewness and kurtosis.

Mean bond-length distributions were analyzed in a similar way. Minimum sample sizes were determined for the skewness and kurtosis of these distributions with the same cut-offs as above, less than which these values have little significance and are not reported. For ^[4]^Si^4+^, the threshold was observed at ∼400 coordination polyhedra (∼700 for ^[4]^S^6+^; Gagné & Hawthorne, 2018 ▸) and ∼60 coordination polyhedra for ^[8]^Bi^3+^, (∼50 for ^[6]^I^5+^; Gagné & Hawthorne, 2018 ▸).

## 5. Results   

### 5.1. Metalloids   

**Figure 3:**
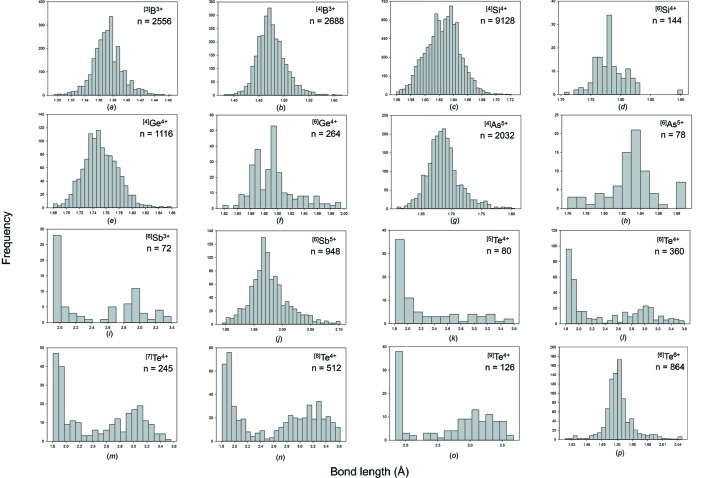
Bond-length distributions for selected configurations of the metalloid ions bonded to O^2−^: (*a*) ^[3]^B^3+^, (*b*) ^[4]^B^3+^, (*c*) ^[4]^Si^4+^, (*d*) ^[6]^Si^4+^, (*e*) ^[4]^Ge^4+^, (*f*) ^[6]^Ge^4+^, (*g*) ^[4]^As^5+^, (*h*) ^[6]^As^5+^, (*i*) ^[6]^Sb^3+^, (*j*) ^[6]^Sb^5+^, (*k*) ^[5]^Te^4+^, (*l*) ^[6]^Te^4+^, (*m*) ^[7]^Te^4+^, (*n*) ^[8]^Te^4+^, (*o*) ^[9]^Te^4+^, (*p*) ^[6]^Te^6+^.

For the metalloid ions bonded to O^2−^, the collection and filtering criteria described in Gagné & Hawthorne (2016*a* ▸) resulted in a sample of 21 761 bonds and 5279 coordination polyhedra. Table 1 ▸ gives the mean bond length and standard deviation, the minimum and maximum bond length (and range), the skewness and kurtosis (where justified by sample size), and the number of bonds and coordination polyhedra for the 33 configurations for which the nine metalloid ions are observed in. All bond-length and bond-valence distributions (using the bond-valence parameters of Gagné & Hawthorne, 2015 ▸) are shown in Figs. S1 and S2 (supporting information), respectively; bond-length distributions with adequate sample sizes (see above) are given in Fig. 3 ▸.

An important issue in proposing bond-length ranges that ions may adopt is the reliability of the data at the limits of its distribution, *i.e.* the shortest and longest bonds of each ion configuration; below we examine some of the data at the lower and upper limits of these distributions. Special attention was paid to identifying short and long bond lengths that were the result of disorder, substitution of other ions, anomalous displacement parameters and uncorrected twinning effects.

#### 5.1.1. B^3+^   

B^3+^ occurs in three coordination numbers [2], [3] and [4], with a slight preference for [3] relative to [4]. [2]-coordination occurs in only four structures (Calvo & Faggiani, 1974 ▸; Calvo *et al.*, 1975 ▸) where a BO_2_ group is aligned parallel to the *c* axis in synthetic apatite structures. The presence of B in these crystals was confirmed by chemical analysis and by ^11^B NMR. These structures did not quite pass our filters, but the occurrence of ^[2]^B^3+^ was thought to be sufficiently significant that it should be noted.

^[3]^B^3+^—O^2−^ distances are in the range 1.298–1.464 Å with a grand mean value of 1.372 Å; the latter is close to the value given for ^[3]^B^3+^—O^2−^ distances in minerals by Hawthorne *et al.* (1996 ▸): 1.370 Å. There is one very short ^[3]^B^3+^—O^2−^ distance of 1.298 Å in the structure of CsBO_2_ (Schläger & Hoppe, 1994 ▸). The constituent anion is coordinated by one ^[3]^B^3+^ and five Cs^+^ anions between 3.029 and 3.251 Å with an incident bond-valence sum of 1.864 v.u.; this sum is low, although not unusually so, and would need an even shorter ^[3]^B^3+^—O^2−^ distance to increase the sum. Hence this value seems a reliable minimum distance at present. Er_2_Cl_2_(B_2_O_5_) (Nikelski & Schleid, 2003 ▸) has a ^[3]^B^3+^—O^2−^ distance of 1.453 Å to a O^2−^ ion that bridges two (BO_3_) groups. The anion also bonds to Er^3+^ and the sum of the incident bond valences is 1.956 v.u. This is the longest reliable ^[3]^B^3+^—O^2−^ distance. The skewness of the distribution is very low, as expected for an ion with high bond valences and small coordination number.

^[4]^B^3+^—O^2−^ distances are in the range 1.380–1.616 Å with a grand mean value of 1.475 Å; the value given for the grand mean ^[4]^B^3+^—O^2−^ distance in minerals by Hawthorne *et al.* (1996 ▸) is 1.476 Å. The structure of Gd_2_(B_4_O_9_) (Emme & Huppertz, 2003 ▸) has edge-sharing (BO_4_) groups and both very short (1.380 Å) and very long (1.603 Å) ^[4]^B^3+^—O^2−^ distances. The sum of the incident bond valences at the central B^3+^ ion is 2.850 v.u and at the anions is 1.796 and 2.206 v.u. The structure is well refined and these distances seem reliable. Longer ^[4]^B^3+^—O^2−^ distances have been published: the structure of piergorite-(Ce) (Boiocchi *et al.*, 2006 ▸) lists a ^[4]^B^3+^—O^2−^ distance of 1.664 (6) Å. However, the 〈^[3]^B^3+^—O^2−^〉 distance is 1.525 Å, much too large for occupancy of the tetrahedrally coordinated site by B^3+^ alone. In accord with this, the *U*_eq_ value for the central *B* site is 2.4× the mean value of the other three *B* sites in the structure, indicating that there is substitution for B^3+^ by a heavier cation, almost certainly Si^4+^ that leads to the anomalously large 〈^[3]^B^3+^—O^2−^〉 distance. Thus the data for piergorite-(Ce) was not included in our analysis.

#### 5.1.2. Si^4+^   

Si^4+^ occurs in two coordination numbers: [4] and [6], with a very strong preference for [4] over [6]: 2282 *versus* 24 polyhedra, respectively (Table 1 ▸). [6]-coordination is generally associated with high-pressure phases, although thaumasite, Ca_3_Si(OH)_6_(CO_3_)(SO_4_)(H_2_O)_12_ (Jacobsen *et al.*, 2003 ▸) contains [6]-coordinate Si^4+^ and occurs as a low-temperature secondary alteration phase in mafic igneous and metamorphic rocks. ^[4]^Si^4+^—O distances are in the range 1.560–1.726 Å with a grand mean value of 1.625 Å, close to the value of 1.624 Å given by Baur (1978 ▸). Si^4+^—O distances smaller than 1.56 Å are commonly recorded, but are associated with high variability in *U*_eq_ values, substitution of B^3+^ and P^5+^ for Si^4+^, and/or disorder of other cations in the structure. Our estimate of a reliable minimum Si^4+^—O^2−^ distance is 1.560 Å. In the type-B (high-pressure) *R*_2_Si_2_O_7_ (*R* = Gd, Tb, Dy, Ho) structures, the longest Si^4+^—O^2−^ distances to bridging anions are in the range 1.708–1.725 Å in well refined structures (Fleet & Liu, 2003 ▸). In these four structures, the bond-valence sums at the anions involved in the longest Si^4+^—O^2−^ distances are 2.09, 2.12, 2.09 and 2.12 v.u. for *R* ^3+^ = Gd, Tb, Dy, Ho, respectively. Thus the longest reliable Si^4+^—O^2−^ distance is 1.726 Å. The bond-length distribution for Si^4+^O_4_ has unusually low values of skewness (0.0) and kurtosis (0.0).

^[6]^Si^4+^—O^2−^ distances are in the range 1.706–1.903 Å with a grand mean value of 1.783 Å. There is one very short ^[6]^Si^4+^—O^2−^ distance of 1.706 Å in the structure of SiPO_4_(OH) (Stearns *et al.*, 2005 ▸). However, the sum of the bond valences at the constituent anion is 2.077 v.u., suggesting that this is a valid distance. Pacalo & Parise (1992 ▸) report a ^[6]^Si^4+^—O^2−^ distance of 1.903 Å, significantly larger that the next-lowest values around 1.83 Å. There is no apparent flaw in the structure refinement, but the sum of the bond valences incident at the constituent anion is 1.859 v.u. The ^[6]^Si^4+^—O^2−^ distance required for exact adherence to the valence-sum rule is 1.804 Å, within the range of values observed in other structures. However, the small number of data leave the possible maximum length of the ^[6]^Si^4+^—O^2−^ bond an open question.

#### 5.1.3. Ge^4+^   

Ge^4+^ occurs in three coordination numbers: [4], [5] and [6], with a very strong preference for [4] and a slight preference for [6] over [5]. ^[4]^Ge^4+^—O^2−^ distances are in the range 1.680–1.859 Å with a grand mean value of 1.752 Å. The largest value of 1.859 Å occurs in the structure of Ca_5_Ge_3_O_11_ (Barbier & Levy, 1997 ▸). The structure is well refined and the O^2−^ ion bridges two (GeO_4_) tetrahedra and bonds to two additional Ca^2+^ ions for an incident bond-valence sum of 2.176 v.u. The ^[4]^Ge^4+^—O distance of 1.844 Å occurs in the structure of Fe_2_Ge_2_O_8_ (Kato *et al.*, 1979 ▸); the constituent anion bridges two (GeO_4_) tetrahedra and bonds to an additional Fe^2+^ ion for an incident bond-valence sum of 2.046 v.u. Thus the tail to higher values in Fig. 3 ▸(*e*) is a result of a small number of linked (GeO_4_) tetrahedra in structures where the bridging anion bonds to other cations. Similar to ^[4]^Si^4+^, the distribution of ^[4]^Ge^4+^—O^2−^ distances shows low skewness (0.4) and kurtosis (0.6) (Table 1 ▸). ^[5]^Ge^4+^—O distances are in the range 1.719–2.117 Å with a grand mean value of 1.847 Å, although the number of data is small. ^[6]^Ge^4+^—O^2−^ distances are in the range 1.818–1.995 Å with a grand mean value of 1.894 Å. The distribution of ^[6]^Ge^4+^—O^2−^ distances shows a tail to longer values, and examination of these structures shows that these distances involve O^2−^ ions that bridge (GeO_6_) octahedra and link to other divalent cations.

#### 5.1.4. As^3+^   

As^3+^ occurs in five coordination numbers from [3] to [8] with an average observed coordination number of [5] and a grand mean bond length of 2.107 Å for 28 polyhedra. As^3+^ is strongly lone-pair stereoactive and despite the paucity of data, all coordination numbers above [3] show bimodal distributions of bond lengths. There are always three short primary bonds for all coordination numbers in the range 1.671–1.891 Å with a mean value of 1.793 Å, to be compared with a grand mean value of 1.783 Å for minerals given by Majzlan *et al.* (2014 ▸). There is a gap of >0.80 Å between the primary bonds and the shortest secondary bonds for all coordination numbers > [3].

#### 5.1.5. As^5+^   

As^5+^ occurs in two coordination numbers: [4] and [6] with [4] dominant over [6] (Table 1 ▸). For [4]-coordination, the grand mean bond length is 1.687 Å, close to the value of 1.685 Å given by Majzlan *et al.* (2014 ▸) for minerals; the individual bond-length range is 1.610–1.806 Å. The distribution shows a long tail to larger values (Fig. 3 ▸ *g*), but these data are from well refined structures and are reasonable from a crystal-chemical point of view. The structure of CaK_2_As_2_O_7_ (Faggiani & Calvo, 1976 ▸) has a diarsenate group; the bridging O^2−^ ion has ^[4]^As^5+^—O^2−^ distances of 1.799 and 1.786 Å with an additional Ca^2+^—O^2−^ bond of 2.875 Å for an incident bond-valence sum of 1.976 v.u. Distances of 1.795 and 1.790 Å to two different O^2−^ ions are listed in the structure of TlH_2_AsO_4_ (Narasaiah *et al.*, 1987 ▸). The constituent anions also bond to Tl^+^ at 2.949 and 2.965 Å for bond-valence sums of 1.043 and 1.049 v.u., respectively. In accord with the composition of the crystal, the valence-sum rule indicates that these anions are OH groups and each receives a bond-valence contribution from the associated H^+^ ion, bringing the incident bond-valence close to 2 v.u. Thus the tail of long values for ^[4]^As^5+^—O^2−^ bonds (Fig. 3 ▸ *g*) is due to a small number of polymerized and acid (AsO_4_) groups.

For [6]-coordination, the grand mean bond length is 1.830 Å with an individual bond-length range of 1.767–1.888 Å; this is the smallest range for any [6]-coordinated metalloid cation, although this may be a result of the small amount of data available (Table 1 ▸).

#### 5.1.6. Sb^3+^   

Sb^3+^ occurs in seven coordination numbers from [3] to [9] with an average observed coordination number of [6] and a grand mean bond length of 2.278 Å for 52 polyhedra. Sb^3+^ is strongly lone-pair stereoactive. For ^[3]^Sb^3+^, there are no secondary bonds and the grand mean bond length is correspondingly short: 1.932 with a range of 1.899–1.982 Å. The grand mean bond lengths increase monotonically with increasing coordination number as the number of secondary bonds increases. The number of primary bonds varies from three (most common) to two examples of five in NaSb_3_O_2_(PO_4_)_2_ (Adair *et al.*, 2000 ▸): 2.310, 1.982, 2.301, 2.121, 2.149 Å and 2.038, 2.301, 2.147, 2.113, 2.296 Å, and the division between primary and secondary bonds is less pronounced than in other lone-pair stereoactive ions.

#### 5.1.7. Sb^5+^   

Sb^5+^ occurs in coordination number [6] with a grand mean bond length of 1.978 Å and a range of 1.894–2.102 Å for 183 polyhedra. The lower limit of 1.894 Å is indicated by several well refined structures with minimum Sb^5+^—O^2−^ distances in the range 1.89–1.90 Å. The longest reliable Sb^5+^—O^2−^ distance is in Sb_2_O_5_ (Jansen, 1978 ▸) where an O^2−^ ion is bonded to three Sb^5+^ ions at distances of 2.043, 2.085 and 2.102 Å for an incident bond-valence sum of 2.041 v.u., thus giving a crystal-chemical justification for the long observed Sb^5+^—O^2−^ distance.

#### 5.1.8. Te^4+^   

Te^4+^ occurs in ten coordination numbers from [3] to [12] with most data observed in coordination numbers [6] and [8]; the grand mean bond length is 2.469 Å for 211 polyhedra. Te^4+^ is strongly lone-pair stereoactive and most of the coordination numbers show a bimodal distribution of bond lengths [Figs. 3(*k*)–3(*o*), and Figs. S1(*x*)–1(*ag*)]. For ^[3]^Te^4+^, there are no secondary bonds and the grand mean bond length is correspondingly short: 1.843 with a range of 1.819–1.862 Å. The grand mean bond lengths increase monotonically with increasing coordination number as the number of secondary bonds increases. The number of primary bonds varies from three (most common) to five in NiTe_2_O_5_ (Platte & Trömel, 1981 ▸): 1.886, 1.996 ×2, 2.247 ×2 Å, and Te_3_SeO_8_ (Pico *et al.*, 1986 ▸): 1.886, 2.021, 2.032, 2.218 ×2 Å. As observed for Sb^3+^, this behaviour is somewhat different to that of Se^4+^ which shows only three primary bonds irrespective of its coordination number (Gagné & Hawthorne, 2018 ▸). The ranges of bond lengths found are broadly compatible with those of Christy *et al.* (2016 ▸).

#### 5.1.9. Te^6+^   

Te^6+^ occurs only in coordination number [6] with a grand mean bond length of 1.923 Å and a range of 1.817–2.048 Å for 155 polyhedra, compatible with the results of Christy *et al.* (2016 ▸). Much of the data is concentrated in the centre of the range and there are long tails to each side of the distribution. In other examples of such distributions, it has been our experience that much of the data in such long tails to the distribution are the result of extensive (atomic or stacking) disorder or unresolved twinning in the structure, inadequate absorption corrections for heavily absorbing structures. However, for Te^6+^ the situation is somewhat different. Such problem structures still occur, but other structures in the tails of the distribution look well refined and the resulting stereochemistry appears reasonable, at least from a bond-valence perspective. The structure of Na_2_Te_2_O_7_ (Meier & Schleid, 2006 ▸) has a short ^[6]^Te^6+^—O^2−^ distance of 1.817 Å and is bonded to three Na^+^ ions for an incident bond-valence sum of 1.874 v.u.

### 5.2. Post-transition metals   

**Figure 4:**
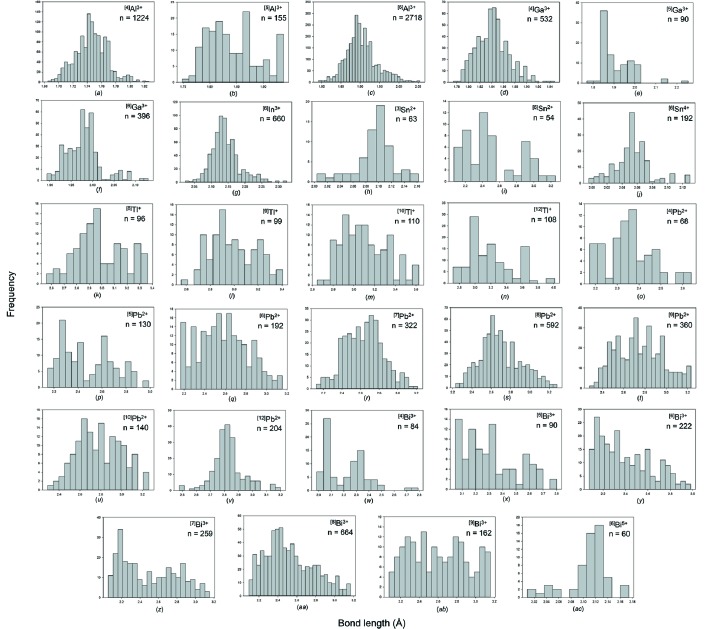
Bond-length distributions for selected configurations of the post-transition metal ions bonded to O^2−^: (*a*) ^[4]^Al^3+^, (*b*) ^[5]^Al^3+^, (*c*) ^[6]^Al^3+^, (*d*) ^[4]^Ga^3+^, (*e*) ^[5]^Ga^3+^, (*f*) ^[6]^Ga^3+^, (*g*) ^[6]^In^3+^, (*h*) ^[3]^Sn^2+^, (*i*) ^[6]^Sn^2+^, (*j*) ^[6]^Sn^4+^, (*k*) ^[8]^Tl^+^, (*l*) ^[9]^Tl^+^, (*m*) ^[10]^Tl^+^, (*n*) ^[12]^Tl^+^, (*o*) ^[4]^Pb^2+^, (*p*) ^[5]^Pb^2+^, (*q*) ^[6]^Pb^2+^, (*r*) ^[7]^Pb^2+^, (*s*) ^[8]^Pb^2+^, (*t*) ^[9]^Pb^2+^, (*u*) ^[10]^Pb^2+^, (*v*) ^[12]^Pb^2+^, (*w*) ^[4]^Bi^3+^, (*x*) ^[5]^Bi^3+^, (*y*) ^[6]^Bi^3+^, (*z*) ^[7]^Bi^3+^, (*aa*) ^[8]^Bi^3+^, (*ab*) ^[9]^Bi^3+^, (*ac*) ^[6]^Bi^5+^.

For the post-transition metals ions bonded to O^2−^, the collection and filtering criteria described in Gagné & Hawthorne (2016*a* ▸) resulted in a sample size of 10 723 bonds and 1821 coordination polyhedra. Table 2 ▸ gives the bond-length statistics for the 56 configurations for which the 11 post-transition metal ions are observed in. All bond-length and bond-valence distributions are shown in Figs. S3 and S4, respectively; bond-length distributions with adequate sample sizes are shown in Fig. 4 ▸.

#### 5.2.1. Al^3+^   

Al^3+^ has three coordination numbers: [4], [5] and [6]; [6] is dominant (*n* = 453) and then [4] (*n* = 306) with [5] being less common (*n* = 31). ^[4]^Al^3+^ has a grand mean bond length of 1.746 Å and a range of 1.685–1.833 Å. Distances below 1.68 Å occur in several structures but they all have potential for cation disorder and we consider them unreliable. ^[5]^Al^3+^ has a grand mean bond length of 1.842 Å and a range of 1.748–1.938 Å. ^[6]^Al^3+^ has grand mean bond length of 1.903 Å and a range of 1.792–2.054 Å. Values longer than 2.054 Å are given in the literature but these are associated with replacement of Al^3+^ by other ions. The longest confirmed bond distance of 2.054 Å occurs in the structure of BeAl_6_O_10_ (Alimpiev *et al.*, 2002 ▸) in which the constituent O^2−^ ion is coordinated by four Al^3+^ ions at distances of 2.054, 1.998, 1.880 and 1.861 Å for an incident bond-valence sum of 1.99 v.u.

#### 5.2.2. Ga^3+^   

Ga^3+^ has three coordination numbers: [4], [5] and [6]; [4] is dominant (*n* = 133) over [6] (*n* = 66) and [5] (*n* = 18). ^[4]^Ga^3+^ has a grand mean bond length of 1.842 Å and a range of 1.774–1.948 Å. The minimum reliable distance occurs in BaGa_2_O_4_ (Kahlenberg *et al.*, 2000 ▸) in which there is one O^2−^ ion bridging two GaO_4_ tetrahedra and not bonded to Ba^2+^. The ^[4]^Ga^3+^—O^2−^ distances are 1.774 and 1.801 Å; the sum of the incident bond valences is low at 1.724 v.u. but this may be due to structural strain as BaGa_2_O_4_ was synthesized at high temperature (1350^o^C) and a [2]-coordinated bridging anion has little possibility of relaxation with decreasing temperature except shortening of its ^[4]^Ga^3+^—O^2−^ bonds. The longest ^[4]^Ga^3+^—O^2−^ bonds are in the range 1.91–1.94 Å and occur in the structures of Sr_4_(Ga_2_O_7_) (Kahlenberg *et al.*, 2005 ▸) and Ba_4_(Ga_2_O_7_) (Kahlenberg, 2001 ▸). ^[5]^Ga has grand mean bond length of 1.910 Å and a range of 1.771–2.254 Å. The value of 2.254 Å is a very prominent outlier in the distribution of bond lengths [Fig. S3(*e*)]. It occurs in the structure of NaGa_2_(OH)(PO_4_)_2_ (Guesdon *et al.*, 2003 ▸); the constituent O^2−^ ion bonds to three Ga^3+^ ions and a H^+^ ion, and the incident bond-valence omitting the H^+^ ion is 1.06 v.u., suggesting that this long distance is valid. ^[6]^Ga^3+^ has grand mean bond length of 1.978 Å and a range of 1.893–2.130 Å. The distribution shows a long tail to longer values.

#### 5.2.3. In^3+^   

In^3+^ has three coordination numbers: [6], [7] and [8]. Coordination number [6] is most frequently observed and has a grand mean bond length of 2.142 Å and a range of 2.023–2.324 Å. Many of the shortest and longest reliable In^3+^—O^2−^ distances occur in the well ordered structure of In_4_(P_2_O_7_)_3_ (Thauern & Glaum, 2003 ▸). Using the coordination numbers of the cations, there are 4 × 6 + 6 × 4 = 48 bonds in the structure, and hence the mean coordination number of the anions is 48/21 = [2.29]; 15 anions have a coordination number of [2] and six anions have a coordination number of [3]. The [2]-coordinated anions bond to In^3+^ and P^5+^ for a Pauling bond-strength sum of 1.75 v.u. and hence the bonds to these cations must be shorter than usual. Thus the In^3+^—O^2−^ distances are very short, with three bonds in the range 2.023–2.036 Å, and the corresponding anion bond-valence sums are in the range 2.051–2.064 v.u. The [3]-coordinated anions bond to In^3+^ ×2 and P^5+^ for a Pauling bond-strength sum of 2.25 v.u. and hence the bonds to these cations must be longer than usual. As the structure contains P_2_O_7_ groups, the terminal P^5+^—O^2−^ bonds cannot lengthen significantly and thus the reduction in incident bond-valence must be accommodated by elongation of the In^3+^—O^2−^ bonds. Accordingly, there are six In^3+^—O^2−^ bonds in the range 2.24–2.30 Å, accounting for many of the long In^3+^—O^2−^ distances in Fig. 4 ▸(*g*), and the incident bond-valence sums are in the range 2.05–2.09 v.u. The longest reliable ^[6]^In^3+^—O^2−^ distance (2.324 Å) occurs in the structure of CuInW_2_O_8_ (Müller-Buschbaum & Szillat, 1994 ▸). The constituent anion also bonds to Cu^2+^ and W^5+/6+^ with an incident bond valence of 1.974 v.u.

#### 5.2.4. Sn^2+^   

Sn^2+^ occurs in seven coordination numbers from [3] to [9] with a grand mean bond length of 2.336 Å for 50 polyhedra. Sn^2+^ is strongly lone-pair stereoactive. For ^[3]^Sn^2+^, there are no secondary bonds and the grand mean bond length is 2.094 Å with a range of 2.004–2.162 Å. For ^[4]^Sn^2+^, the fourth bond distance varies from 2.470 to 2.881 Å. In most cases, all four distances should be regarded as primary bonds as they lie to one side (*i.e.* in one hemisphere of space to one side) of the cation. Thus in Sn_2_(S_2_O_4_)_2_ (Magnusson & Johansson, 1982 ▸), the four distances 2.236, 2.242, 2.264 and 2.324 Å lie to one side of the Sn^2+^ ion, whereas in Sn_3_O(OH)PO_4_ (Jordan *et al.*, 1980 ▸), one Sn^2+^ has four primary bonds at 2.065, 2.167, 2.281 and 2.470 Å whereas another Sn^2+^ has three primary bonds at 2.111, 2.138 and 2.167 Å and one secondary bond at 2.674 Å. Thus both arrangements, three primary bonds and four primary bonds, occur for ^[4]^Sn^2+^. The grand mean bond length increases monotonically with increasing coordination number as the number of secondary bonds increases (Table 2 ▸).

#### 5.2.5. Sn^4+^   

Sn^4+^ occurs in three coordination numbers, [4], [6] and [7]. Coordination number [6] is observed most frequently, and has a grand mean bond length of 2.054 Å with a range of 1.996–2.130 Å. The range of distances for ^[4]^Sn^4+^ is very small, 1.935–1.970 Å, with a mean value of 1.956 Å; this may be the result of the small number of data and the restricted range of compositions (alkali metal stannates).

#### 5.2.6. Tl^+^   

Tl^+^ occurs in ten coordination numbers from [3] to [12], all with a small number of data. ^[3]^Tl^+^ is strongly lone-pair stereoactive with three short bonds to one side of the Tl^+^ ion, as in Tl_6_Si_2_O_7_ (Piffard *et al.*, 1975 ▸). ^[4]^Tl^+^ also occurs in the structure of Tl_6_Si_2_O_7_ and is also lone-pair stereoactive with all four anions occurring on one side of the ^[4]^Tl^+^ ion at distances of 2.378, 2.721, 2.812 and 3.014 Å. There is a gradual increase in mean Tl^+^—O^2−^ distances with increasing coordination number. The difference between the primary and secondary bond lengths is not as great for Tl^+^ as for other lone-pair stereoactive ions, a result of the lower formal charge of Tl^+^ relative to other ions such as As^3+^ or Te^4+^.

#### 5.2.7. Tl^3+^   

Tl^3+^ occurs in three coordination numbers: [6], [7] and [8], and there is very little data (Table 2 ▸). The grand mean bond lengths increase from 2.228 to 2.336 to 2.378 Å with increasing coordination number.

#### 5.2.8. Pb^2+^   

Pb^2+^ occurs in ten coordination numbers from [3] to [12] with a preference for [8]. The grand mean bond length is 2.680 Å for 275 polyhedra. For ^[3]^Pb^2+^, there are no secondary bonds and the grand mean bond length is 2.210 Å with a range of 2.149–2.291 Å and all bonds lying to one side of the Pb^2+^ ion. For ^[4]^Pb^2+^, the fourth (long) distance varies from 2.367 to 2.638 Å, and in most cases, all four O^2−^ anions lie to one side of the cation. With increasing coordination number, there is no obvious development of bimodal distributions of bond lengths [Figs. 4(*o*)–4(*v*)] with the possible exception of [5] (Fig. 4 ▸ *p*). For [5]-coordination, all bonds can still be to one side of the cation, as in PbAl_2_O_4_ (Ploetz & Müller-Buschbaum, 1982 ▸). With increasing coordination number, this asymmetric distribution of coordinating anions can be lost, as in Pb(WO_4_) (Richter *et al.*, 1976 ▸) in which the eight bonds seem distributed reasonably randomly around the central cation.

#### 5.2.9. Pb^4+^   

Pb^4+^ occurs in coordinations [4], [5] and [6] with a preference for [6]. The grand mean bond lengths increase with increasing coordination number, but the paucity of data (Table 2 ▸) prevents any general conclusions.

#### 5.2.10. Bi^3+^   

Bi^3+^ occurs in nine coordination numbers from [3] to [12] with a marked preference for coordination [8] (Table 2 ▸). The grand mean bond length is 2.481 Å for 231 polyhedra. Bi^3+^ is strongly lone-pair stereoactive, but there is little sign of bimodal distributions of bond lengths [Figs. 4(*w*)–4(*ab*)], except perhaps for a coordination of [7] (Fig. 4 ▸ *z*). For ^[3]^Bi^3+^, there are no secondary bonds and the grand mean bond length is 2.069 Å with a range of 2.002–2.151 Å. For ^[4]^Bi^3+^, the fourth bond distance varies from 2.291–2.790 Å but is always a primary bond in that it lies within the hemisphere containing the primary (short) bonds. For ^[5]^Bi^3+^, the fifth bond distance varies from 2.336 to 2.785 Å but again is always a primary bond. For ^[6]^Bi^3+^, the fifth bond distance varies from 2.409 to 2.981 Å. The bonds do not now occupy a single hemisphere, but one or two just project into the second hemisphere; this is the case both for Bi(PO_3_)_3_ (Palkina & Jost, 1975 ▸) with a fifth bond of 2.435 Å and uranosphaerite, Bi(UO_2_)O_2_OH (Hughes *et al.*, 2003 ▸) with a fifth bond of 2.981 Å. At higher coordination numbers, the bonds are distributed more uniformly around the central cation, but the shortest three bonds still tend to be concentrated to one side of the central cation.

#### 5.2.11. Bi^5+^   

Bi^5+^ occurs in coordinations [4] and [6] with a preference for [6]. The distribution of distances for [6]-coordination shows a strong negative skewness, but this is probably the result of insufficient data (ten coordination polyhedra), as most other ions with strong negative skewness are characterized by very few data. The grand mean bond length for ^[6]^Bi^5+^ is 2.110 Å with a range of 2.009–2.174 Å.

## 6. Discussion   

### 6.1. Lone-pair stereoactivity for metalloids and post-transition metals   

In our bond-length dispersion analysis, three metalloid and four post-transition metal cations bonded to O^2−^ display lone-pair stereoactivity. These ions also occur in an *n*+2 oxidation state, *i.e.* with no lone-pair electrons. For the metalloids, the number of coordination polyhedra for the *n* and *n*+2 oxidation states are 28 *versus* 526 for As^3+^ and As^5+^, 54 *versus* 183 for Sb^3+^ and Sb^5+^, and 212 *versus* 155 for Te^4+^ and Te^6+^. For the post-transition metals, these numbers are 50 *versus* 38 for Sn^2+^ and Sn^4+^, 74 *versus* nine for Tl^+^ and Tl^3+^, 276 *versus* 12 for Pb^2+^ and Pb^4+^, and 231 *versus* 11 for Bi^3+^ and Bi^5+^. Therefore, two of the seven ions are observed more often in their *n*+2 oxidation state: the group 15 ions of periods 4 and 5, As^3+^ and Sb^3+^. Although this is also the case for P (the group 15 cation of period 3), this trend does not extend to Bi in period 6. In contrast, Gagné & Hawthorne (2018 ▸) showed that the period 3 non-metal ions more frequently occur in their highest oxidation state (without lone pair) when bonded to O^2−^ (P^5+^, S^6+^ and Cl^7+^), and in their lowest oxidation state (with lone pair) for the period 4 and 5 non-metals bonded to O^2−^ (Se^4+^, Br^5+^, I^5+^).

**Figure 5:**
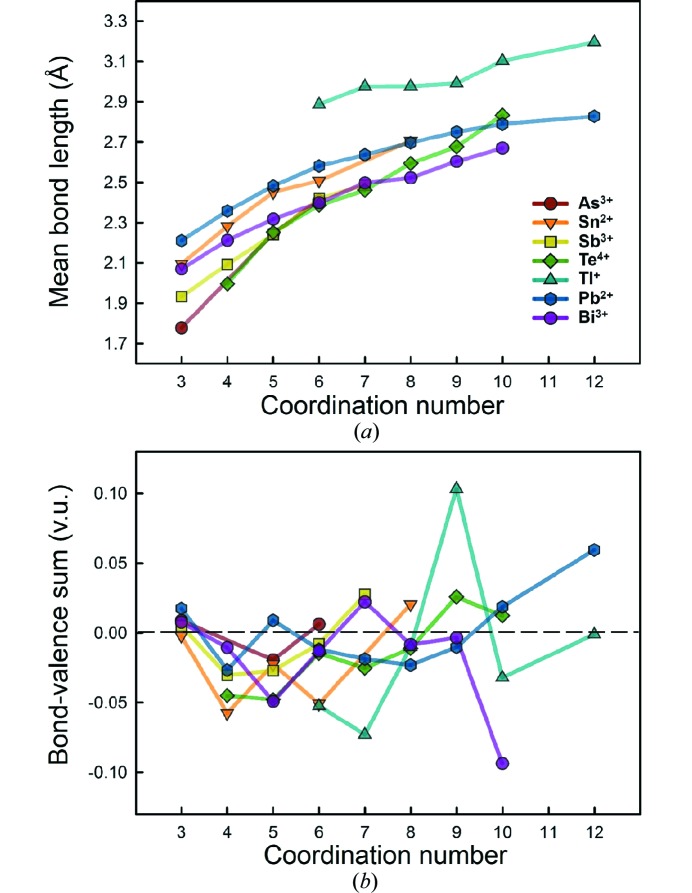
Values of (*a*) mean bond length (Å) and (*b*) mean bond-valence sum using the parameters of Gagné & Hawthorne (2015 ▸) for the different coordination numbers of the metalloid and post-transition metal ions.

When they are bonded to O^2−^, the metalloid and post-transition metal ions with stereoactive lone-pair electrons show no trend for the bond-length range and the skewness and kurtosis of the bond-length distribution; this is probably due to small sample size. In Fig. 5 ▸(*a*), we give mean bond length as a function of coordination number for the seven ions for sample sizes greater than five coordination polyhedra. Individual data points are clearly prone to error due to small sample size, but we nonetheless observe a somewhat regular increase with coordination number for these ions. The mean bond length may appear to increase in a logarithmic way for certain ions, but this is again probably due to small sample size for higher coordination numbers. For the alkali and alkaline earth metals bonded to O^2−^, Gagné & Hawthorne (2016*a* ▸) reported a linear increase for larger sample sizes. In Fig. 5 ▸(*b*), we give deviations from the bond-valence sum for the cations as a function of coordination number for the same ions. Contrary to what was observed for alkali and alkaline earth metal ions bonded to O^2−^ (Gagné & Hawthorne, 2016*a* ▸), here we see no correlation between bond-valence sum and coordination number. The bond-valence parameters used are those of Gagné & Hawthorne (2015 ▸), which were derived with a coordination-based optimization factor to minimize deviations as a function of coordination number; these deviations are otherwise large for other published sets of bond-valence parameters.

#### 6.1.1. When do we observe lone-pair stereoactivity?   

**Figure 6:**
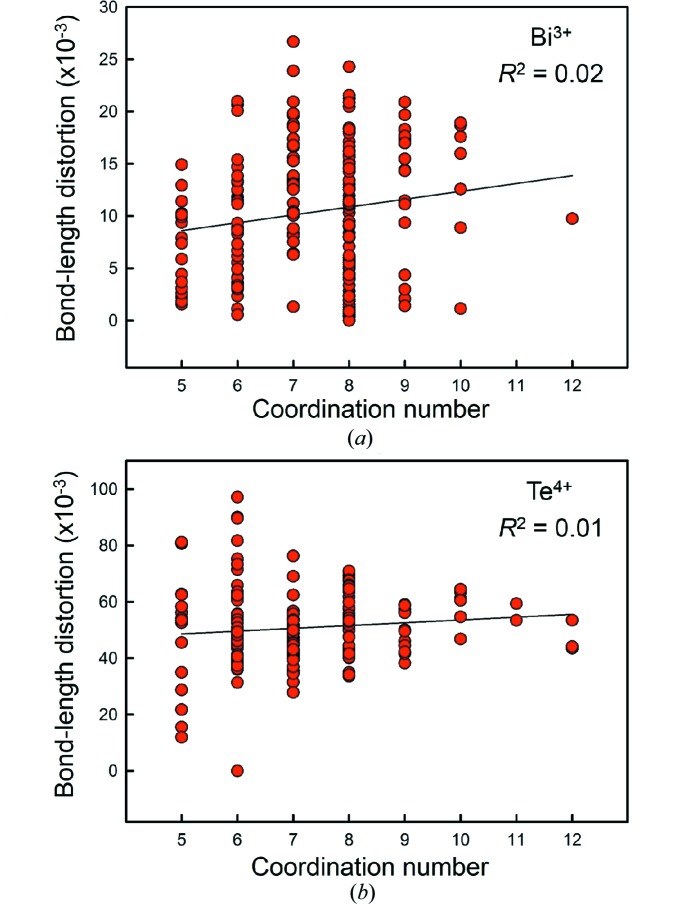
Bond-length distortion as a function of coordination number for (*a*) Bi^3+^ and (*b*) Te^4+^. The *p*-values are (*a*) 0.030 and (*b*) 0.139.

In a general examination of lone-pair stereoactivity for 14 non-metal, metalloid and post-transition metal cations with lone-pair electrons bonded to O^2−^, Gagné & Hawthorne (2018 ▸) confirmed the observation of Galy *et al.* (1975 ▸) that in the majority of cases, the lone-pair of cations is observed in an ‘intermediate state’ between stereoactivity and inertness. They also showed that interatomic distances may be included as secondary bonds in 1126 of 1321 coordination polyhedra surveyed (∼85%). Where the lone pair is ‘fully stereoactive’, the next-nearest anions are usually observed at 2–3× the distance of the mean bond length for the short bonds, too far and weak to be considered secondary bonds. Gagné & Hawthorne (2018 ▸) also showed that lone-pair stereoactivity (as measured by bond-length distortion) correlates very poorly to coordination number for Se^4+^ and Pb^2+^ (*R* ^2^ = 0.19 and 0.08, respectively), concluding that both intermediate and inert lone-pair electrons may occur for coordination numbers > [4]. In Fig. 6 ▸, we give a similar plot for (*a*) Bi^3+^ (*p*-value = 0.030, *R* ^2^ = 0.02) and (*b*) Te^4+^ (*p*-value = 0.139, *R* ^2^ = 0.01), confirming that there is no relation between lone-pair stereoactivity and coordination number for coordination numbers > [4].

This result follows the current model for lone-pair stereoactivity, which does not concern itself with coordination number. In this model (Walsh *et al.*, 2011 ▸), stereoactivity of the lone-pair electrons results from strong interactions between the cation *s* and anion *p* orbitals that result in a high-energy antibonding state. This antibonding state may then interact with the empty *p* orbitals of the cation *via* distortion of the crystal structure to form an electronic state where the lone pair resides; what coordination number will result from this is irrelevant to this phenomenon, and depends on the rest of the structure. Furthermore, whether or not distortion will result in a net stabilization of the occupied electronic states depends on the relative energy of the cation *s* and *p* and anion *p* orbitals, the prediction of which requires orbital energy calculations on a case-by-case basis.

Alternatively, Brown & Faggiani (1980 ▸) showed that simple Lewis acid–base arguments may be used to predict lone-pair stereoactivity. They gave a loose inverse relation between the coordination number of Tl^+^ and the base strength of the anion, proposing that lone-pair electrons are always stereoactive where the counterion is a strong base with Lewis basicity > 0.22 v.u. However, a mixture of lone-pair stereoactivity and inactivity is observed below that threshold. Brown (1988 ▸) correlated a vector-based measure of bond-length distortion to the Lewis base strength of the anion for Tl^+^ structures, and updated the threshold to 0.27 v.u. This threshold is set to include as many structures with lone-pair stereoactive cations without including structures where the lone pair is inert. Structures observed above that threshold typically do not form secondary bonds and have coordination numbers [3] and [4]. Although the model may not be used to predict lone-pair stereoactivity below the set threshold (most cases), it may be used to predict lone-pair stereoactivity above it, *i.e.* for structure with strong anion complexes.

Thus the Lewis acid–base argument is easy to apply, but is not always useful. Although the procedure is more involved, the occurrence of lone-pair stereoactivity is more confidently predicted *via* orbital energy calculations.

### 6.2. Mean bond-length distributions   

**Figure 7:**
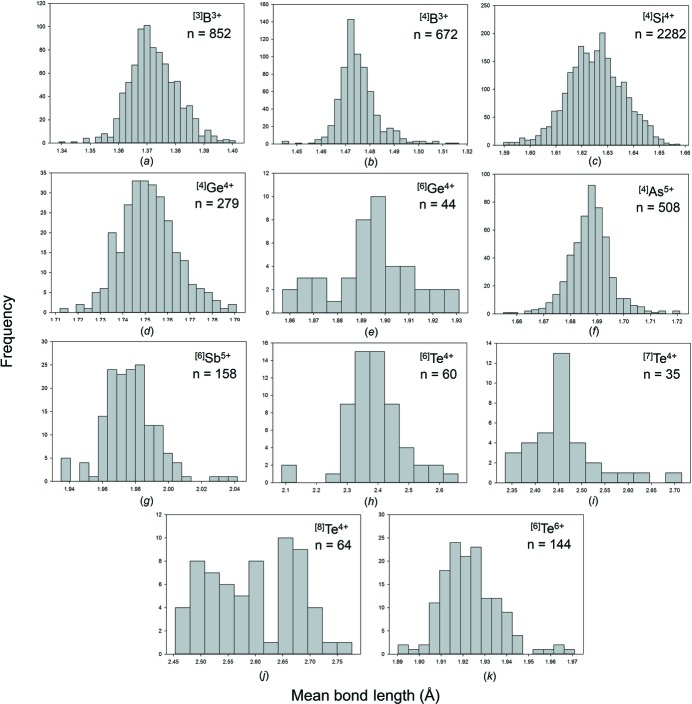
Mean bond-length distributions for selected configurations of the metalloid ions bonded to O^2−^: (*a*) ^[3]^B^3+^, (*b*) ^[4]^B^3+^, (*c*) ^[4]^Si^4+^, (*d*) ^[4]^Ge^4+^, (*e*) ^[6]^Ge^4+^, (*f*) ^[4]^As^5+^, (*g*) ^[6]^Sb^5+^, (*h*) ^[6]^Te^4+^, (*i*) ^[7]^Te^4+^, (*j*) ^[8]^Te^4+^, (*k*) ^[6]^Te^6+^.

The mean bond-length distributions for the metalloid and post-transition metal ions bonded to O^2−^ are given in Figs. S5 and S6, respectively. Those with adequate sample sizes (see sample size study above) are given in Figs. 7 ▸ and 8 ▸, and Tables 3 ▸ and 4 ▸ give the grand mean bond length (and standard deviation), the minimum and maximum mean bond length (and range), the skewness and kurtosis of these distributions (where justified by sample size) and the number of coordination polyhedra for all configurations observed.

Similar to the case for non-metal cations, both cations that form strongly bonded oxyanions (*e.g.* B^3+^, Si^4+^, As^5+^) and cations with ‘fully stereoactive’ lone-pair electrons (*i.e.* with coordination numbers [3] and [4]) have a narrow range of mean bond lengths, typically ∼0.06–0.09 Å. This range is larger for ion configurations with stereoactive lone-pair electrons where secondary bonds are formed (*e.g.* ^[5–12]^Bi^3+^), and is typically ∼0.1–0.3 Å for ion configurations with a dataset larger than ∼10 coordination polyhedra (mean bond-length range is highly dependent on sample size). The largest range of mean bond length observed is for ^[6]^Te^4+^ with 0.573 Å, followed by ^[7]^Te^4+^ with 0.380 Å and ^[8]^Te^4+^ with 0.323 Å; however, this may due to the relatively high occurrence of ion configurations in coordination numbers ∼[6]–[8]. In comparison, the largest mean bond-length range observed for non-metal ions with stereoactive lone-pair electrons bonded to O^2−^ is 0.227 Å for ^[6]^Se^4+^ (Gagné & Hawthorne, 2018 ▸), and the largest range for the alkali and alkaline earth metals bonded to O^2−^, respectively, are 0.652 Å for ^[6]^K^+^ and 0.436 Å for ^[10]^Sr^2+^; the mean bond-length ranges are typically ∼0.3–0.4 Å for these two families (Gagné & Hawthorne, 2016*a* ▸).

#### 6.2.1. Bond-length distortion   

**Figure 9:**
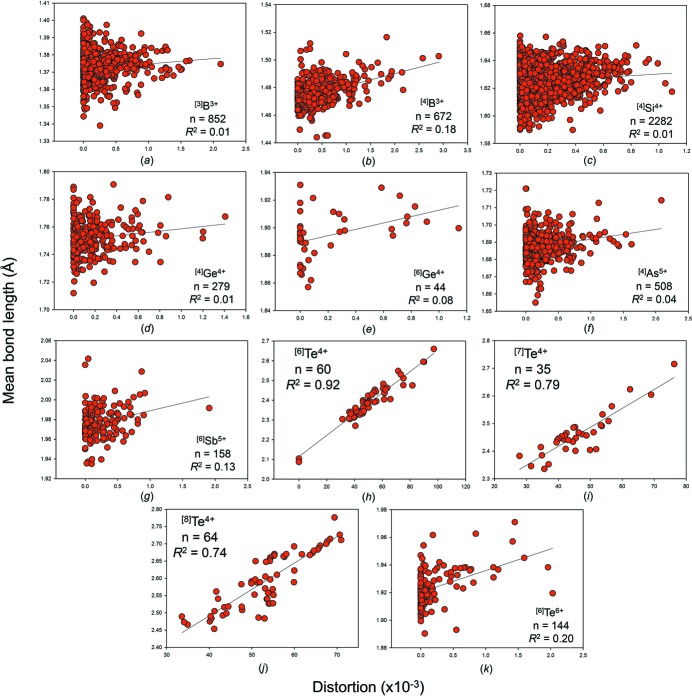
The effect of bond-length distortion on mean bond length for selected configurations of the metalloid ions bonded to O^2−^: (*a*) ^[3]^B^3+^, (*b*) ^[4]^B^3+^, (*c*) ^[4]^Si^4+^, (*d*) ^[4]^Ge^4+^, (*e*) ^[6]^Ge^4+^, (*f*) ^[4]^As^5+^, (*g*) ^[6]^Sb^5+^, (*h*) ^[6]^Te^4+^, (*i*) ^[7]^Te^4+^, (*j*) ^[8]^Te^4+^, (*k*) ^[6]^Te^6+^.

We give the bond-length distortion plots for the metalloid and post-transition metal ions bonded to O^2−^ in Figs. S7 and S8, and in Figs. 9 ▸ and 10 ▸ for those with adequate sample sizes. We use the definition of Brown & Shannon (1973 ▸) for distortion, *i.e.* the mean-square relative deviation of bond lengths from their average value. These plots show that mean bond length correlates highly with bond-length distortion for ion configurations observed with distortion values > 20 × 10^−3^, *e.g.* *R* ^2^ = 0.92 for ^[6]^Te^4+^, 0.86 for ^[8]^Bi^3+^, but correlates poorly below that. A similar threshold was observed at ∼10 × 10^−3^ for the non-metal ions with stereoactive lone-pair electrons (Gagné & Hawthorne, 2018 ▸).

#### 6.2.2. Factors affecting mean bond-length variations   

A thorough investigation of potential factors leading to mean bond-length variation was done by Gagné & Hawthorne (2017 ▸) for 55 ion configurations, which included analysis for ^[3]^B^3+^, ^[4]^B^3+^, ^[4]^Al^3+^, ^[6]^Al^3+^, ^[4]^Si^4+^, ^[4]^Ga^3+^, ^[4]^Ge^4+^, ^[4]^As^5+^, ^[6]^Sb^5+^ and ^[6]^Te^6+^. However, ion configurations with lone-pair electrons were not analyzed due to inadequate sample size. One of the conclusions of the study of Gagné & Hawthorne (2017 ▸) is that the well ingrained correlation between mean bond length and mean coordination number of the bonded anions, proposed in the late 1960s, in fact resulted from small sample size, and is not of general applicability to inorganic oxide and oxysalt structures. They also confirmed bond-length distortion as a causal factor of mean bond-length variation and quantified its effect, and found no statistically significant correlation between mean bond length and the mean electronegativity and mean ionization energy of the next-nearest neighbours.

Let us examine the results for the metalloid and post-transition metal ions: ^[3]^B^3+^ (*n* = 237 coordination polyhedra), ^[4]^B^3+^ (*n* = 148), ^[4]^Al^3+^ (*n* = 49), ^[6]^Al^3+^ (*n* = 58), ^[4]^Si^4+^ (*n* = 335), ^[4]^Ga^3+^ (*n* = 27), ^[4]^Ge^4+^ (*n* = 64), ^[4]^As^5+^ (*n* = 59), ^[6]^Sb^5+^ (*n* = 19) and ^[6]^Te^6+^ (*n* = 21). Student *t*-tests show that for (1) bond-length distortion, (2) mean coordination number of bonded anion, (3) mean electronegativity and (4) mean ionization energy of the next-nearest neighbours, there are 16 of 40 possible correlations that are significant at the 95% confidence level. For bond-length distortion, they are for (*R* ^2^) ^[4]^B^3+^(0.33), ^[6]^Al^3+^ (0.23), ^[6]^Sb^5+^ (0.45) and ^[6]^Te^6+^ (0.28); for mean coordination number of bonded anion, ^[3]^B^3+^ (0.10), ^[4]^B^3+^ (0.05), ^[4]^Al^3+^ (0.17), ^[6]^Al^3+^ (0.15) and ^[4]^Ga^3+^ (0.29); for mean electronegativity of the next-nearest neighbours, ^[4]^Ga^3+^ (−0.17), ^[4]^As^5+^ (0.02) and ^[6]^Te^6+^ (0.10); for mean ionization energy of the next-nearest neighbours, ^[4]^Si^4+^ (−0.08), ^[4]^Ga^3+^ (0.33), ^[4]^As^5+^ (−0.09) and ^[6]^Te^6+^ (−0.04). A negative symbol before *R* ^2^ indicates that the observed correlation with mean bond length is negative.

As discussed by Gagné & Hawthorne (2017 ▸), values of *R* ^2^ and *p*-values vary significantly as a function of sample size (*R* ^2^ values sometimes greater than 0.2 for sample sizes smaller than 100 coordination polyhedra for these variables), and although results for sample sizes > 35 coordination polyhedra are generally indicative, analysis of ion configurations with less than ∼100 coordination polyhedra cannot be considered statistically reliable. In the above case, the mean *R* ^2^ values for the four variables considered are (1) 0.32, (2) 0.15, (3) −0.02, and (4) 0.04. Based on (1) lack of statistical significance in most cases, (2) low *R* ^2^ values for those cases that are statistically significant, (3) the reliability of the *R* ^2^ values based on the sample size study of Gagné & Hawthorne (2017 ▸), and (4) a lack of demonstrated causality between mean bond length and these variables, we assume that mean bond length shows little or no correlation with the mean coordination number of bonded anion, the mean electronegativity of the next-nearest neighbours and the mean ionization energy of the next-nearest neighbours for the metalloid and post-transition metal ions.

The case for bond-length distortion is more interesting. It is clear from Figs. 9 ▸ and 10 ▸ that mean bond length is highly correlated to bond-length distortion for ion configurations that generally occur as highly distorted (*e.g.* ions with stereoactive lone-pair electrons), but correlates poorly otherwise. In addition, bond-length distortion is the only of the four potential factors analyzed above that has been demonstrated to be causal, *via* the distortion theorem (*e.g.* Brown & Shannon, 1973 ▸; Allmann, 1975 ▸; Brown, 1978 ▸; Urusov, 2003 ▸). Because of this, we can confidently say that bond-length distortion has a non-negligible effect on mean bond length for some strongly bonded metalloid and post-transition metal ions, and is the main cause of mean bond-length variation for highly distorted configurations of these ions.

Altogether, mean bond-length correlates poorly with the listed factors for the sample studied, and it is clear that one or more other factors affect mean bond-length variation. Following a study of *a priori* bond lengths in a variety of structures containing ^[4]^Al^3+^, ^[6]^Al^3+^ and ^[12]^Ba^2+^, Gagné & Hawthorne (2017 ▸) showed that *a priori* bond lengths do not correlate to observed bond lengths across structure types, although they are known to correlate well *within* structure types (*e.g.* *R* ^2^ > 0.99 for milarite; Gagné & Hawthorne, 2016*b* ▸). Following this, Gagné & Hawthorne (2017 ▸) proposed that the inability of crystal structures to attain their ideal (*a priori*) bond lengths within the constraints of space-group symmetry is the leading cause of mean bond-length variation in crystals. As we concluded in the previous article of this series for the oxyanions of non-metals (Gagné & Hawthorne, 2018 ▸), this phenomenon seems plausible in explaining the mean bond-length variations observed here, and should be investigated further.

## 7. Summary   

(1) We have examined the bond-length distributions for 33 configurations of the metalloid ions bonded to O^2−^ using 5279 coordination polyhedra and 21 761 bond distances, and for 56 configurations of the post-transition metal ions bonded to O^2−^ using 1821 coordination polyhedra and 10 723 bond distances.

(2) We find that for the seven metalloid and post-transition elements with lone-pair electrons we observe bonded to O^2−^, the most common state between their *n* *versus* *n*+2 oxidation states is that of higher oxidation state for As and Sb, and lower oxidation state for Sn, Te, Tl, Pb and Bi.

(3) We find no correlation between bond-valence sum and coordination number for cations with stereoactive lone-pair electrons using the bond-valence parameters of Gagné & Hawthorne (2015 ▸).

(4) We confirm the absence of a correlation between lone-pair stereoactivity and coordination number when including secondary bonds, whereby both intermediate states of lone-pair stereoactivity and inert lone pairs may be observed for any coordination number > [4] of a cation with lone-pair electrons.

(5) We observe variations in mean bond lengths of ∼0.06–0.09 Å for strongly bonded oxyanions of metalloid and post-transition metal ions, and ∼0.1–0.3 Å for these ions that display lone-pair stereoactivity.

(6) We show that bond-length distortion is a leading cause of mean bond-length variation for ions with stereoactive lone-pair electrons, and that the causes of mean bond-length variation for strongly bonded cations (*i.e.* oxyanions) remain unclear. The most probable cause of mean bond-length variation for these ions is the effect of structure type, *i.e.* stress produced by the inability of a structure to follow its *a priori* bond lengths.

## Supplementary Material

Figs S1 to S8. DOI: 10.1107/S2052520617017437/ra5031sup1.pdf

Raw data file for Al3+. DOI: 10.1107/S2052520617017437/ra5031sup2.txt

Raw data file for As3+. DOI: 10.1107/S2052520617017437/ra5031sup3.txt

Raw data file for As5+. DOI: 10.1107/S2052520617017437/ra5031sup4.txt

Raw data file for B3+. DOI: 10.1107/S2052520617017437/ra5031sup5.txt

Raw data file for Bi3+. DOI: 10.1107/S2052520617017437/ra5031sup6.txt

Raw data file for Bi5+. DOI: 10.1107/S2052520617017437/ra5031sup7.txt

Raw data file for Ga3+. DOI: 10.1107/S2052520617017437/ra5031sup8.txt

Raw data file for Ge4+. DOI: 10.1107/S2052520617017437/ra5031sup9.txt

Raw data file for In3+. DOI: 10.1107/S2052520617017437/ra5031sup10.txt

Raw data file for Pb2+. DOI: 10.1107/S2052520617017437/ra5031sup11.txt

Raw data file for Pb4+. DOI: 10.1107/S2052520617017437/ra5031sup12.txt

Raw data file for Sb3+. DOI: 10.1107/S2052520617017437/ra5031sup13.txt

Raw data file for Sb5+. DOI: 10.1107/S2052520617017437/ra5031sup14.txt

Raw data file for Sb5+. DOI: 10.1107/S2052520617017437/ra5031sup14.txt

Raw data file for Si4+. DOI: 10.1107/S2052520617017437/ra5031sup15.txt

Raw data file for Sn2+. DOI: 10.1107/S2052520617017437/ra5031sup16.txt

Raw data file for Sn4+. DOI: 10.1107/S2052520617017437/ra5031sup17.txt

Raw data file for Te4+. DOI: 10.1107/S2052520617017437/ra5031sup18.txt

Raw data file for Te6+. DOI: 10.1107/S2052520617017437/ra5031sup19.txt

Raw data file for Tl3+. DOI: 10.1107/S2052520617017437/ra5031sup20.txt

Raw data file for Tl+. DOI: 10.1107/S2052520617017437/ra5031sup21.txt

## Figures and Tables

**Figure 8:**
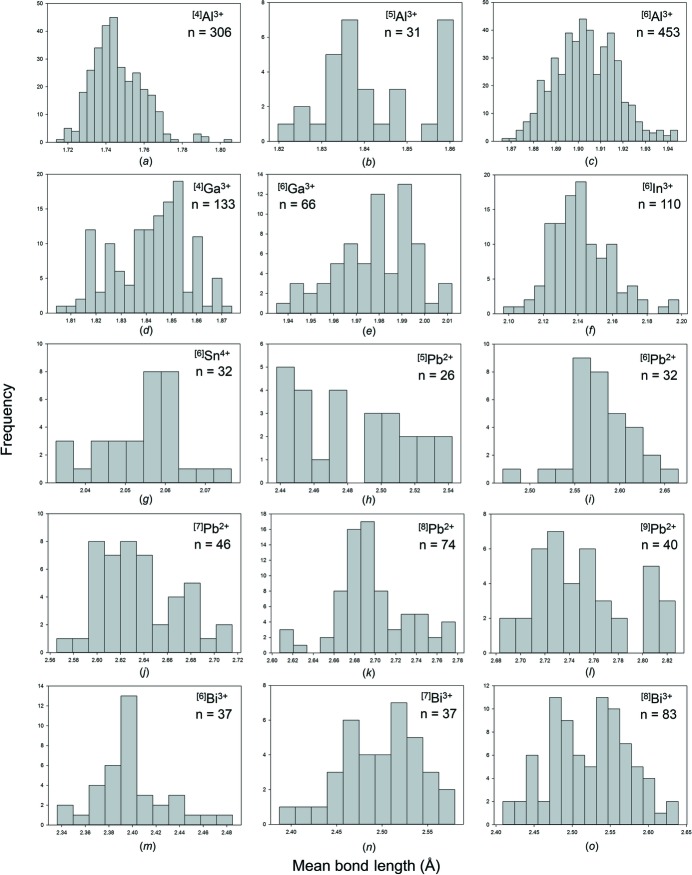
Mean bond-length distributions for selected configurations of the post-transition metal ions bonded to O^2−^: (*a*) ^[4]^Al^3+^, (*b*) ^[5]^Al^3+^, (*c*) ^[6]^Al^3+^, (*d*) ^[4]^Ga^3+^, (*e*) ^[6]^Ga^3+^, (*f*) ^[6]^In^3+^, (*g*) ^[6]^Sn^4+^, (*h*) ^[5]^Pb^2+^, (*i*) ^[6]^Pb^2+^, (*j*) ^[7]^Pb^2+^, (*k*) ^[8]^Pb^2+^, (*l*) ^[9]^Pb^2+^, (*m*) ^[6]^Bi^3+^, (*n*) ^[7]^Bi^3+^, (*o*) ^[8]^Bi^3+^.

**Figure 10:**
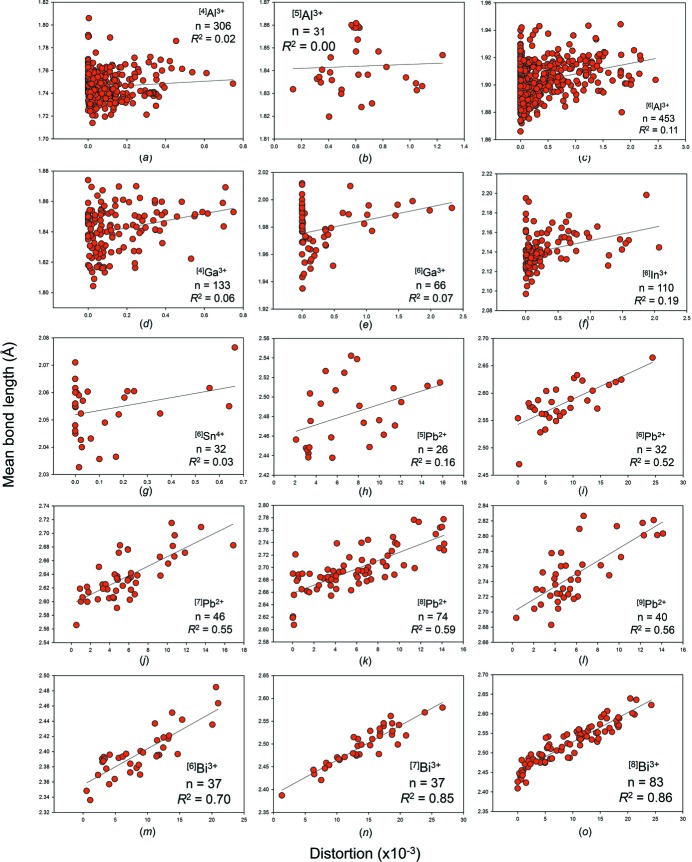
The effect of bond-length distortion on mean bond length for selected configurations of the post-transition metal ions bonded to O^2−^: (*a*) ^[4]^Al^3+^, (*b*) ^[5]^Al^3+^, (*c*) ^[6]^Al^3+^, (*d*) ^[4]^Ga^3+^, (*e*) ^[6]^Ga^3+^, (*f*) ^[6]^In^3+^, (*g*) ^[6]^Sn^4+^, (*h*) ^[5]^Pb^2+^, (*i*) ^[6]^Pb^2+^, (*j*) ^[7]^Pb^2+^, (*k*) ^[8]^Pb^2+^, (*l*) ^[9]^Pb^2+^, (*m*) ^[6]^Bi^3+^, (*n*) ^[7]^Bi^3+^, (*o*) ^[8]^Bi^3+^.

**Table 1:** Bond-length statistics for the metalloid ions bonded to O^2−^

Ion	Coordination number	Number of bonds	Number of coordination polyhedra	Mean bond length (Å)	Standard deviation (Å)	Range (Å)	Maximum bond length (Å)	Minimum bond length (Å)	Skewness	Kurtosis
B^3+^	3	2556	852	1.372	0.021	0.166	1.464	1.298	0.2	1.0
	4	2688	672	1.475	0.028	0.236	1.616	1.380	0.6	1.4
Si^4+^	4	9128	2282	1.625	0.024	0.166	1.726	1.560	0.0	0.0
	6	144	24	1.783	0.028	0.197	1.903	1.706	–	–
Ge^4+^	4	1116	279	1.752	0.027	0.179	1.859	1.680	0.4	0.6
	5	60	12	1.847	0.080	0.398	2.117	1.719	–	–
	6	264	44	1.894	0.033	0.177	1.995	1.818	–	–
As^3+^	3	27	9	1.776	0.045	0.174	1.845	1.671	–	–
	4	16	4	2.026	0.406	1.177	2.896	1.719	–	–
	5	40	8	2.247	0.553	1.338	3.092	1.754	0.5	−1.8
	6	36	6	2.410	0.620	1.492	3.212	1.720	0.1	−2.0
	8	8	1	2.480	0.507	1.201	3.005	1.804	–	–
As^5+^	4	2032	508	1.687	0.027	0.196	1.806	1.610	0.7	1.5
	6	78	13	1.830	0.028	0.121	1.888	1.767	–	–
Sb^3+^	3	18	6	1.932	0.024	0.083	1.982	1.899	–	–
	4	60	15	2.092	0.178	0.698	2.596	1.898	1.3	1.0
	5	35	7	2.240	0.309	1.018	2.963	1.945	1.2	0.4
	6	72	12	2.443	0.485	1.474	3.391	1.917	0.4	−1.5
	7	49	7	2.486	0.451	1.308	3.266	1.958	0.3	−1.5
	8	32	4	2.584	0.477	1.386	3.362	1.976	–	–
	9	9	1	2.758	0.583	1.404	3.385	1.981	–	–
Sb^5+^	6	948	158	1.977	0.034	0.208	2.102	1.894	0.7	1.0
Te^4+^	3	12	4	1.843	0.015	0.043	1.862	1.819	–	–
	4	28	7	1.984	0.123	0.329	2.176	1.847	0.2	−1.9
	5	80	16	2.251	0.511	1.773	3.586	1.813	1.2	0.0
	6	360	60	2.386	0.552	1.786	3.595	1.809	0.5	−1.3
	7	245	35	2.460	0.538	1.747	3.556	1.809	0.2	−1.6
	8	512	64	2.594	0.608	1.805	3.615	1.810	0.1	−1.6
	9	126	14	2.677	0.611	1.830	3.674	1.844	−0.2	−1.5
	10	60	6	2.833	0.692	1.873	3.736	1.863	−0.3	−1.6
	11	22	2	2.812	0.668	1.699	3.581	1.882	–	–
	12	36	3	2.928	0.643	1.853	3.697	1.844	–	–
Te^6+^	6	864	144	1.923	0.030	0.231	2.048	1.817	0.7	3.5

**Table 2:** Bond-length statistics for the post-transition metal ions bonded to O^2−^

Ion	Coordination number	Number of bonds	Number of coordination polyhedra	Mean bond length (Å)	Standard deviation (Å)	Range (Å)	Maximum bond length (Å)	Minimum bond length (Å)	Skewness	Kurtosis
Al^3+^	4	1224	306	1.746	0.022	0.142	1.827	1.685	0.2	0.3
	5	155	31	1.842	0.047	0.190	1.938	1.748	–	–
	6	2718	453	1.903	0.040	0.262	2.054	1.792	0.6	0.7
Ga^3+^	4	532	133	1.842	0.027	0.174	1.948	1.774	0.5	0.4
	5	90	18	1.910	0.076	0.483	2.254	1.771	–	–
	6	396	66	1.978	0.040	0.237	2.130	1.893	0.8	1.7
In^3+^	6	660	110	2.142	0.042	0.301	2.324	2.023	0.9	1.8
	7	28	4	2.218	0.071	0.365	2.450	2.085	–	–
	8	16	2	2.275	0.058	0.155	2.351	2.196	–	–
Sn^2+^	3	63	21	2.094	0.031	0.158	2.162	2.004	–	–
	4	24	6	2.281	0.225	0.818	2.881	2.063	1.3	1.1
	5	30	6	2.469	0.402	1.166	3.228	2.062	0.6	−1.5
	6	54	9	2.508	0.310	1.183	3.251	2.068	0.6	−0.7
	7	14	2	2.690	0.488	1.345	3.407	2.062	–	–
	8	40	5	2.706	0.423	1.212	3.327	2.115	0.1	−1.7
	9	9	1	2.828	0.490	1.155	3.312	2.157	–	–
Sn^4+^	4	16	4	1.956	0.009	0.035	1.970	1.935	–	–
	6	192	32	2.054	0.024	0.134	2.130	1.996	–	–
	7	7	1	2.115	0.027	0.070	2.142	2.072	–	–
Tl^+^	3	9	3	2.517	0.051	0.157	2.569	2.412	–	–
	4	12	3	2.726	0.231	0.867	3.245	2.378	–	–
	5	15	3	2.840	0.296	0.864	3.356	2.492	–	–
	6	48	8	2.887	0.194	0.816	3.271	2.455	0.0	−0.6
	7	56	8	2.976	0.226	0.790	3.366	2.576	−0.1	−1.4
	8	96	12	2.988	0.192	0.804	3.363	2.559	0.2	−0.7
	9	99	11	2.991	0.192	0.846	3.404	2.558	0.2	−0.8
	10	110	11	3.102	0.220	0.979	3.626	2.647	0.4	−0.6
	11	55	5	3.134	0.258	0.998	3.674	2.676	0.4	−0.6
	12	108	9	3.195	0.285	1.290	4.012	2.722	0.7	−0.3
Tl^3+^	6	36	6	2.228	0.076	0.407	2.481	2.074	–	–
	7	7	1	2.336	0.157	0.504	2.624	2.120	–	–
	8	16	2	2.378	0.138	0.618	2.804	2.186	–	–
Pb^2+^	3	15	5	2.210	0.047	0.142	2.291	2.149	0.7	−1.1
	4	68	17	2.357	0.108	0.464	2.638	2.174	0.5	0.1
	5	130	26	2.482	0.212	0.843	2.990	2.147	0.4	−1.0
	6	192	32	2.581	0.237	0.984	3.153	2.169	0.2	−0.7
	7	322	46	2.637	0.205	1.089	3.214	2.125	0.1	−0.2
	8	592	74	2.697	0.210	1.048	3.280	2.232	0.4	−0.3
	9	360	40	2.750	0.217	0.950	3.231	2.281	0.2	−0.7
	10	140	14	2.789	0.212	0.970	3.261	2.291	0.1	−0.7
	11	44	4	2.838	0.205	0.767	3.168	2.401	–	–
	12	204	17	2.827	0.113	0.722	3.200	2.478	0.4	1.9
Pb^4+^	4	8	2	2.056	0.022	0.076	2.074	1.998	–	–
	5	5	1	2.147	0.100	0.276	2.324	2.048	–	–
	6	54	9	2.169	0.038	0.168	2.237	2.069	–	–
Bi^3+^	3	27	9	2.069	0.037	0.130	2.151	2.021	–	–
	4	84	21	2.211	0.163	0.805	2.790	1.985	0.9	1.1
	5	90	18	2.317	0.195	0.732	2.785	2.053	0.5	−0.7
	6	222	37	2.398	0.229	0.904	2.981	2.077	0.6	−0.7
	7	259	37	2.497	0.303	1.097	3.154	2.057	0.4	−1.2
	8	664	83	2.522	0.252	1.069	3.165	2.096	0.5	−0.5
	9	162	18	2.604	0.302	1.081	3.158	2.077	0.1	−1.2
	10	70	7	2.670	0.313	1.093	3.219	2.126	0.0	−1.0
	12	12	1	2.671	0.264	0.897	3.183	2.286	–	–
Bi^5+^	4	4	1	1.979	0.015	0.038	2.002	1.964	–	–
	6	60	10	2.110	0.033	0.165	2.174	2.009	–	–

**Table 3:** Mean bond-length statistics for the metalloid ions bonded to O^2−^

Ion	Coordination number	Number of coordination polyhedra	Grand mean bond length (Å)	Standard deviation (Å)	Mean bond-length range (Å)	Maximum mean bond length (Å)	Minimum mean bond length (Å)	Skewness	Kurtosis
B^3+^	3	852	1.372	0.008	0.062	1.401	1.339	0.2	0.5
	4	672	1.475	0.008	0.073	1.517	1.444	0.9	3.9
Si^4+^	4	2282	1.625	0.011	0.068	1.658	1.590	−0.1	0.0
	6	24	1.783	0.017	0.064	1.819	1.755	–	–
Ge^4+^	4	279	1.752	0.013	0.079	1.791	1.712	0.3	0.4
	5	12	1.847	0.013	0.040	1.868	1.828	–	–
	6	44	1.894	0.018	0.074	1.931	1.857	–	–
As^3+^	3	9	1.776	0.011	0.036	1.794	1.758	–	–
	4	4	2.026	0.036	0.081	2.077	1.996	–	–
	5	8	2.247	0.025	0.082	2.295	2.213	–	–
	6	6	2.410	0.060	0.163	2.495	2.332	–	–
	8	1	2.480	–	0.000	2.480	2.480	–	–
As^5+^	4	508	1.687	0.008	0.066	1.721	1.655	0.2	2.4
	6	13	1.830	0.008	0.031	1.849	1.819	–	–
Sb^3+^	3	6	1.932	0.022	0.060	1.974	1.914	–	–
	4	15	2.092	0.025	0.064	2.125	2.061	–	–
	5	7	2.240	0.063	0.182	2.361	2.179	–	–
	6	12	2.443	0.083	0.274	2.623	2.349	–	–
	7	7	2.486	0.023	0.072	2.517	2.445	–	–
	8	4	2.584	0.032	0.074	2.626	2.552	–	–
	9	1	2.758	–	0.000	2.758	2.758	–	–
Sb^5+^	6	158	1.977	0.016	0.107	2.042	1.935	–	–
Te^4+^	3	4	1.843	0.017	0.039	1.858	1.819	–	–
	4	7	1.984	0.011	0.042	2.006	1.964	–	–
	5	16	2.251	0.085	0.271	2.383	2.112	–	–
	6	60	2.386	0.096	0.573	2.660	2.087	–	–
	7	35	2.460	0.077	0.380	2.715	2.336	–	–
	8	64	2.594	0.082	0.323	2.776	2.453	–	–
	9	14	2.677	0.073	0.250	2.778	2.528	–	–
	10	6	2.833	0.085	0.203	2.921	2.718	–	–
	11	2	2.812	0.032	0.045	2.835	2.790	–	–
	12	3	2.928	0.102	0.193	3.044	2.851	–	–
Te^6+^	6	144	1.923	0.013	0.081	1.971	1.890	–	–

**Table 4:** Mean bond-length statistics for the post-transition metal ions bonded to O^2−^

Ion	Coordination number	Number of coordination polyhedra	Grand mean bond length (Å)	Standard deviation (Å)	Mean bond-length range (Å)	Maximum mean bond length (Å)	Minimum mean bond length (Å)	Skewness	Kurtosis
Al^3+^	4	306	1.746	0.013	0.092	1.806	1.714	0.9	1.7
	5	31	1.842	0.012	0.041	1.861	1.820	–	–
	6	453	1.903	0.014	0.078	1.944	1.866	0.2	−0.1
Ga^3+^	4	133	1.842	0.015	0.070	1.874	1.804	–	–
	5	18	1.910	0.014	0.048	1.941	1.893	–	–
	6	66	1.978	0.017	0.077	2.012	1.935	–	–
In^3+^	6	110	2.142	0.018	0.101	2.198	2.097	–	–
	7	4	2.218	0.014	0.030	2.238	2.208	–	–
	8	2	2.275	0.002	0.003	2.276	2.274	–	–
Sn^2+^	3	21	2.094	0.017	0.062	2.121	2.059	–	–
	4	6	2.281	0.024	0.069	2.315	2.246	–	–
	5	6	2.451	0.049	0.125	2.492	2.367	–	–
	6	9	2.508	0.064	0.174	2.577	2.403	–	–
	7	2	2.690	0.015	0.021	2.701	2.680	–	–
	8	5	2.706	0.013	0.030	2.722	2.692	–	–
	9	1	2.828	–	0.000	2.828	2.828	–	–
	4	4	1.956	0.002	0.004	1.958	1.954	–	–
Sn^4+^	6	32	2.054	0.010	0.044	2.077	2.033	–	–
	7	1	2.115	–	0.000	2.115	2.115	–	–
Tl^+^	3	3	2.517	0.035	0.070	2.551	2.481	–	–
	4	3	2.726	0.049	0.097	2.773	2.676	–	–
	5	3	2.840	0.055	0.108	2.889	2.781	–	–
	6	8	2.887	0.032	0.089	2.922	2.833	–	–
	7	8	2.976	0.030	0.094	3.012	2.918	–	–
	8	12	2.976	0.030	0.094	3.012	2.918	–	–
	9	11	2.991	0.030	0.095	3.049	2.954	–	–
	10	11	3.102	0.059	0.166	3.203	3.037	–	–
	11	5	3.134	0.043	0.113	3.176	3.062	–	–
	12	9	3.195	0.073	0.210	3.304	3.094	–	–
Tl^3+^	6	6	2.228	0.013	0.036	2.242	2.206	–	–
	7	1	2.336	–	0.000	2.336	2.336	–	–
	8	2	2.378	0.047	0.067	2.412	2.345	–	–
Pb^2+^	3	5	2.210	0.038	0.083	2.250	2.167	–	–
	4	17	2.357	0.030	0.119	2.413	2.294	–	–
	5	26	2.482	0.033	0.104	2.542	2.438	–	–
	6	32	2.581	0.037	0.195	2.665	2.470	–	–
	7	46	2.637	0.033	0.149	2.715	2.566	–	–
	8	74	2.697	0.036	0.170	2.778	2.608	0.2	0.5
	9	40	2.750	0.038	0.144	2.827	2.683	–	–
	10	14	2.789	0.028	0.100	2.840	2.740	–	–
	11	4	2.821	0.046	0.111	2.869	2.758	–	–
	12	17	2.827	0.030	0.094	2.867	2.773	–	–
Pb^4+^	4	2	2.056	0.007	0.011	2.061	2.051	–	–
	5	1	2.147	–	0.000	2.147	2.147	–	–
	6	9	2.169	0.014	0.044	2.193	2.149	–	–
Bi^3+^	3	9	2.069	0.021	0.060	2.096	2.036	–	–
	4	21	2.211	0.024	0.095	2.268	2.173	–	–
	5	18	2.317	0.033	0.098	2.369	2.271	–	–
	6	37	2.398	0.031	0.149	2.485	2.336	–	–
	7	37	2.497	0.043	0.193	2.580	2.387	–	–
	8	83	2.522	0.052	0.230	2.639	2.409	0.0	−0.6
	9	18	2.604	0.052	0.149	2.654	2.505	–	–
	10	7	2.670	0.040	0.116	2.704	2.588	–	–
	12	1	2.671	–	0.000	2.671	2.671	–	–
Bi^5+^	4	1	1.979	–	0.000	1.979	1.979	–	–
	6	10	2.110	0.013	0.038	2.130	2.092	–	–
