# Supplementary material for: Bond-length distributions for ions bonded to oxygen: metalloids and post-transition metals
Source: Acta Crystallogr B Struct Sci Cryst Eng Mater. 2018 Jan 12;74(Pt 1):63–78. doi: 10.1107/S2052520617017437 (PMC5798398; doi:10.1107/S2052520617017437)
Supplement: Supplementary file 1 [file b-74-00063-sup1.pdf]

Acta Cryst B
STRUCTURAL SCIENCE
CRYSTAL ENGINEERING
MATERIALS

Volume 74 (2018)

Supporting information for article:

Bond-length distributions for ions bonded to oxygen: metalloids and post-transition metals

Olivier Charles Gagné and Frank Christopher Hawthorne

Figure S1 Bond-length distributions for all configurations of the metalloid ions bonded to O^{2-}: (*a*) $^{[3]}B^{3+}$, (*b*) $^{[4]}B^{3+}$, (*c*) $^{[4]}Si^{4+}$, (*d*) $^{[6]}Si^{4+}$, (*e*) $^{[4]}Ge^{4+}$, (*f*) $^{[5]}Ge^{4+}$, (*g*) $^{[6]}Ge^{4+}$, (*h*) $^{[3]}As^{3+}$, (*i*) $^{[4]}As^{3+}$, (*j*) $^{[5]}As^{3+}$, (*k*) $^{[6]}As^{3+}$, (*l*) $^{[8]}As^{3+}$, (*m*) $^{[4]}As^{5+}$, (*n*) $^{[6]}As^{5+}$, (*o*) $^{[3]}Sb^{3+}$, (*p*) $^{[4]}Sb^{3+}$, (*q*) $^{[5]}Sb^{3+}$, (*r*) $^{[6]}Sb^{3+}$, (*s*) $^{[7]}Sb^{3+}$, (*t*) $^{[8]}Sb^{3+}$, (*u*) $^{[9]}Sb^{3+}$, (*v*) $^{[6]}Sb^{5+}$, (*w*) $^{[3]}Te^{4+}$, (*x*) $^{[4]}Te^{4+}$, (*y*) $^{[5]}Te^{4+}$, (*z*) $^{[6]}Te^{4+}$, (*aa*) $^{[7]}Te^{4+}$, (*ab*) $^{[8]}Te^{4+}$, (*ac*) $^{[9]}Te^{4+}$, (*ad*) $^{[10]}Te^{4+}$, (*ae*) $^{[11]}Te^{4+}$, (*af*) $^{[12]}Te^{4+}$, (*ag*) $^{[6]}Te^{6+}$.

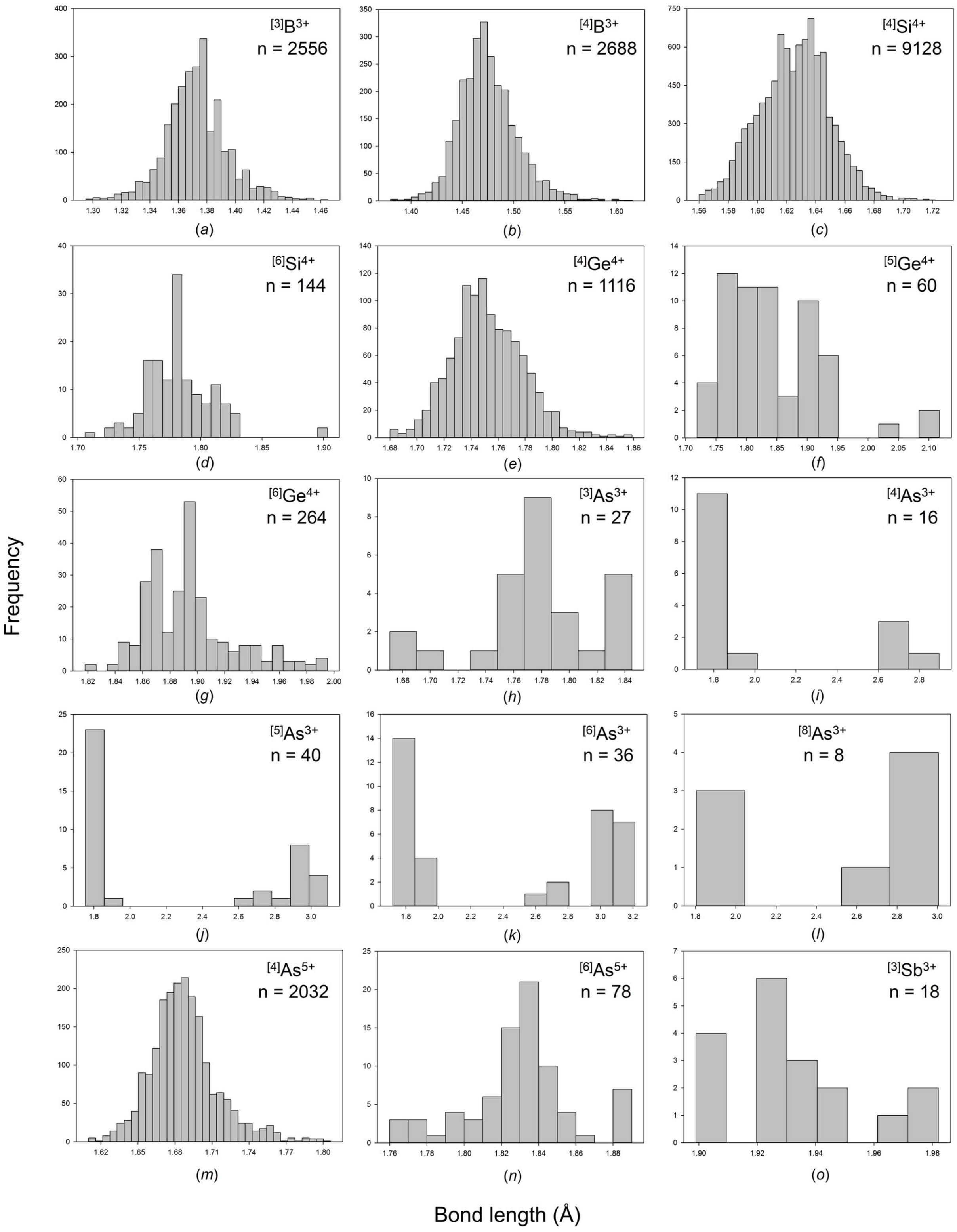

[3]B3+ n = 2556
(a)
[4]B3+ n = 2688
(b)
[4]Si4+ n = 9128
(c)
[6]Si4+ n = 144
(d)
[4]Ge4+ n = 1116
(e)
[5]Ge4+ n = 60
(f)
[6]Ge4+ n = 264
(g)
[3]As3+ n = 27
(h)
[4]As3+ n = 16
(i)
[5]As3+ n = 40
(j)
[6]As3+ n = 36
(k)
[8]As3+ n = 8
(l)
[4]As5+ n = 2032
(m)
[6]As5+ n = 78
(n)
[3]Sb3+ n = 18
(o)
Frequency
Bond length (Å)

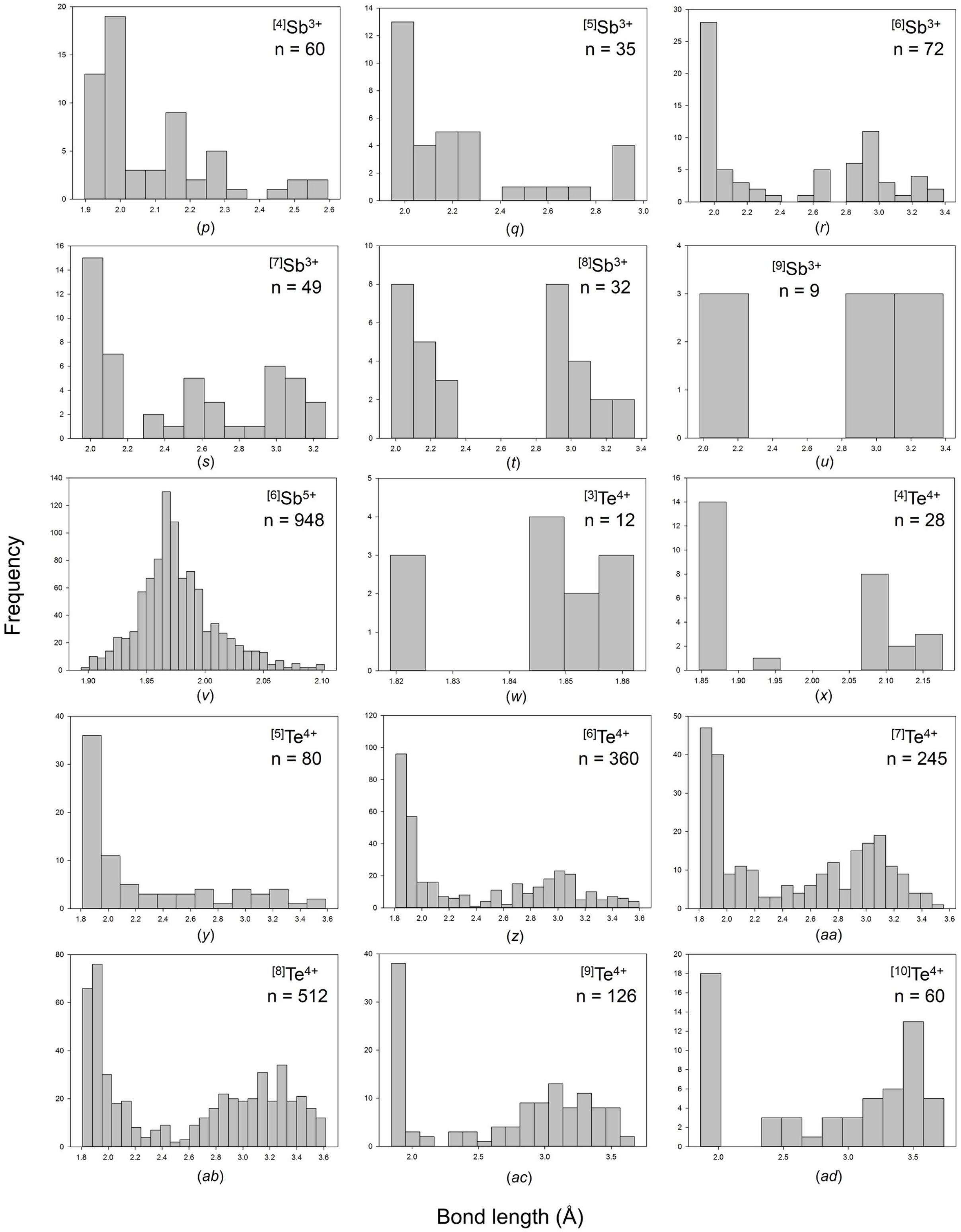
[4]Sb3+
n = 60
(p)
[5]Sb3+
n = 35
(q)
[6]Sb3+
n = 72
(r)
[7]Sb3+
n = 49
(s)
[8]Sb3+
n = 32
(t)
[9]Sb3+
n = 9
(u)
[6]Sb5+
n = 948
(v)
[3]Te4+
n = 12
(w)
[4]Te4+
n = 28
(x)
[5]Te4+
n = 80
(y)
[6]Te4+
n = 360
(z)
[7]Te4+
n = 245
(aa)
[8]Te4+
n = 512
(ab)
[9]Te4+
n = 126
(ac)
[10]Te4+
n = 60
(ad)
Frequency
Bond length (Å)

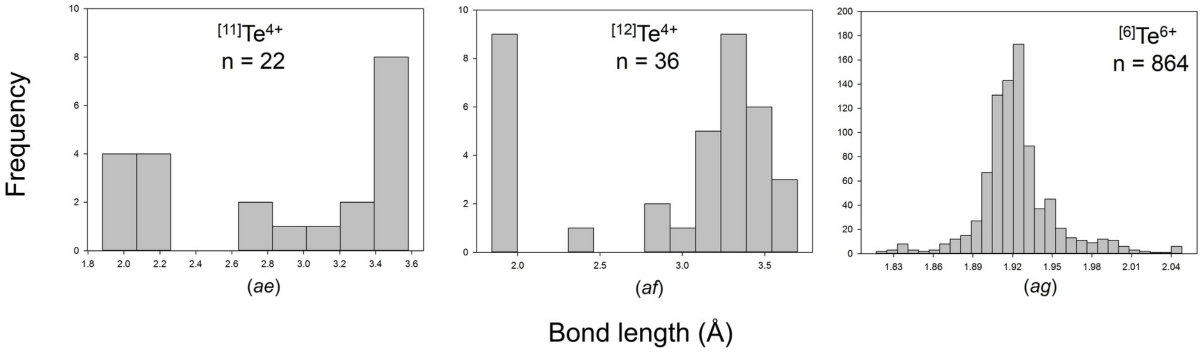
Frequency
[11]Te4+
n = 22
(ae)
[12]Te4+
n = 36
(af)
[6]Te6+
n = 864
(ag)
Bond length (Å)

Figure S2 Bond-valence distributions for all configurations of the metalloid ions bonded to O^{2-}: (*a*) $^{[3]}B^{3+}$, (*b*) $^{[4]}B^{3+}$, (*c*) $^{[4]}Si^{4+}$, (*d*) $^{[6]}Si^{4+}$, (*e*) $^{[4]}Ge^{4+}$, (*f*) $^{[5]}Ge^{4+}$, (*g*) $^{[6]}Ge^{4+}$, (*h*) $^{[3]}As^{3+}$, (*i*) $^{[4]}As^{3+}$, (*j*) $^{[5]}As^{3+}$, (*k*) $^{[6]}As^{3+}$, (*l*) $^{[8]}As^{3+}$, (*m*) $^{[4]}As^{5+}$, (*n*) $^{[6]}As^{5+}$, (*o*) $^{[3]}Sb^{3+}$, (*p*) $^{[4]}Sb^{3+}$, (*q*) $^{[5]}Sb^{3+}$, (*r*) $^{[6]}Sb^{3+}$, (*s*) $^{[7]}Sb^{3+}$, (*t*) $^{[8]}Sb^{3+}$, (*u*) $^{[9]}Sb^{3+}$, (*v*) $^{[6]}Sb^{5+}$, (*w*) $^{[3]}Te^{4+}$, (*x*) $^{[4]}Te^{4+}$, (*y*) $^{[5]}Te^{4+}$, (*z*) $^{[6]}Te^{4+}$, (*aa*) $^{[7]}Te^{4+}$, (*ab*) $^{[8]}Te^{4+}$, (*ac*) $^{[9]}Te^{4+}$, (*ad*) $^{[10]}Te^{4+}$, (*ae*) $^{[11]}Te^{4+}$, (*af*) $^{[12]}Te^{4+}$, (*ag*) $^{[6]}Te^{6+}$.

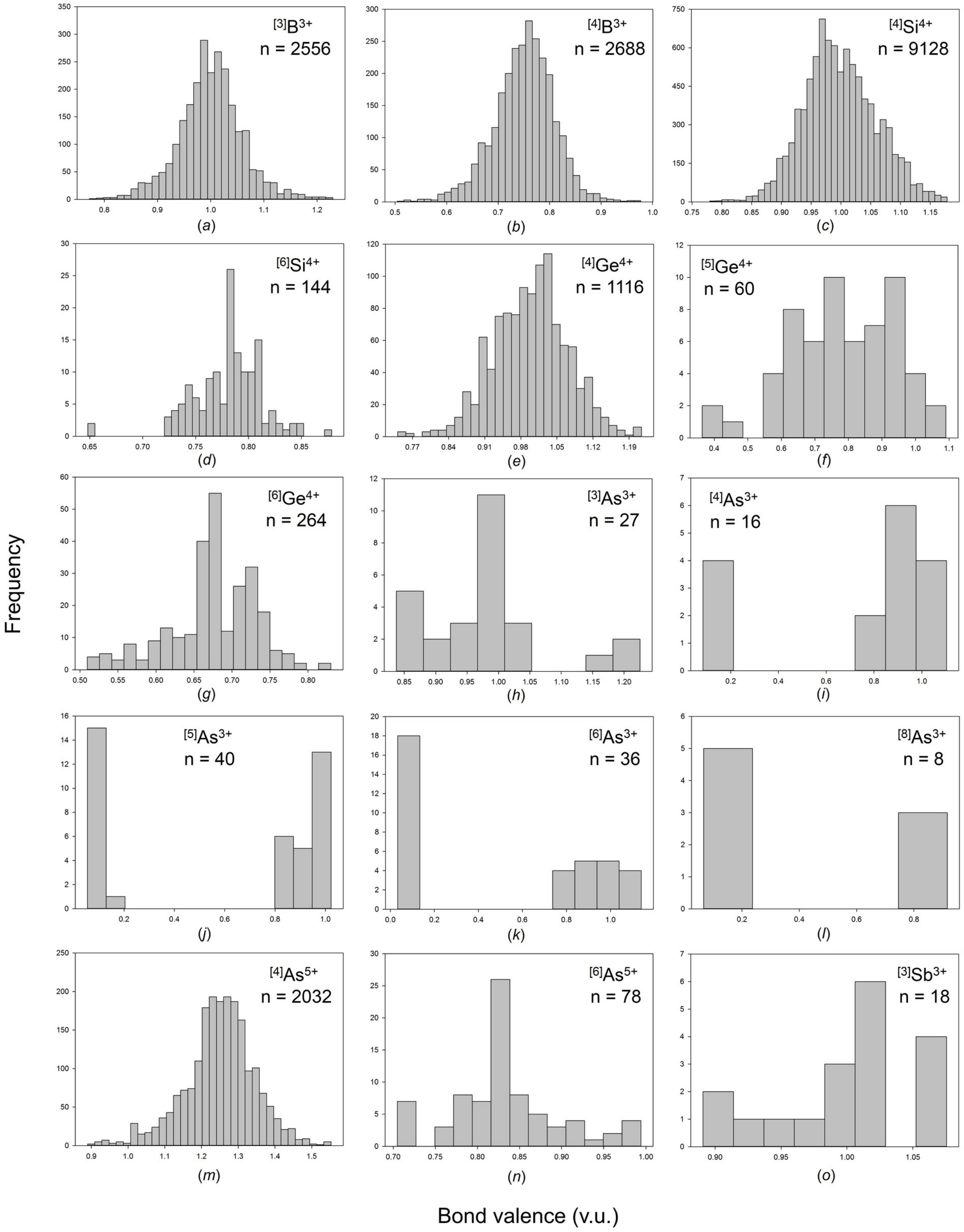
$^{[3]}B^{3+}$
n = 2556
(*a*)
$^{[4]}B^{3+}$
n = 2688
(*b*)
$^{[4]}Si^{4+}$
n = 9128
(*c*)
$^{[6]}Si^{4+}$
n = 144
(*d*)
$^{[4]}Ge^{4+}$
n = 1116
(*e*)
$^{[5]}Ge^{4+}$
n = 60
(*f*)
$^{[6]}Ge^{4+}$
n = 264
(*g*)
$^{[3]}As^{3+}$
n = 27
(*h*)
$^{[4]}As^{3+}$
n = 16
(*i*)
$^{[5]}As^{3+}$
n = 40
(*j*)
$^{[6]}As^{3+}$
n = 36
(*k*)
$^{[8]}As^{3+}$
n = 8
(*l*)
$^{[4]}As^{5+}$
n = 2032
(*m*)
$^{[6]}As^{5+}$
n = 78
(*n*)
$^{[3]}Sb^{3+}$
n = 18
(*o*)
Frequency
Bond valence (v.u.)

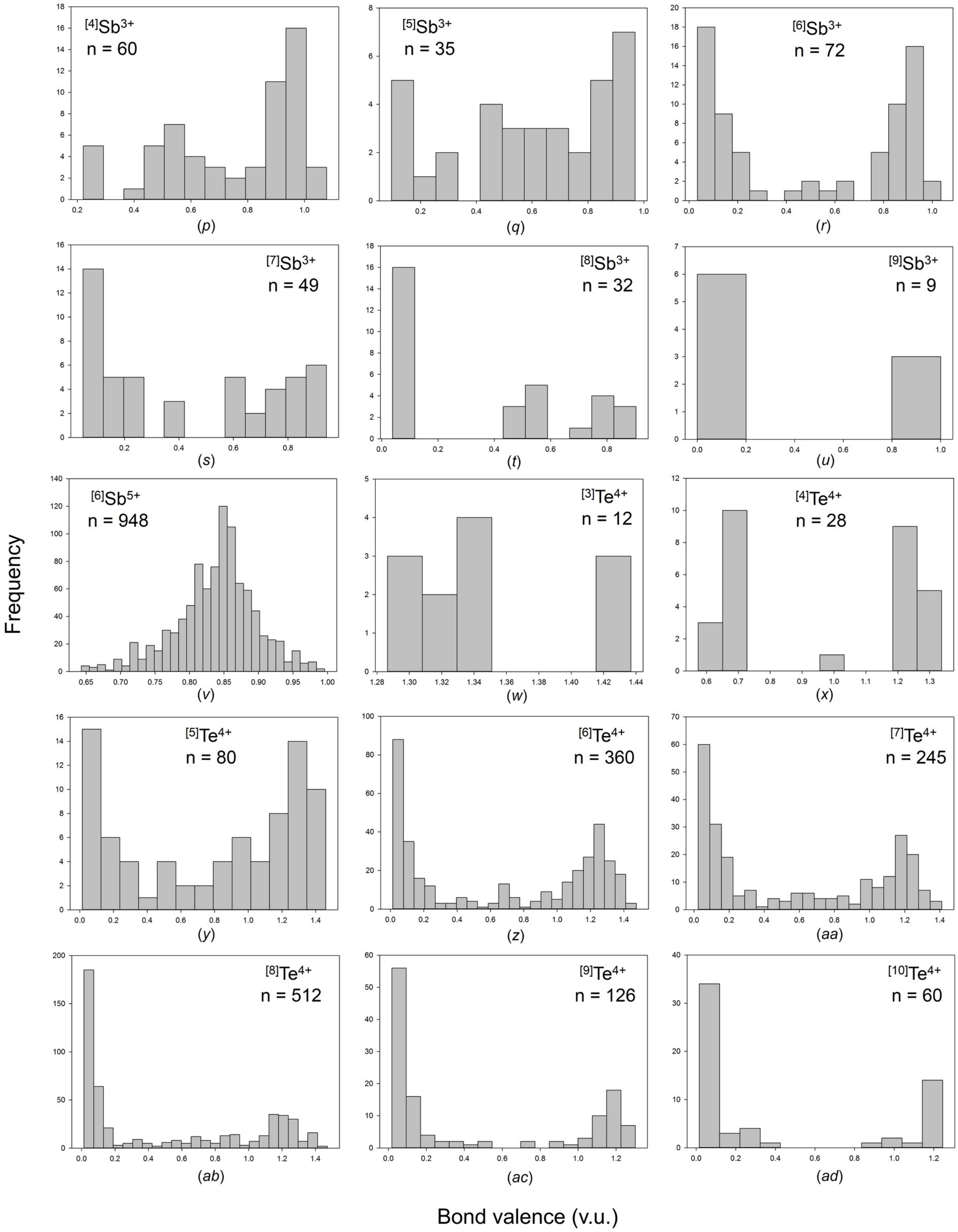
[4]Sb3+
n = 60
(p)
[5]Sb3+
n = 35
(q)
[6]Sb3+
n = 72
(r)
[7]Sb3+
n = 49
(s)
[8]Sb3+
n = 32
(t)
[9]Sb3+
n = 9
(u)
[6]Sb5+
n = 948
(v)
[3]Te4+
n = 12
(w)
[4]Te4+
n = 28
(x)
[5]Te4+
n = 80
(y)
[6]Te4+
n = 360
(z)
[7]Te4+
n = 245
(aa)
[8]Te4+
n = 512
(ab)
[9]Te4+
n = 126
(ac)
[10]Te4+
n = 60
(ad)
Frequency
Bond valence (v.u.)

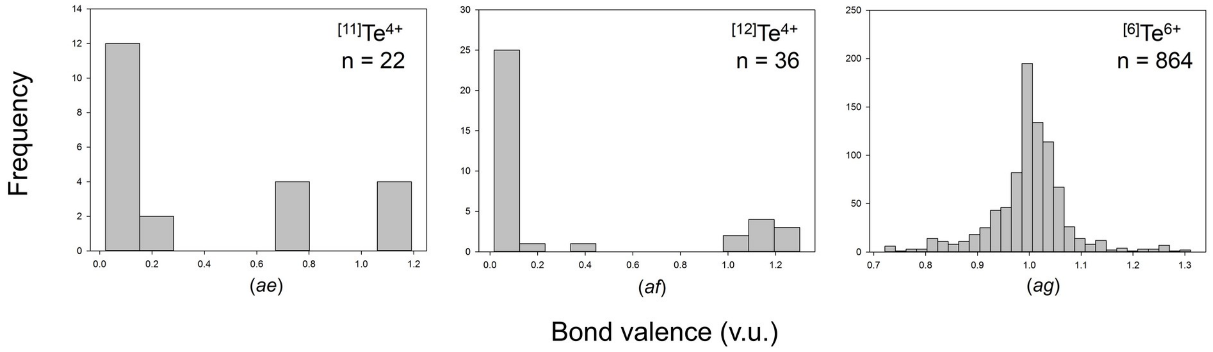
[11]Te4+
n = 22
[12]Te4+
n = 36
[6]Te6+
n = 864
Frequency
(ae)
(af)
(ag)
Bond valence (v.u.)

Figure S3 Bond-length distributions for all configurations of the post-transition-metal ions bonded to O^{2-}: (*a*) $^{[4]}Al^{3+}$, (*b*) $^{[5]}Al^{3+}$, (*c*) $^{[6]}Al^{3+}$, (*d*) $^{[4]}Ga^{3+}$, (*e*) $^{[5]}Ga^{3+}$, (*f*) $^{[6]}Ga^{3+}$, (*g*) $^{[6]}In^{3+}$, (*h*) $^{[7]}In^{3+}$, (*i*) $^{[8]}In^{3+}$, (*j*) $^{[3]}Sn^{2+}$, (*k*) $^{[4]}Sn^{2+}$, (*l*) $^{[5]}Sn^{2+}$, (*m*) $^{[6]}Sn^{2+}$, (*n*) $^{[7]}Sn^{2+}$, (*o*) $^{[8]}Sn^{2+}$, (*p*) $^{[9]}Sn^{2+}$, (*q*) $^{[4]}Sn^{4+}$, (*r*) $^{[6]}Sn^{4+}$, (*s*) $^{[7]}Sn^{4+}$, (*t*) $^{[3]}Tl^{+}$, (*u*) $^{[4]}Tl^{+}$, (*v*) $^{[5]}Tl^{+}$, (*w*) $^{[6]}Tl^{+}$, (*x*) $^{[7]}Tl^{+}$, (*y*) $^{[8]}Tl^{+}$, (*z*) $^{[9]}Tl^{+}$, (*aa*) $^{[10]}Tl^{+}$, (*ab*) $^{[11]}Tl^{+}$, (*ac*) $^{[12]}Tl^{+}$, (*ad*) $^{[6]}Tl^{3+}$, (*ae*) $^{[7]}Tl^{3+}$, (*af*) $^{[8]}Tl^{3+}$, (*ag*) $^{[3]}Pb^{2+}$, (*ah*) $^{[4]}Pb^{2+}$, (*ai*) $^{[5]}Pb^{2+}$, (*aj*) $^{[6]}Pb^{2+}$, (*ak*) $^{[7]}Pb^{2+}$, (*al*) $^{[8]}Pb^{2+}$, (*am*) $^{[9]}Pb^{2+}$, (*an*) $^{[10]}Pb^{2+}$, (*ao*) $^{[11]}Pb^{2+}$, (*ap*) $^{[12]}Pb^{2+}$, (*aq*) $^{[4]}Pb^{4+}$, (*ar*) $^{[5]}Pb^{4+}$, (*as*) $^{[6]}Pb^{4+}$, (*at*) $^{[3]}Bi^{3+}$, (*au*) $^{[4]}Bi^{3+}$, (*av*) $^{[5]}Bi^{3+}$, (*aw*) $^{[6]}Bi^{3+}$, (*ax*) $^{[7]}Bi^{3+}$, (*ay*) $^{[8]}Bi^{3+}$, (*az*) $^{[9]}Bi^{3+}$, (*ba*) $^{[10]}Bi^{3+}$, (*bb*) $^{[12]}Bi^{3+}$, (*bc*) $^{[4]}Bi^{5+}$, (*bd*) $^{[6]}Bi^{5+}$.

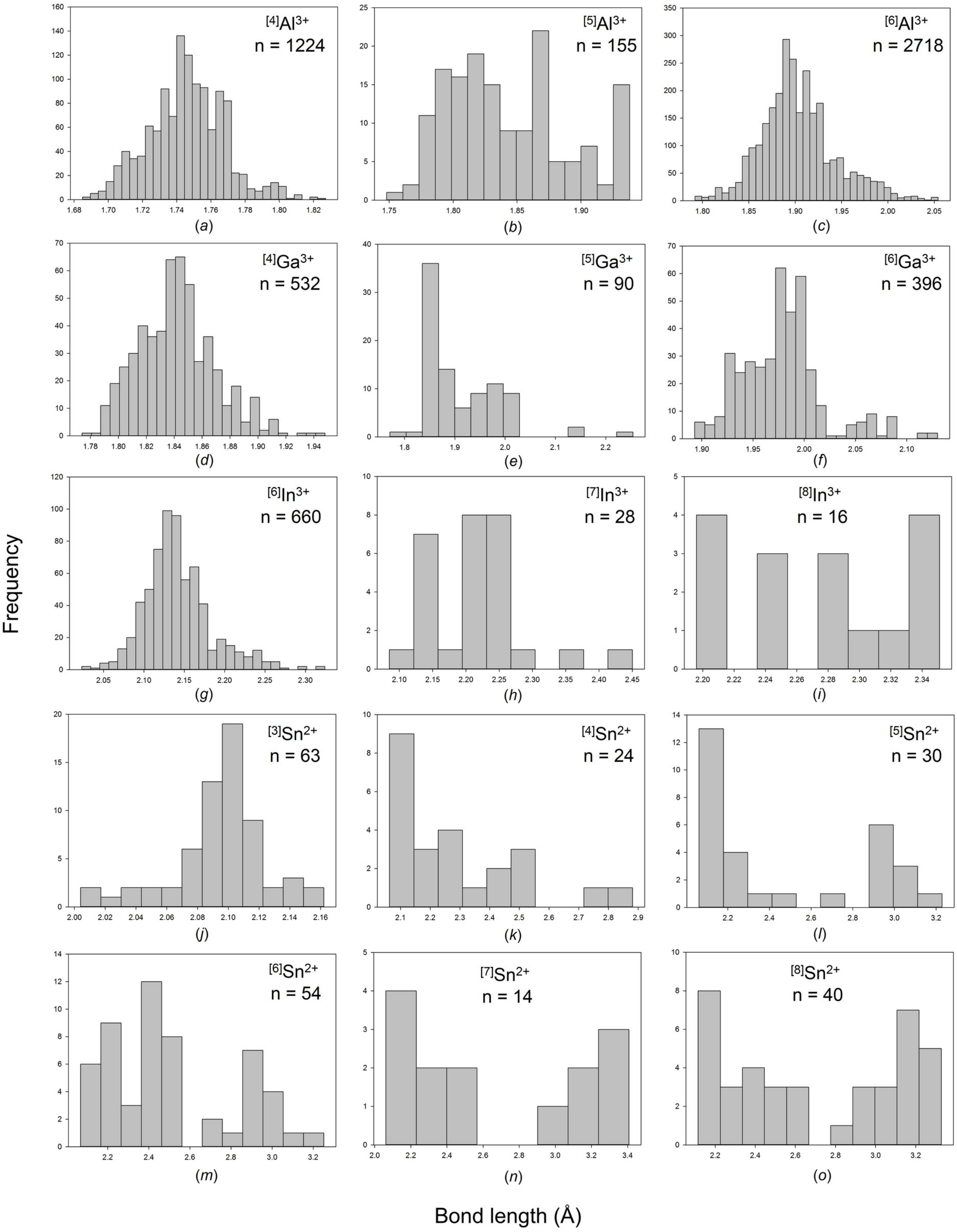

$^{[4]}Al^{3+}$ n = 1224
(a)
$^{[5]}Al^{3+}$ n = 155
(b)
$^{[6]}Al^{3+}$ n = 2718
(c)
$^{[4]}Ga^{3+}$ n = 532
(d)
$^{[5]}Ga^{3+}$ n = 90
(e)
$^{[6]}Ga^{3+}$ n = 396
(f)
$^{[6]}In^{3+}$ n = 660
(g)
$^{[7]}In^{3+}$ n = 28
(h)
$^{[8]}In^{3+}$ n = 16
(i)
$^{[3]}Sn^{2+}$ n = 63
(j)
$^{[4]}Sn^{2+}$ n = 24
(k)
$^{[5]}Sn^{2+}$ n = 30
(l)
$^{[6]}Sn^{2+}$ n = 54
(m)
$^{[7]}Sn^{2+}$ n = 14
(n)
$^{[8]}Sn^{2+}$ n = 40
(o)
Frequency
Bond length (Å)

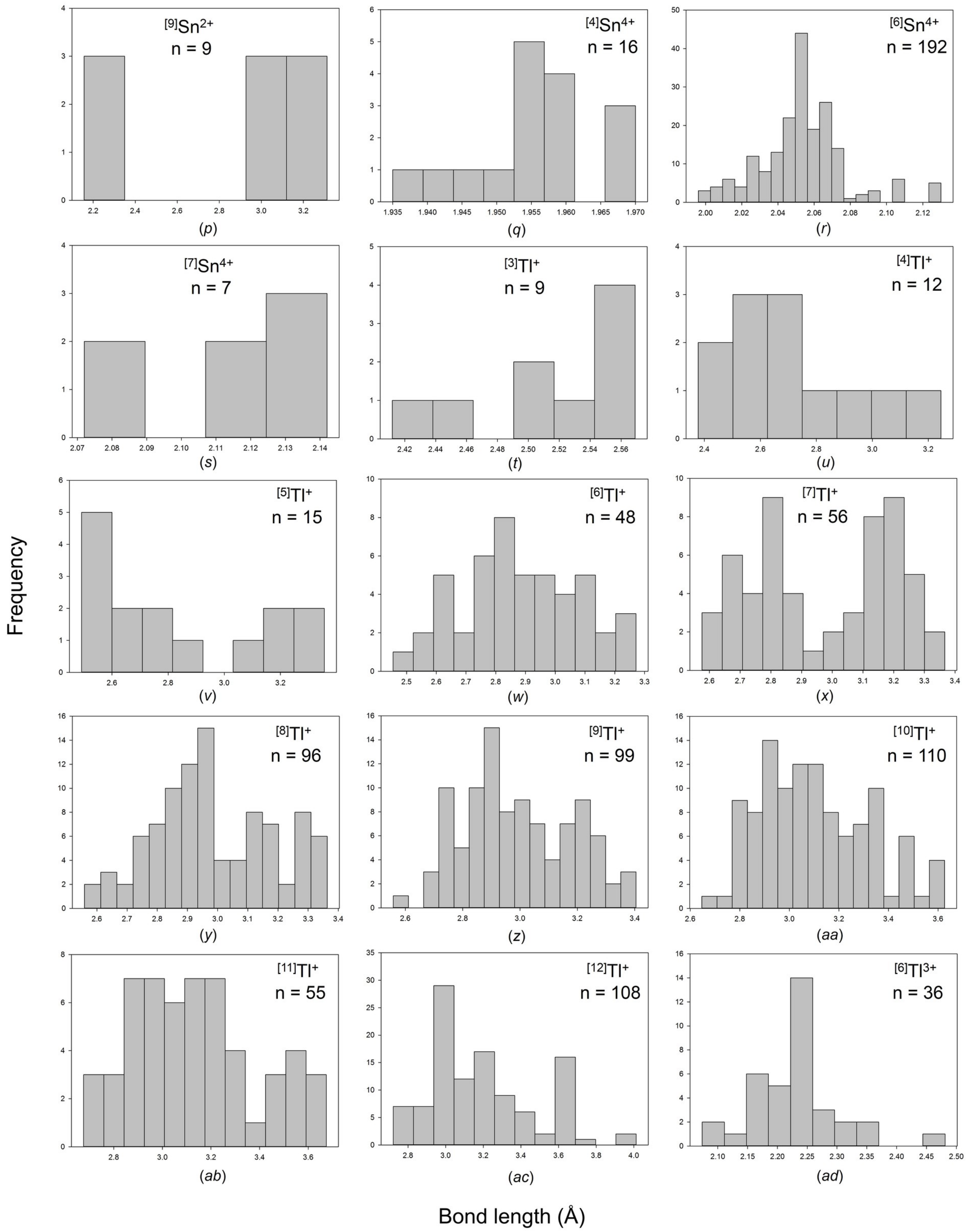
[9]Sn2+
n = 9
(p)
[4]Sn4+
n = 16
(q)
[6]Sn4+
n = 192
(r)
[7]Sn4+
n = 7
(s)
[3]Tl+
n = 9
(t)
[4]Tl+
n = 12
(u)
[5]Tl+
n = 15
(v)
[6]Tl+
n = 48
(w)
[7]Tl+
n = 56
(x)
[8]Tl+
n = 96
(y)
[9]Tl+
n = 99
(z)
[10]Tl+
n = 110
(aa)
[11]Tl+
n = 55
(ab)
[12]Tl+
n = 108
(ac)
[6]Tl3+
n = 36
(ad)
Frequency
Bond length (Å)

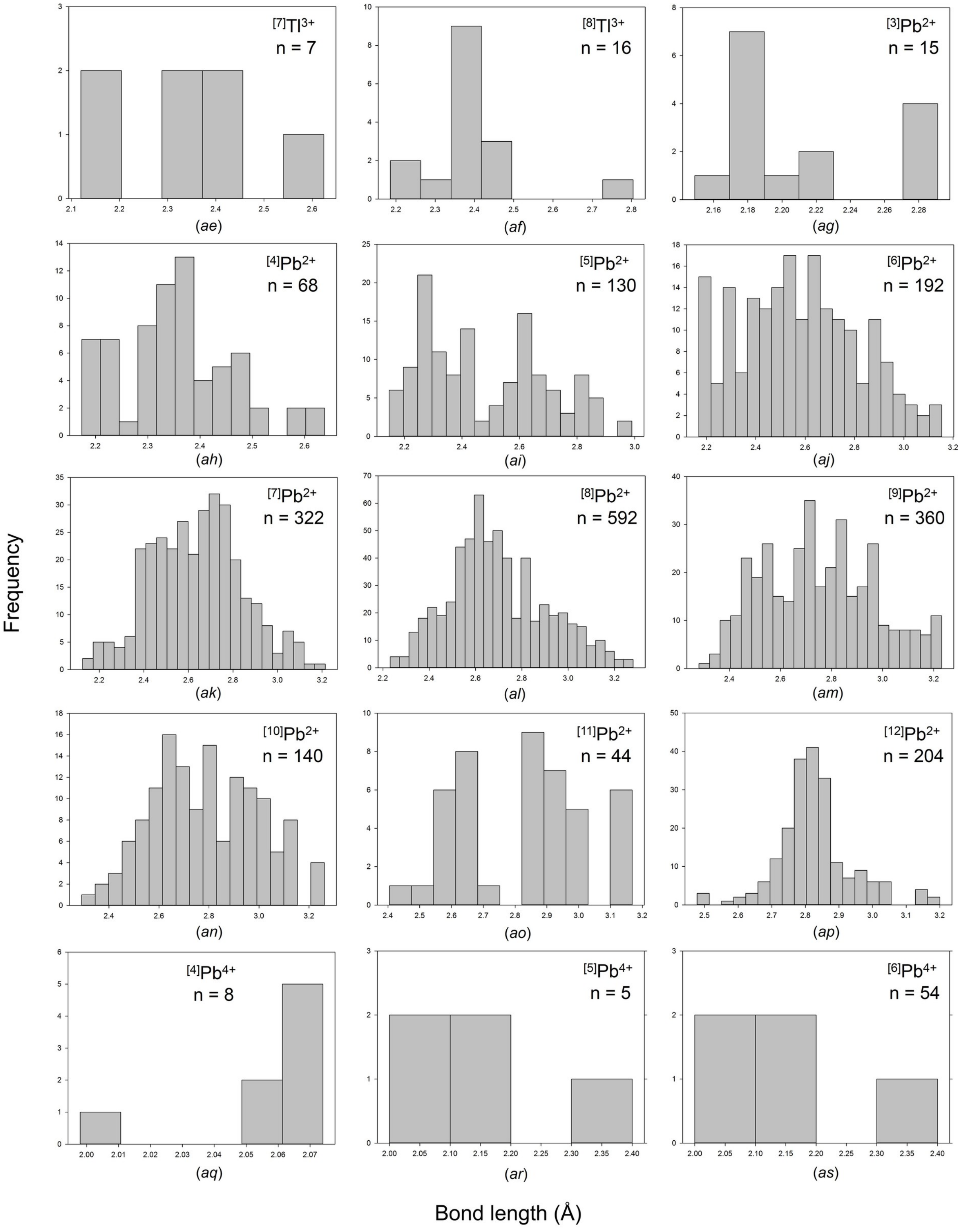

[7]Tl3+ n = 7
(ae)
[8]Tl3+ n = 16
(af)
[3]Pb2+ n = 15
(ag)
[4]Pb2+ n = 68
(ah)
[5]Pb2+ n = 130
(ai)
[6]Pb2+ n = 192
(aj)
[7]Pb2+ n = 322
(ak)
[8]Pb2+ n = 592
(al)
[9]Pb2+ n = 360
(am)
[10]Pb2+ n = 140
(an)
[11]Pb2+ n = 44
(ao)
[12]Pb2+ n = 204
(ap)
[4]Pb4+ n = 8
(aq)
[5]Pb4+ n = 5
(ar)
[6]Pb4+ n = 54
(as)
Frequency
Bond length (Å)

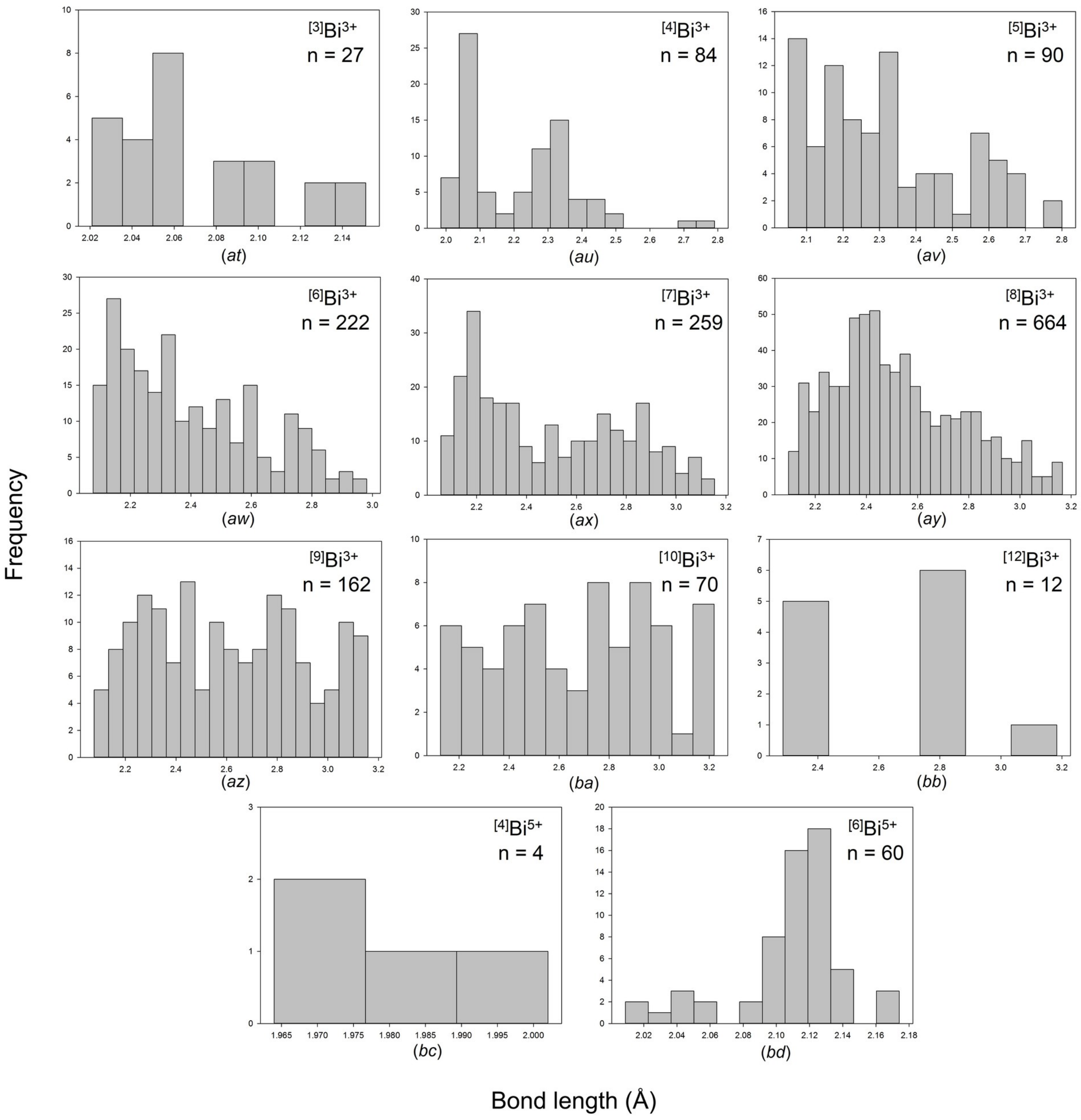
[3]Bi3+
n = 27
(at)
[4]Bi3+
n = 84
(au)
[5]Bi3+
n = 90
(av)
[6]Bi3+
n = 222
(aw)
[7]Bi3+
n = 259
(ax)
[8]Bi3+
n = 664
(ay)
[9]Bi3+
n = 162
(az)
[10]Bi3+
n = 70
(ba)
[12]Bi3+
n = 12
(bb)
[4]Bi5+
n = 4
(bc)
[6]Bi5+
n = 60
(bd)
Frequency
Bond length (Å)

Figure S4 Bond-valence distributions for all configurations of the post-transition-metal ions bonded to O^{2-}: (*a*) $^{[4]}Al^{3+}$, (*b*) $^{[5]}Al^{3+}$, (*c*) $^{[6]}Al^{3+}$, (*d*) $^{[4]}Ga^{3+}$, (*e*) $^{[5]}Ga^{3+}$, (*f*) $^{[6]}Ga^{3+}$, (*g*) $^{[6]}In^{3+}$, (*h*) $^{[7]}In^{3+}$, (*i*) $^{[8]}In^{3+}$, (*j*) $^{[3]}Sn^{2+}$, (*k*) $^{[4]}Sn^{2+}$, (*l*) $^{[5]}Sn^{2+}$, (*m*) $^{[6]}Sn^{2+}$, (*n*) $^{[7]}Sn^{2+}$, (*o*) $^{[8]}Sn^{2+}$, (*p*) $^{[9]}Sn^{2+}$, (*q*) $^{[4]}Sn^{4+}$, (*r*) $^{[6]}Sn^{4+}$, (*s*) $^{[7]}Sn^{4+}$, (*t*) $^{[3]}Tl^{+}$, (*u*) $^{[4]}Tl^{+}$, (*v*) $^{[5]}Tl^{+}$, (*w*) $^{[6]}Tl^{+}$, (*x*) $^{[7]}Tl^{+}$, (*y*) $^{[8]}Tl^{+}$, (*z*) $^{[9]}Tl^{+}$, (*aa*) $^{[10]}Tl^{+}$, (*ab*) $^{[11]}Tl^{+}$, (*ac*) $^{[12]}Tl^{+}$, (*ad*) $^{[6]}Tl^{3+}$, (*ae*) $^{[7]}Tl^{3+}$, (*af*) $^{[8]}Tl^{3+}$, (*ag*) $^{[3]}Pb^{2+}$, (*ah*) $^{[4]}Pb^{2+}$, (*ai*) $^{[5]}Pb^{2+}$, (*aj*) $^{[6]}Pb^{2+}$, (*ak*) $^{[7]}Pb^{2+}$, (*al*) $^{[8]}Pb^{2+}$, (*am*) $^{[9]}Pb^{2+}$, (*an*) $^{[10]}Pb^{2+}$, (*ao*) $^{[11]}Pb^{2+}$, (*ap*) $^{[12]}Pb^{2+}$, (*aq*) $^{[4]}Pb^{4+}$, (*ar*) $^{[5]}Pb^{4+}$, (*as*) $^{[6]}Pb^{4+}$, (*at*) $^{[3]}Bi^{3+}$, (*au*) $^{[4]}Bi^{3+}$, (*av*) $^{[5]}Bi^{3+}$, (*aw*) $^{[6]}Bi^{3+}$, (*ax*) $^{[7]}Bi^{3+}$, (*ay*) $^{[8]}Bi^{3+}$, (*az*) $^{[9]}Bi^{3+}$, (*ba*) $^{[10]}Bi^{3+}$, (*bb*) $^{[12]}Bi^{3+}$, (*bc*) $^{[4]}Bi^{5+}$, (*bd*) $^{[6]}Bi^{5+}$.

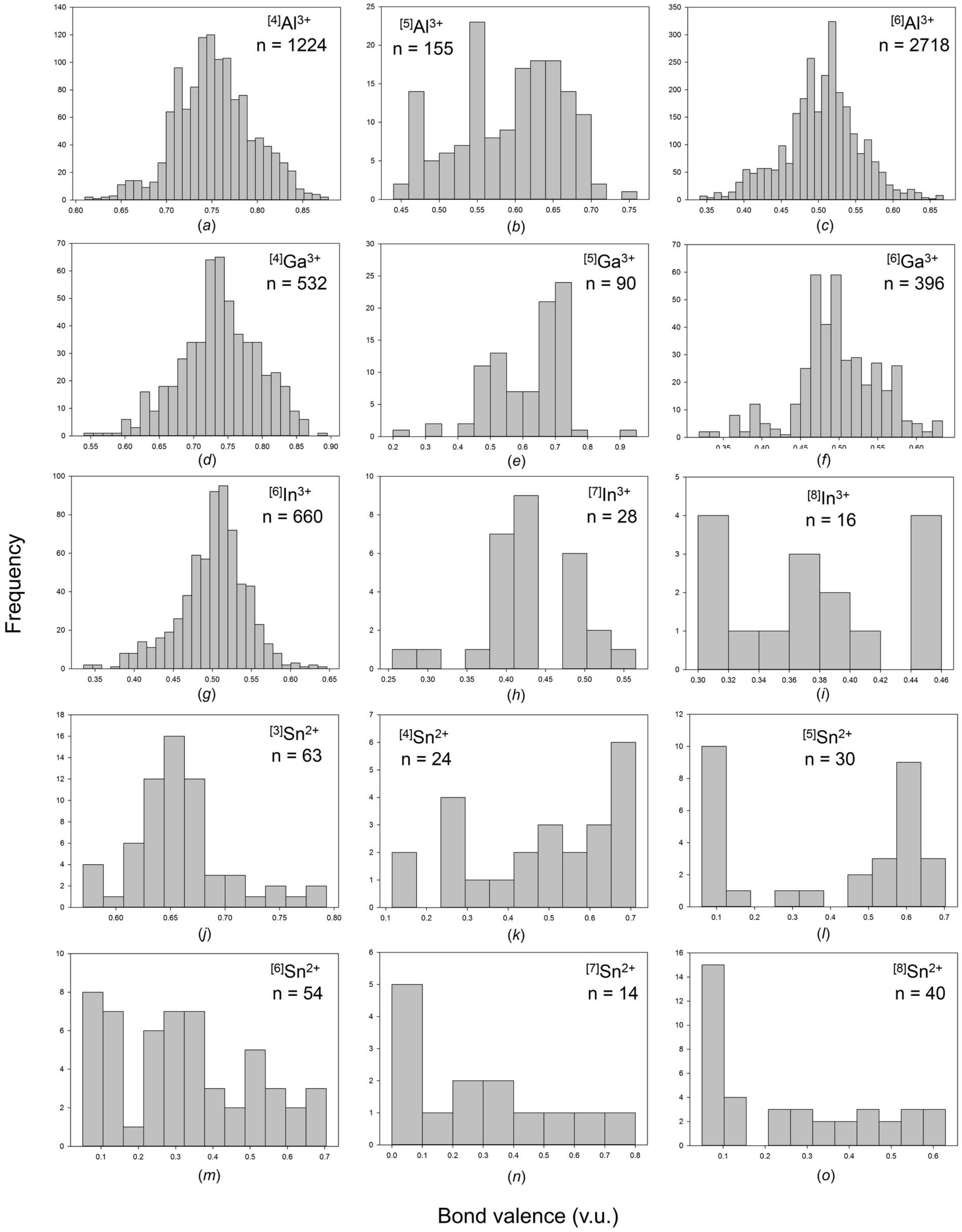
[4]Al3+
n = 1224
(a)
[5]Al3+
n = 155
(b)
[6]Al3+
n = 2718
(c)
[4]Ga3+
n = 532
(d)
[5]Ga3+
n = 90
(e)
[6]Ga3+
n = 396
(f)
[6]In3+
n = 660
(g)
[7]In3+
n = 28
(h)
[8]In3+
n = 16
(i)
[3]Sn2+
n = 63
(j)
[4]Sn2+
n = 24
(k)
[5]Sn2+
n = 30
(l)
[6]Sn2+
n = 54
(m)
[7]Sn2+
n = 14
(n)
[8]Sn2+
n = 40
(o)
Frequency
Bond valence (v.u.)

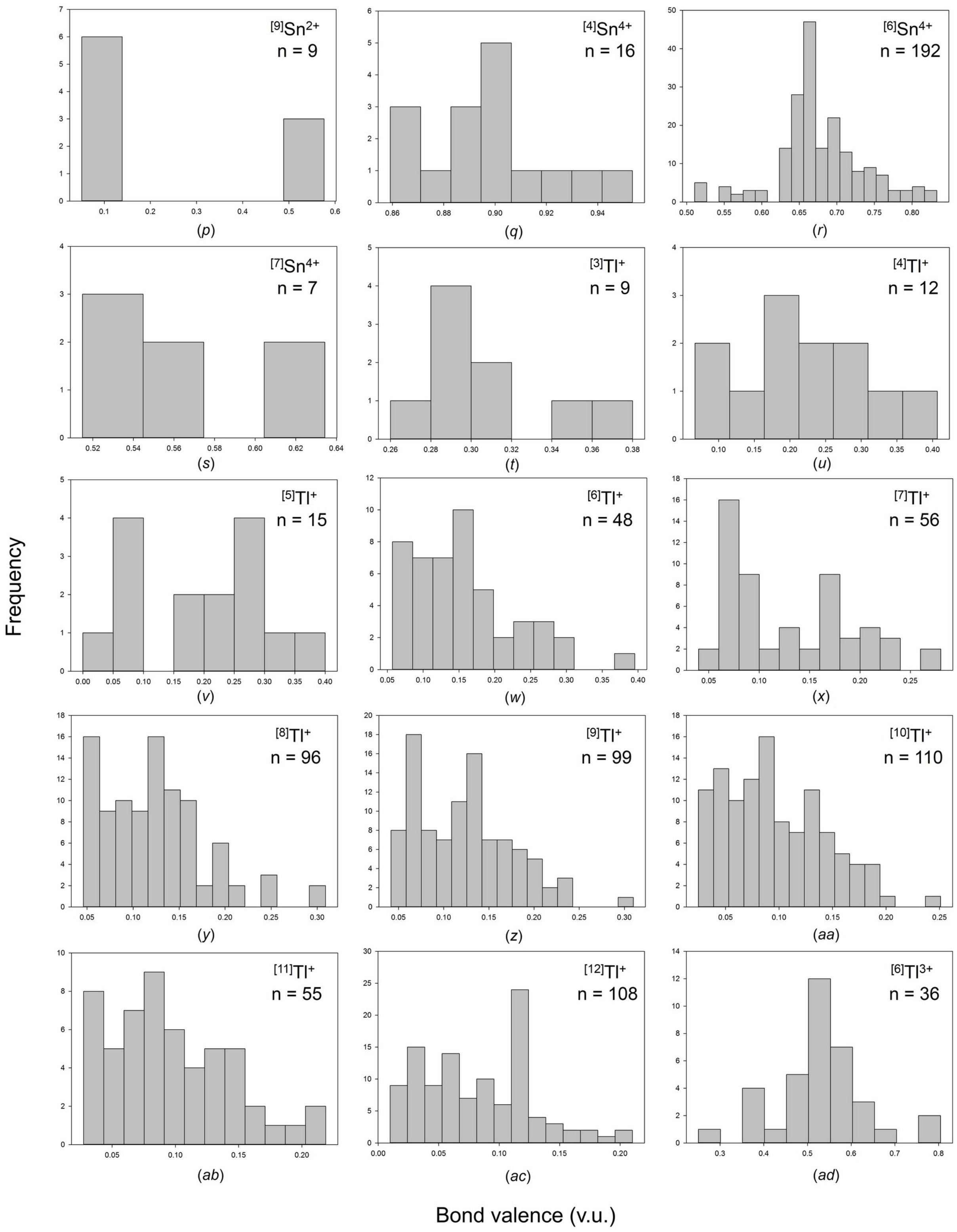
Frequency
[9]Sn2+ n = 9
(p)
[4]Sn4+ n = 16
(q)
[6]Sn4+ n = 192
(r)
[7]Sn4+ n = 7
(s)
[3]Tl+ n = 9
(t)
[4]Tl+ n = 12
(u)
[5]Tl+ n = 15
(v)
[6]Tl+ n = 48
(w)
[7]Tl+ n = 56
(x)
[8]Tl+ n = 96
(y)
[9]Tl+ n = 99
(z)
[10]Tl+ n = 110
(aa)
[11]Tl+ n = 55
(ab)
[12]Tl+ n = 108
(ac)
[6]Tl3+ n = 36
(ad)
Bond valence (v.u.)

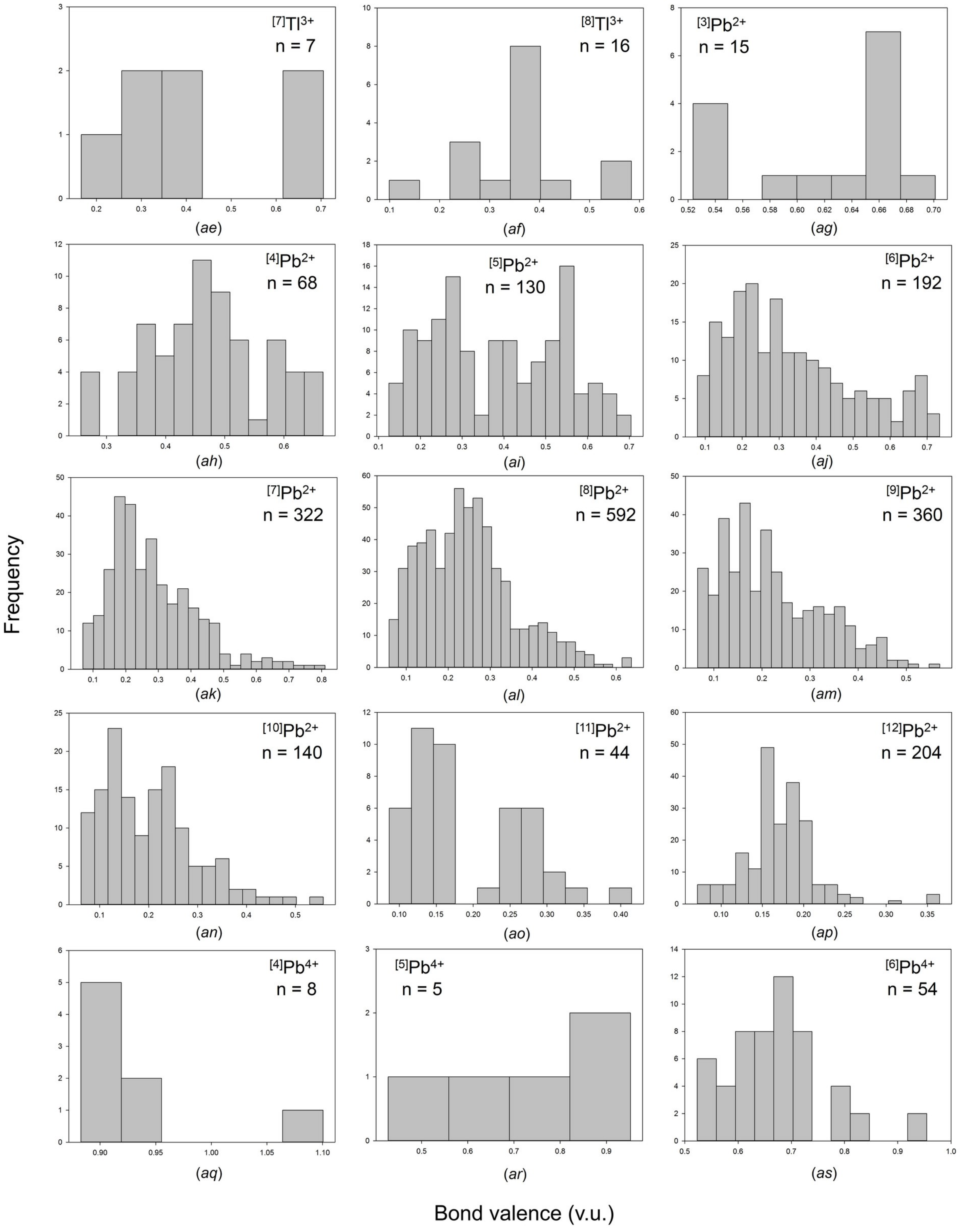
[7]Tl3+
n = 7
(ae)
[8]Tl3+
n = 16
(af)
[3]Pb2+
n = 15
(ag)
[4]Pb2+
n = 68
(ah)
[5]Pb2+
n = 130
(ai)
[6]Pb2+
n = 192
(aj)
[7]Pb2+
n = 322
(ak)
[8]Pb2+
n = 592
(al)
[9]Pb2+
n = 360
(am)
[10]Pb2+
n = 140
(an)
[11]Pb2+
n = 44
(ao)
[12]Pb2+
n = 204
(ap)
[4]Pb4+
n = 8
(aq)
[5]Pb4+
n = 5
(ar)
[6]Pb4+
n = 54
(as)
Frequency
Bond valence (v.u.)

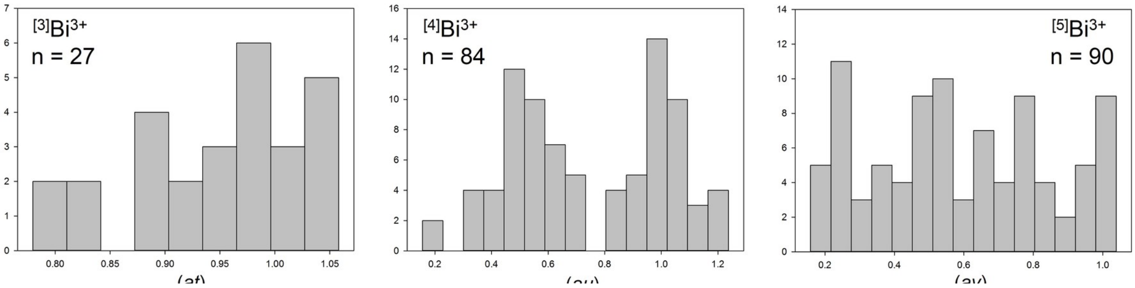
[3]Bi3+
n = 27
[4]Bi3+
n = 84
[5]Bi3+
n = 90

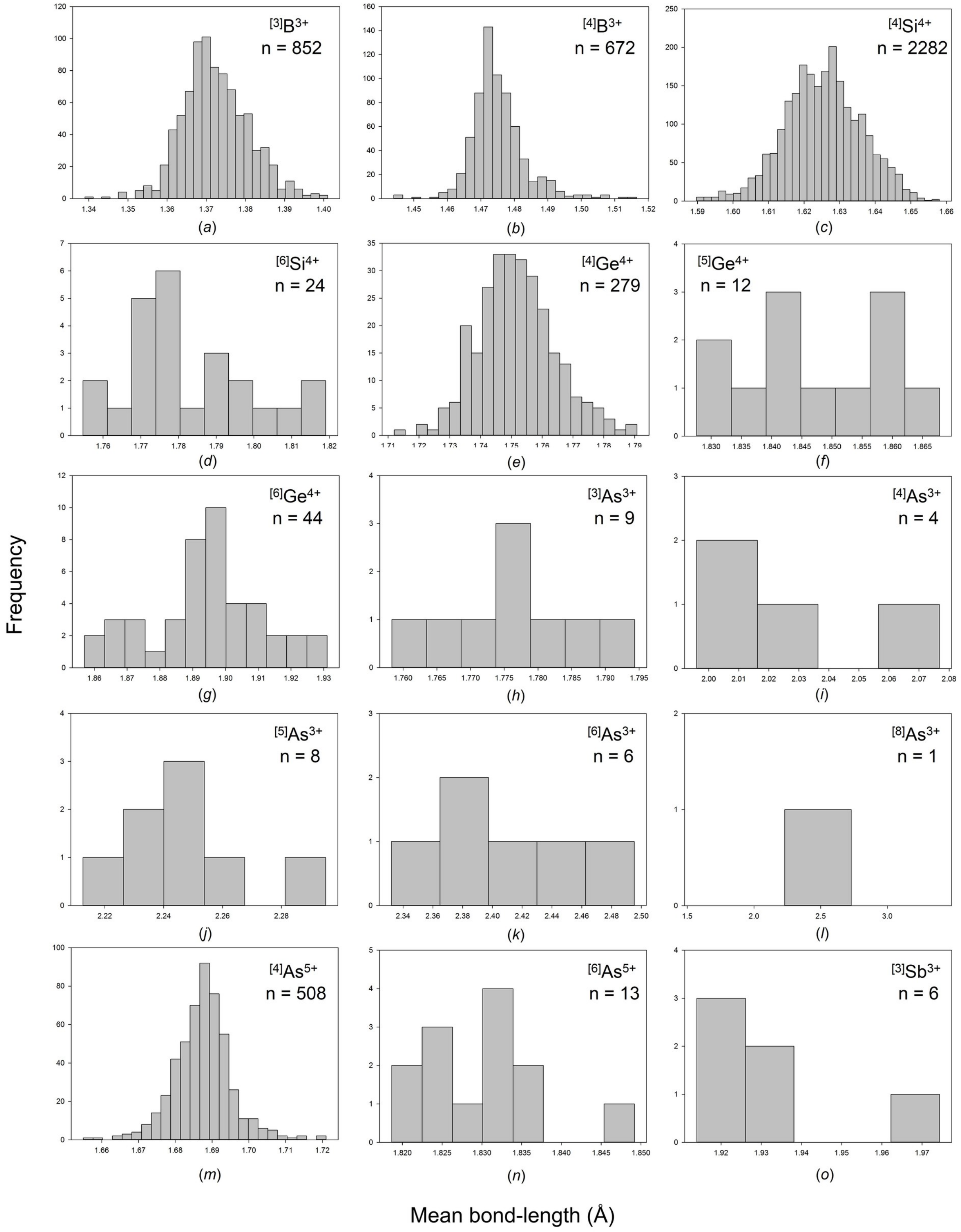
[3]B3+
n = 852
(a)
[4]B3+
n = 672
(b)
[4]Si4+
n = 2282
(c)
[6]Si4+
n = 24
(d)
[4]Ge4+
n = 279
(e)
[5]Ge4+
n = 12
(f)
[6]Ge4+
n = 44
(g)
[3]As3+
n = 9
(h)
[4]As3+
n = 4
(i)
[5]As3+
n = 8
(j)
[6]As3+
n = 6
(k)
[8]As3+
n = 1
(l)
[4]As5+
n = 508
(m)
[6]As5+
n = 13
(n)
[3]Sb3+
n = 6
(o)
Frequency
Mean bond-length (Å)

Figure S5 Mean-bond-length distributions for all configurations of the metalloid ions bonded to O^{2-} : (*a*) $^{[3]}B^{3+}$, (*b*) $^{[4]}B^{3+}$, (*c*) $^{[4]}Si^{4+}$, (*d*) $^{[6]}Si^{4+}$, (*e*) $^{[4]}Ge^{4+}$, (*f*) $^{[5]}Ge^{4+}$, (*g*) $^{[6]}Ge^{4+}$, (*h*) $^{[3]}As^{3+}$, (*i*) $^{[4]}As^{3+}$, (*j*) $^{[5]}As^{3+}$, (*k*) $^{[6]}As^{3+}$, (*l*) $^{[8]}As^{3+}$, (*m*) $^{[4]}As^{5+}$, (*n*) $^{[6]}As^{5+}$, (*o*) $^{[3]}Sb^{3+}$, (*p*) $^{[4]}Sb^{3+}$, (*q*) $^{[5]}Sb^{3+}$, (*r*) $^{[6]}Sb^{3+}$, (*s*) $^{[7]}Sb^{3+}$, (*t*) $^{[8]}Sb^{3+}$, (*u*) $^{[9]}Sb^{3+}$, (*v*) $^{[6]}Sb^{5+}$, (*w*) $^{[3]}Te^{4+}$, (*x*) $^{[4]}Te^{4+}$, (*y*) $^{[5]}Te^{4+}$, (*z*) $^{[6]}Te^{4+}$, (*aa*) $^{[7]}Te^{4+}$, (*ab*) $^{[8]}Te^{4+}$, (*ac*) $^{[9]}Te^{4+}$, (*ad*) $^{[10]}Te^{4+}$, (*ae*) $^{[11]}Te^{4+}$, (*af*) $^{[12]}Te^{4+}$, (*ag*) $^{[6]}Te^{6+}$.

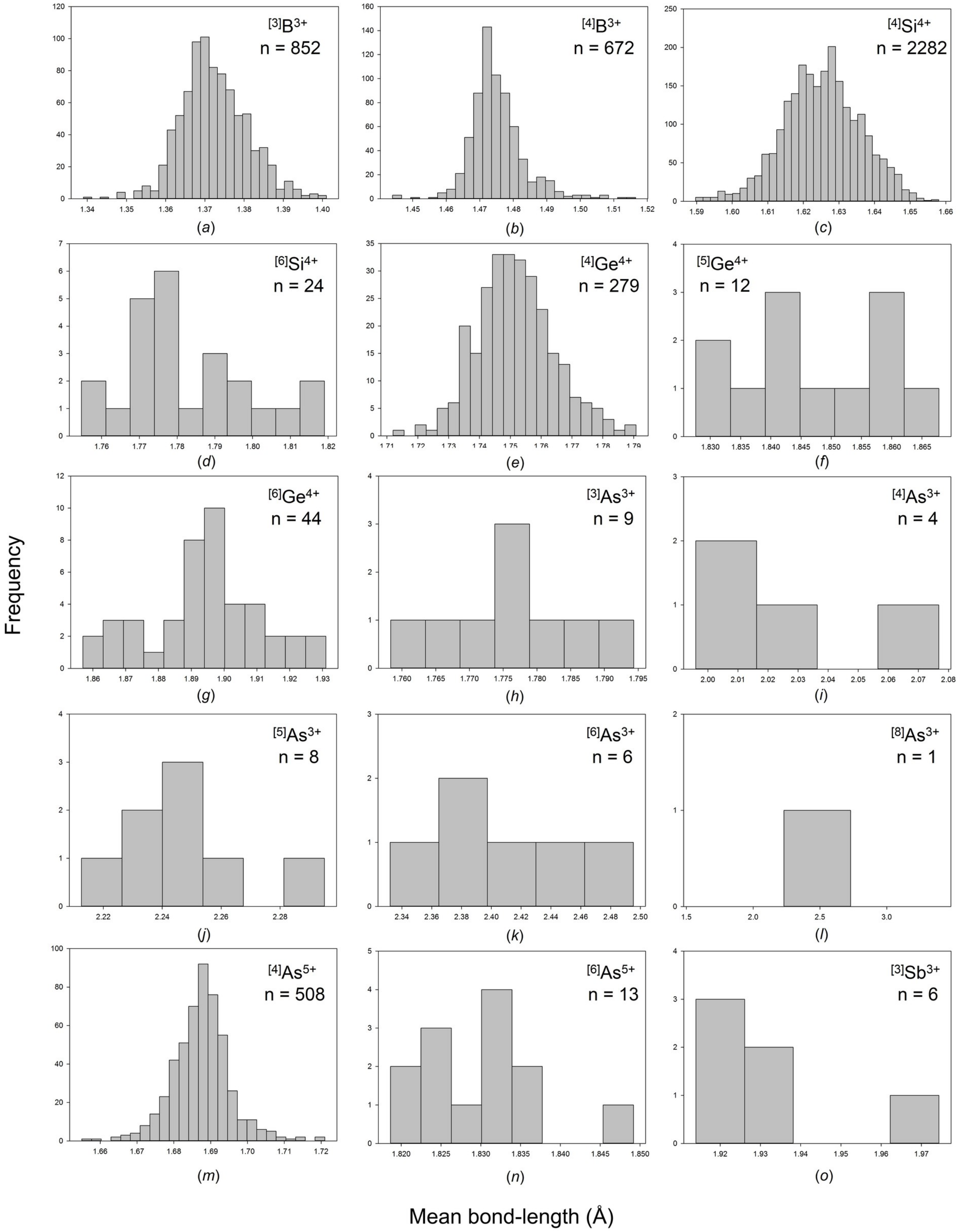
[3]B3+
n = 852
(a)
[4]B3+
n = 672
(b)
[4]Si4+
n = 2282
(c)
[6]Si4+
n = 24
(d)
[4]Ge4+
n = 279
(e)
[5]Ge4+
n = 12
(f)
[6]Ge4+
n = 44
(g)
[3]As3+
n = 9
(h)
[4]As3+
n = 4
(i)
[5]As3+
n = 8
(j)
[6]As3+
n = 6
(k)
[8]As3+
n = 1
(l)
[4]As5+
n = 508
(m)
[6]As5+
n = 13
(n)
[3]Sb3+
n = 6
(o)
Frequency
Mean bond-length (Å)

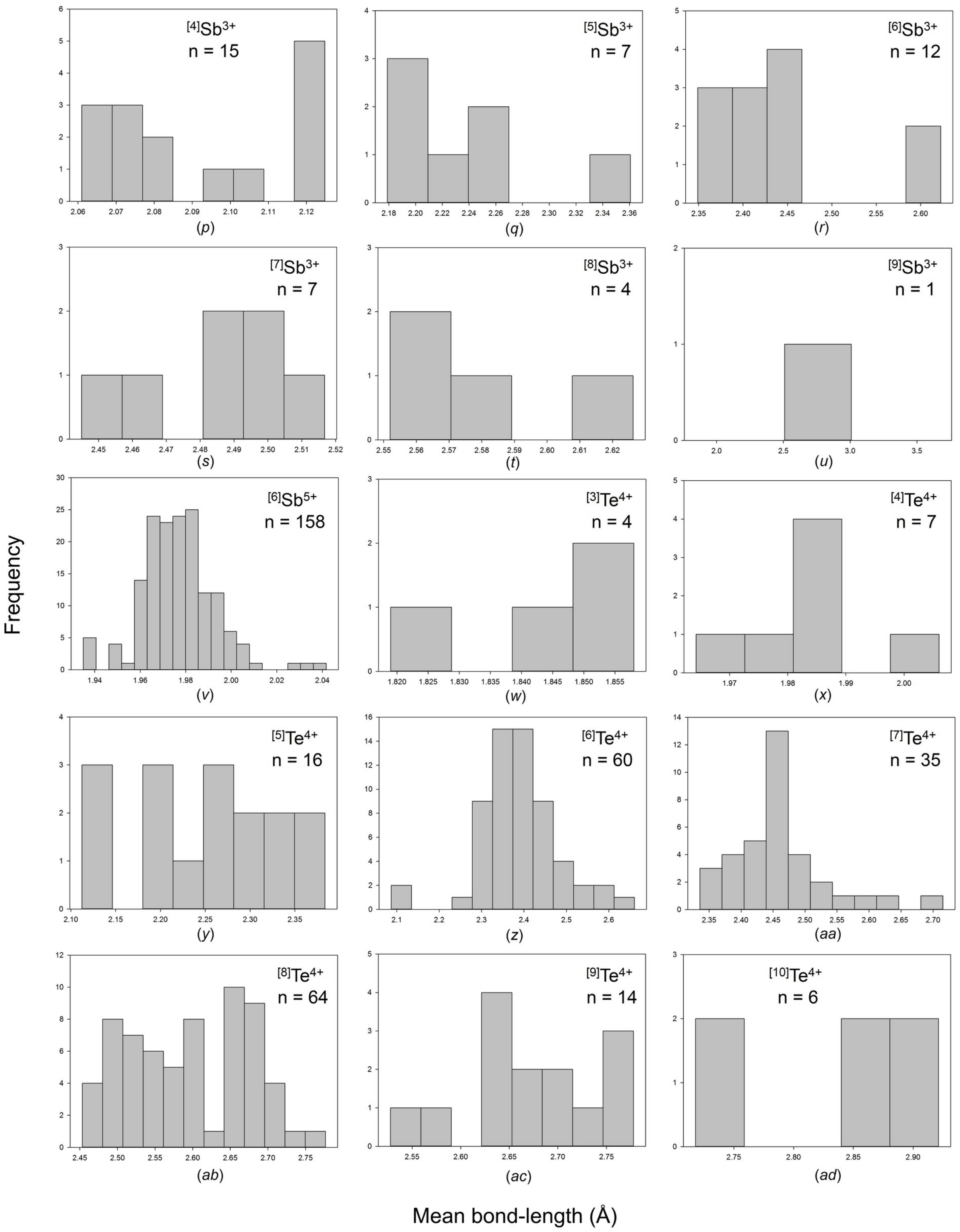
Frequency
[4]Sb3+ n = 15
(p)
[5]Sb3+ n = 7
(q)
[6]Sb3+ n = 12
(r)
[7]Sb3+ n = 7
(s)
[8]Sb3+ n = 4
(t)
[9]Sb3+ n = 1
(u)
[6]Sb5+ n = 158
(v)
[3]Te4+ n = 4
(w)
[4]Te4+ n = 7
(x)
[5]Te4+ n = 16
(y)
[6]Te4+ n = 60
(z)
[7]Te4+ n = 35
(aa)
[8]Te4+ n = 64
(ab)
[9]Te4+ n = 14
(ac)
[10]Te4+ n = 6
(ad)
Mean bond-length (Å)

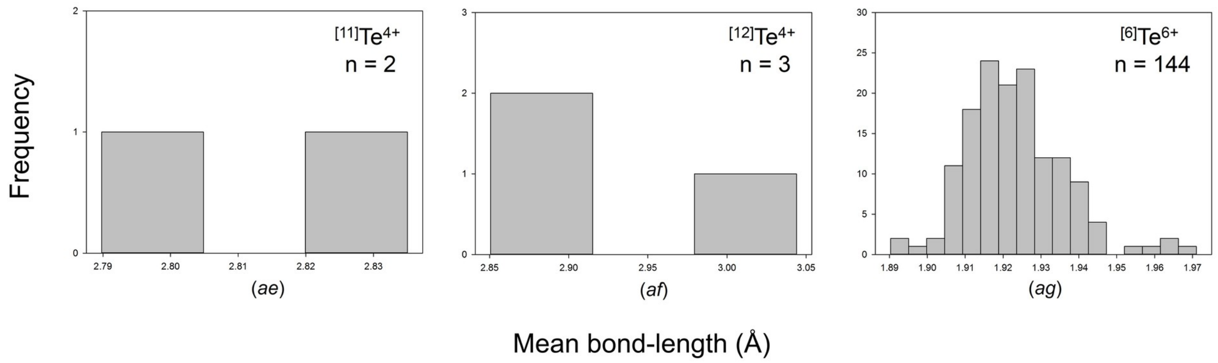
Frequency
$^{[11]}Te^{4+}$
n = 2
(ae)
$^{[12]}Te^{4+}$
n = 3
(af)
$^{[6]}Te^{6+}$
n = 144
(ag)
Mean bond-length (Å)

Figure S6 Mean-bond-length distributions for all configurations of the post-transition-metal ions bonded to O^{2-}: (*a*) $^{[4]}Al^{3+}$, (*b*) $^{[5]}Al^{3+}$, (*c*) $^{[6]}Al^{3+}$, (*d*) $^{[4]}Ga^{3+}$, (*e*) $^{[5]}Ga^{3+}$, (*f*) $^{[6]}Ga^{3+}$, (*g*) $^{[6]}In^{3+}$, (*h*) $^{[7]}In^{3+}$, (*i*) $^{[8]}In^{3+}$, (*j*) $^{[3]}Sn^{2+}$, (*k*) $^{[4]}Sn^{2+}$, (*l*) $^{[5]}Sn^{2+}$, (*m*) $^{[6]}Sn^{2+}$, (*n*) $^{[7]}Sn^{2+}$, (*o*) $^{[8]}Sn^{2+}$, (*p*) $^{[9]}Sn^{2+}$, (*q*) $^{[4]}Sn^{4+}$, (*r*) $^{[6]}Sn^{4+}$, (*s*) $^{[7]}Sn^{4+}$, (*t*) $^{[3]}Tl^{+}$, (*u*) $^{[4]}Tl^{+}$, (*v*) $^{[5]}Tl^{+}$, (*w*) $^{[6]}Tl^{+}$, (*x*) $^{[7]}Tl^{+}$, (*y*) $^{[8]}Tl^{+}$, (*z*) $^{[9]}Tl^{+}$, (*aa*) $^{[10]}Tl^{+}$, (*ab*) $^{[11]}Tl^{+}$, (*ac*) $^{[12]}Tl^{+}$, (*ad*) $^{[6]}Tl^{3+}$, (*ae*) $^{[7]}Tl^{3+}$, (*af*) $^{[8]}Tl^{3+}$, (*ag*) $^{[3]}Pb^{2+}$, (*ah*) $^{[4]}Pb^{2+}$, (*ai*) $^{[5]}Pb^{2+}$, (*aj*) $^{[6]}Pb^{2+}$, (*ak*) $^{[7]}Pb^{2+}$, (*al*) $^{[8]}Pb^{2+}$, (*am*) $^{[9]}Pb^{2+}$, (*an*) $^{[10]}Pb^{2+}$, (*ao*) $^{[11]}Pb^{2+}$, (*ap*) $^{[12]}Pb^{2+}$, (*aq*) $^{[4]}Pb^{4+}$, (*ar*) $^{[5]}Pb^{4+}$, (*as*) $^{[6]}Pb^{4+}$, (*at*) $^{[3]}Bi^{3+}$, (*au*) $^{[4]}Bi^{3+}$, (*av*) $^{[5]}Bi^{3+}$, (*aw*) $^{[6]}Bi^{3+}$, (*ax*) $^{[7]}Bi^{3+}$, (*ay*) $^{[8]}Bi^{3+}$, (*az*) $^{[9]}Bi^{3+}$, (*ba*) $^{[10]}Bi^{3+}$, (*bb*) $^{[12]}Bi^{3+}$, (*bc*) $^{[4]}Bi^{5+}$, (*bd*) $^{[6]}Bi^{5+}$.

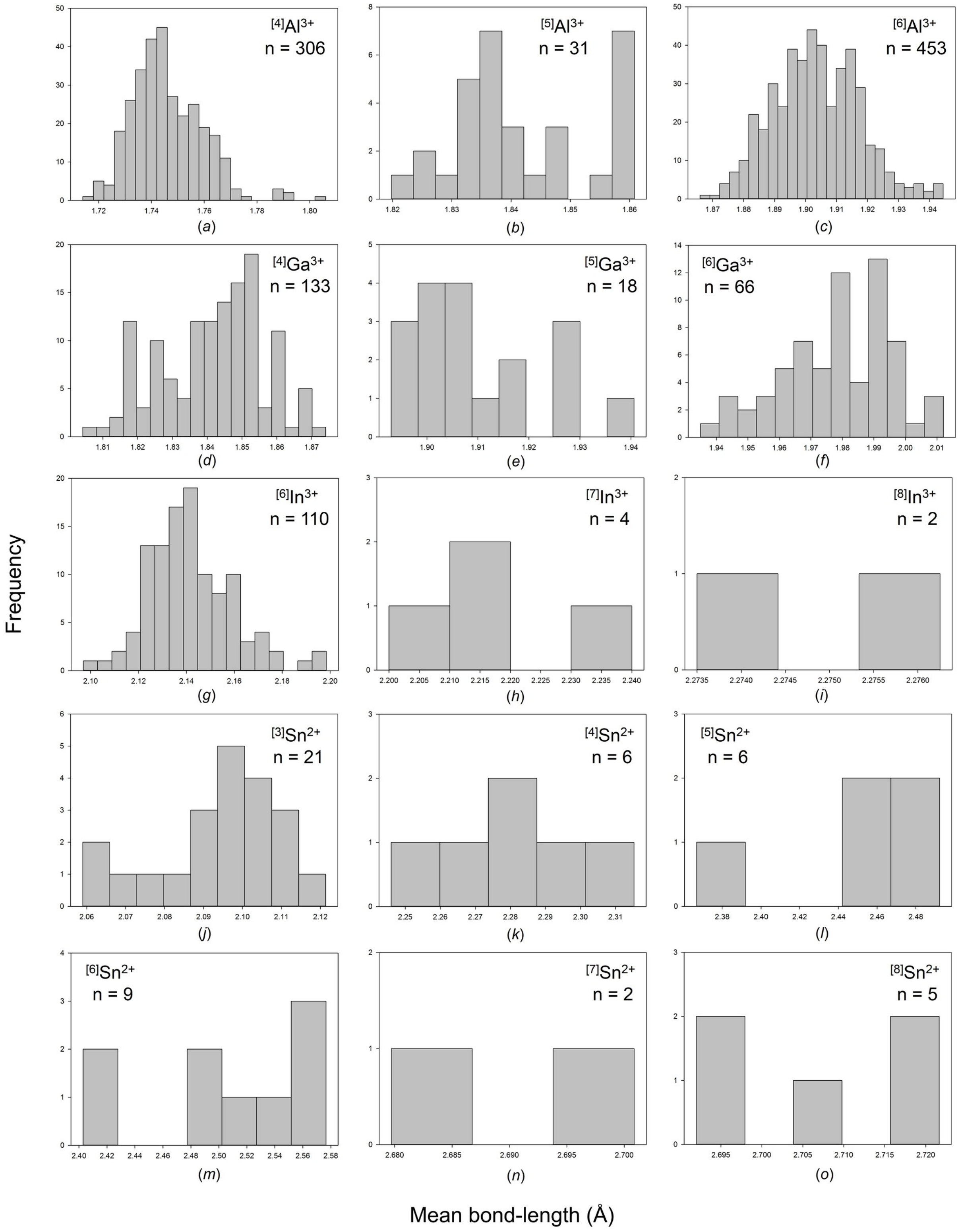
[4]Al3+
n = 306
[5]Al3+
n = 31
[6]Al3+
n = 453
[4]Ga3+
n = 133
[5]Ga3+
n = 18
[6]Ga3+
n = 66
[6]In3+
n = 110
[7]In3+
n = 4
[8]In3+
n = 2
[3]Sn2+
n = 21
[4]Sn2+
n = 6
[5]Sn2+
n = 6
[6]Sn2+
n = 9
[7]Sn2+
n = 2
[8]Sn2+
n = 5
(a)
(b)
(c)
(d)
(e)
(f)
(g)
(h)
(i)
(j)
(k)
(l)
(m)
(n)
(o)
Frequency
Mean bond-length (Å)

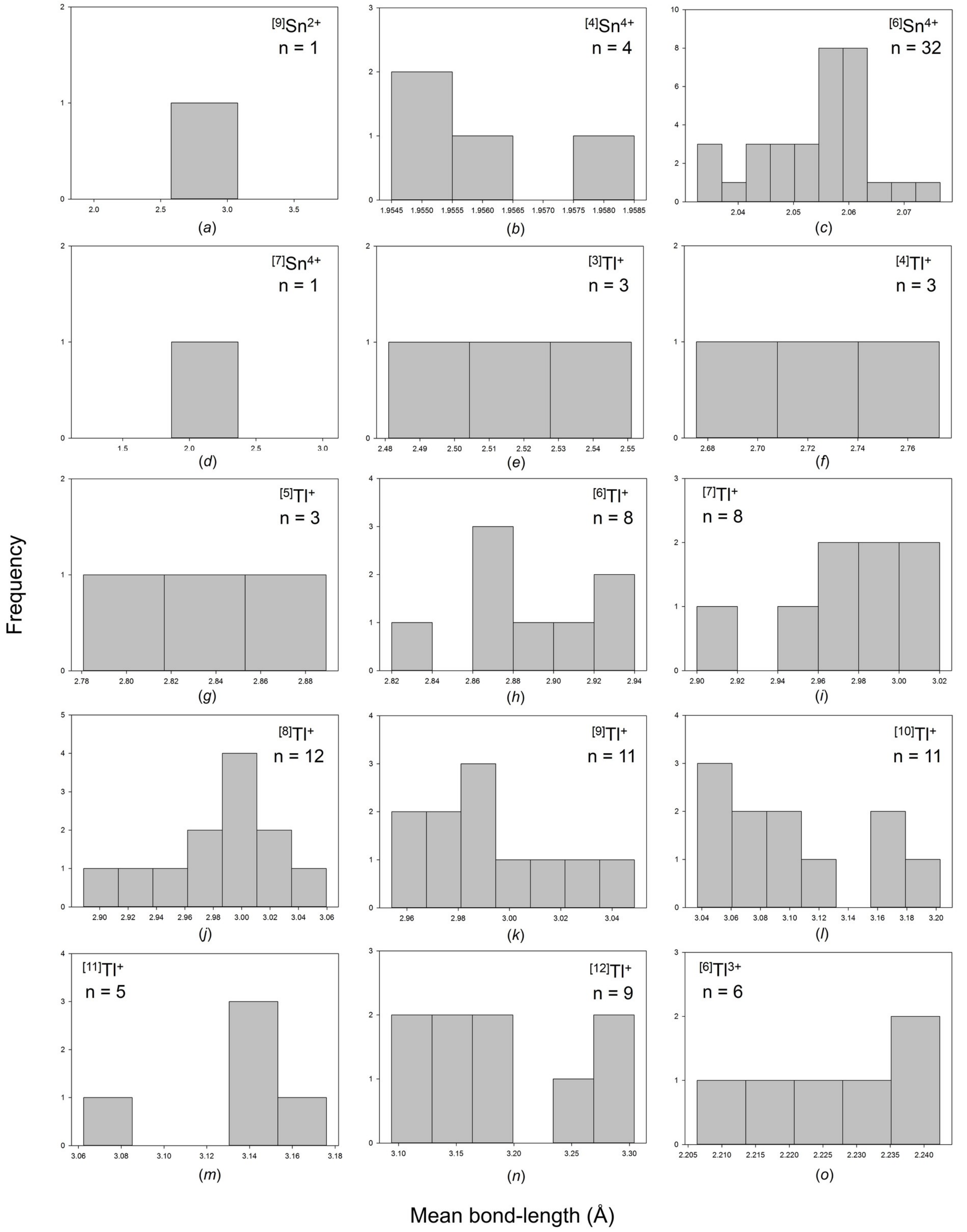
[9]Sn2+
n = 1
(a)
[4]Sn4+
n = 4
(b)
[6]Sn4+
n = 32
(c)
[7]Sn4+
n = 1
(d)
[3]Tl+
n = 3
(e)
[4]Tl+
n = 3
(f)
[5]Tl+
n = 3
(g)
[6]Tl+
n = 8
(h)
[7]Tl+
n = 8
(i)
[8]Tl+
n = 12
(j)
[9]Tl+
n = 11
(k)
[10]Tl+
n = 11
(l)
[11]Tl+
n = 5
(m)
[12]Tl+
n = 9
(n)
[6]Tl3+
n = 6
(o)
Frequency
Mean bond-length (Å)

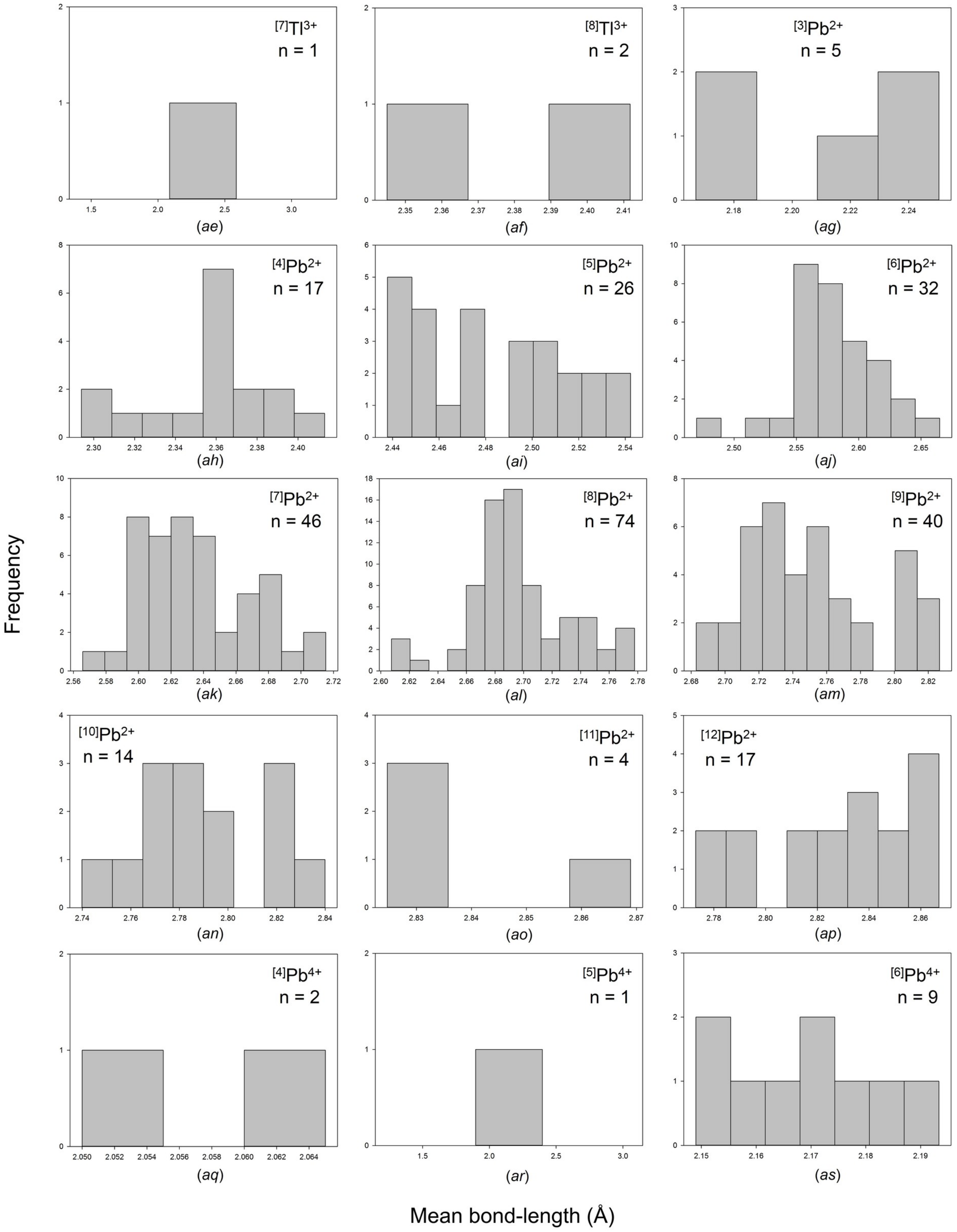
[7]Tl3+
n = 1
(ae)
[8]Tl3+
n = 2
(af)
[3]Pb2+
n = 5
(ag)
[4]Pb2+
n = 17
(ah)
[5]Pb2+
n = 26
(ai)
[6]Pb2+
n = 32
(aj)
[7]Pb2+
n = 46
(ak)
[8]Pb2+
n = 74
(al)
[9]Pb2+
n = 40
(am)
[10]Pb2+
n = 14
(an)
[11]Pb2+
n = 4
(ao)
[12]Pb2+
n = 17
(ap)
[4]Pb4+
n = 2
(aq)
[5]Pb4+
n = 1
(ar)
[6]Pb4+
n = 9
(as)
Frequency
Mean bond-length (Å)

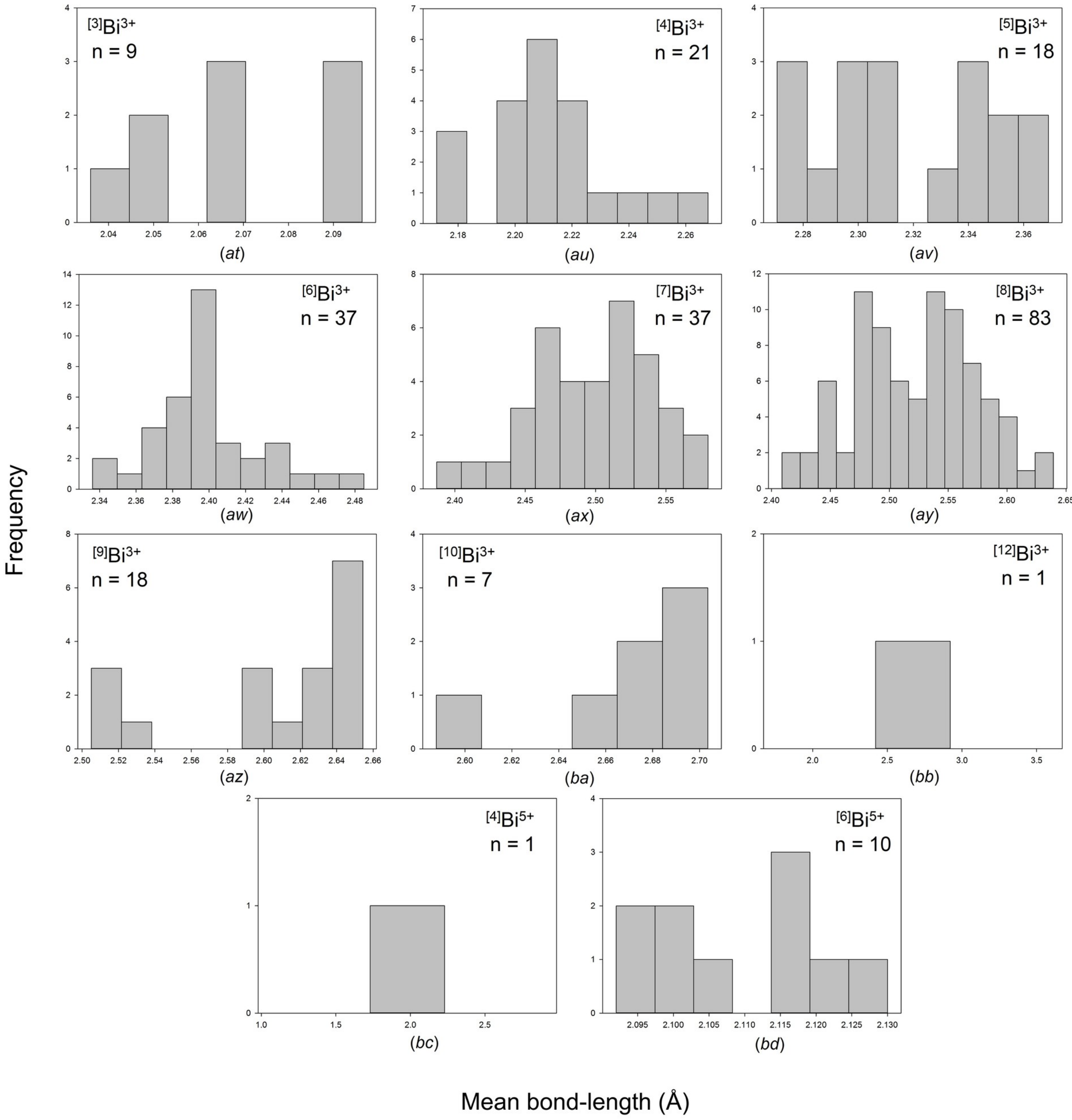

[3]Bi3+
n = 9
(at)
[4]Bi3+
n = 21
(au)
[5]Bi3+
n = 18
(av)
[6]Bi3+
n = 37
(aw)
[7]Bi3+
n = 37
(ax)
[8]Bi3+
n = 83
(ay)
[9]Bi3+
n = 18
(az)
[10]Bi3+
n = 7
(ba)
[12]Bi3+
n = 1
(bb)
[4]Bi5+
n = 1
(bc)
[6]Bi5+
n = 10
(bd)
Frequency
Mean bond-length (Å)

Figure S7 The effect of bond-length distortion on mean bond-length for all configurations of the metalloid ions bonded to O^{2-}: (*a*) $^{[3]}B^{3+}$, (*b*) $^{[4]}B^{3+}$, (*c*) $^{[4]}Si^{4+}$, (*d*) $^{[6]}Si^{4+}$, (*e*) $^{[4]}Ge^{4+}$, (*f*) $^{[5]}Ge^{4+}$, (*g*) $^{[6]}Ge^{4+}$, (*h*) $^{[3]}As^{3+}$, (*i*) $^{[4]}As^{3+}$, (*j*) $^{[5]}As^{3+}$, (*k*) $^{[6]}As^{3+}$, (*l*) $^{[8]}As^{3+}$, (*m*) $^{[4]}As^{5+}$, (*n*) $^{[6]}As^{5+}$, (*o*) $^{[3]}Sb^{3+}$, (*p*) $^{[4]}Sb^{3+}$, (*q*) $^{[5]}Sb^{3+}$, (*r*) $^{[6]}Sb^{3+}$, (*s*) $^{[7]}Sb^{3+}$, (*t*) $^{[8]}Sb^{3+}$, (*u*) $^{[9]}Sb^{3+}$, (*v*) $^{[6]}Sb^{5+}$, (*w*) $^{[3]}Te^{4+}$, (*x*) $^{[4]}Te^{4+}$, (*y*) $^{[5]}Te^{4+}$, (*z*) $^{[6]}Te^{4+}$, (*aa*) $^{[7]}Te^{4+}$, (*ab*) $^{[8]}Te^{4+}$, (*ac*) $^{[9]}Te^{4+}$, (*ad*) $^{[10]}Te^{4+}$, (*ae*) $^{[11]}Te^{4+}$, (*af*) $^{[12]}Te^{4+}$, (*ag*) $^{[6]}Te^{6+}$.

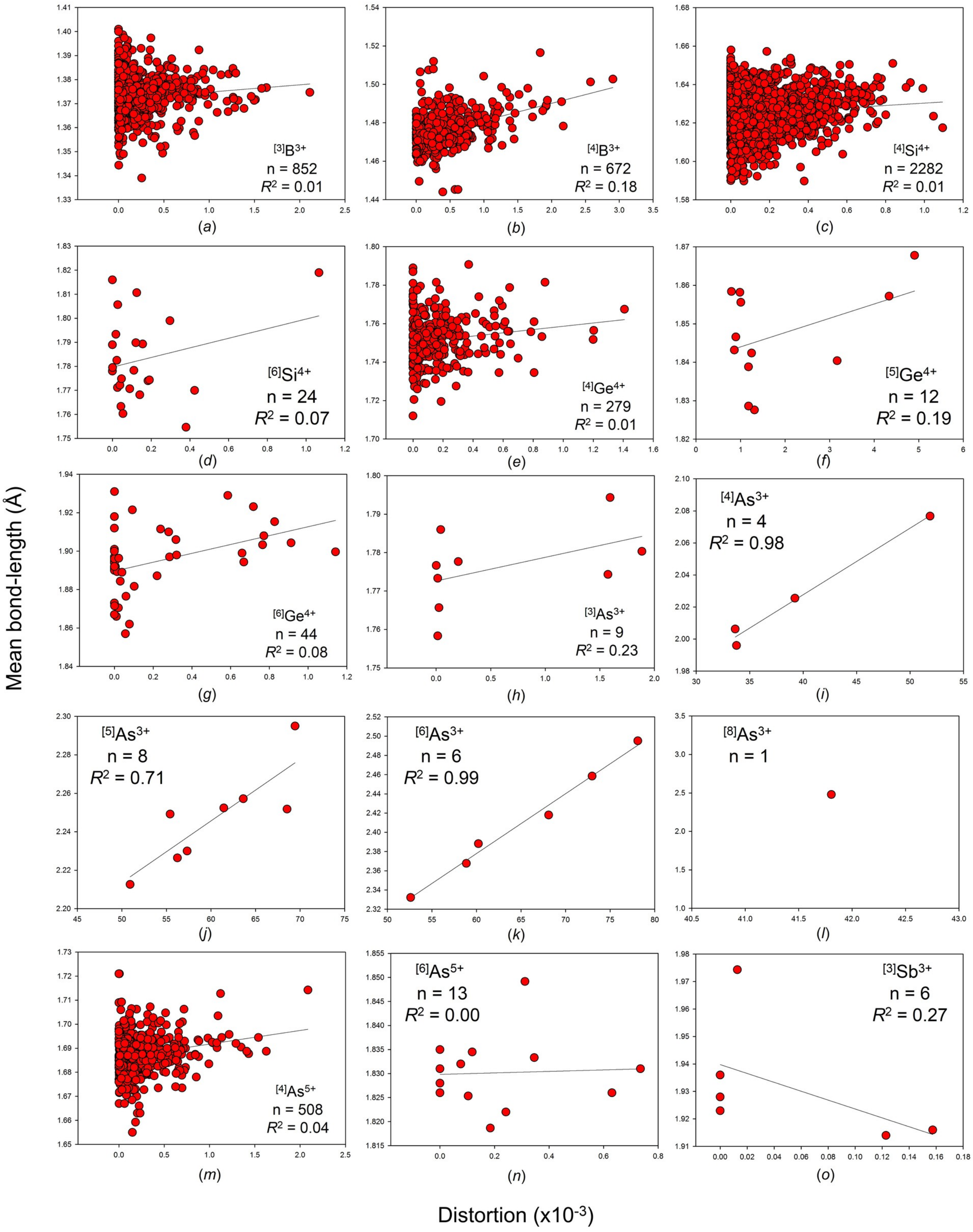
Mean bond-length (Å)
Distortion (x10-3)
[3]B3+
n = 852
R2 = 0.01
(a)
[4]B3+
n = 672
R2 = 0.18
(b)
[4]Si4+
n = 2282
R2 = 0.01
(c)
[6]Si4+
n = 24
R2 = 0.07
(d)
[4]Ge4+
n = 279
R2 = 0.01
(e)
[5]Ge4+
n = 12
R2 = 0.19
(f)
[6]Ge4+
n = 44
R2 = 0.08
(g)
[3]As3+
n = 9
R2 = 0.23
(h)
[4]As3+
n = 4
R2 = 0.98
(i)
[5]As3+
n = 8
R2 = 0.71
(j)
[6]As3+
n = 6
R2 = 0.99
(k)
[8]As3+
n = 1
(l)
[4]As5+
n = 508
R2 = 0.04
(m)
[6]As5+
n = 13
R2 = 0.00
(n)
[3]Sb3+
n = 6
R2 = 0.27
(o)

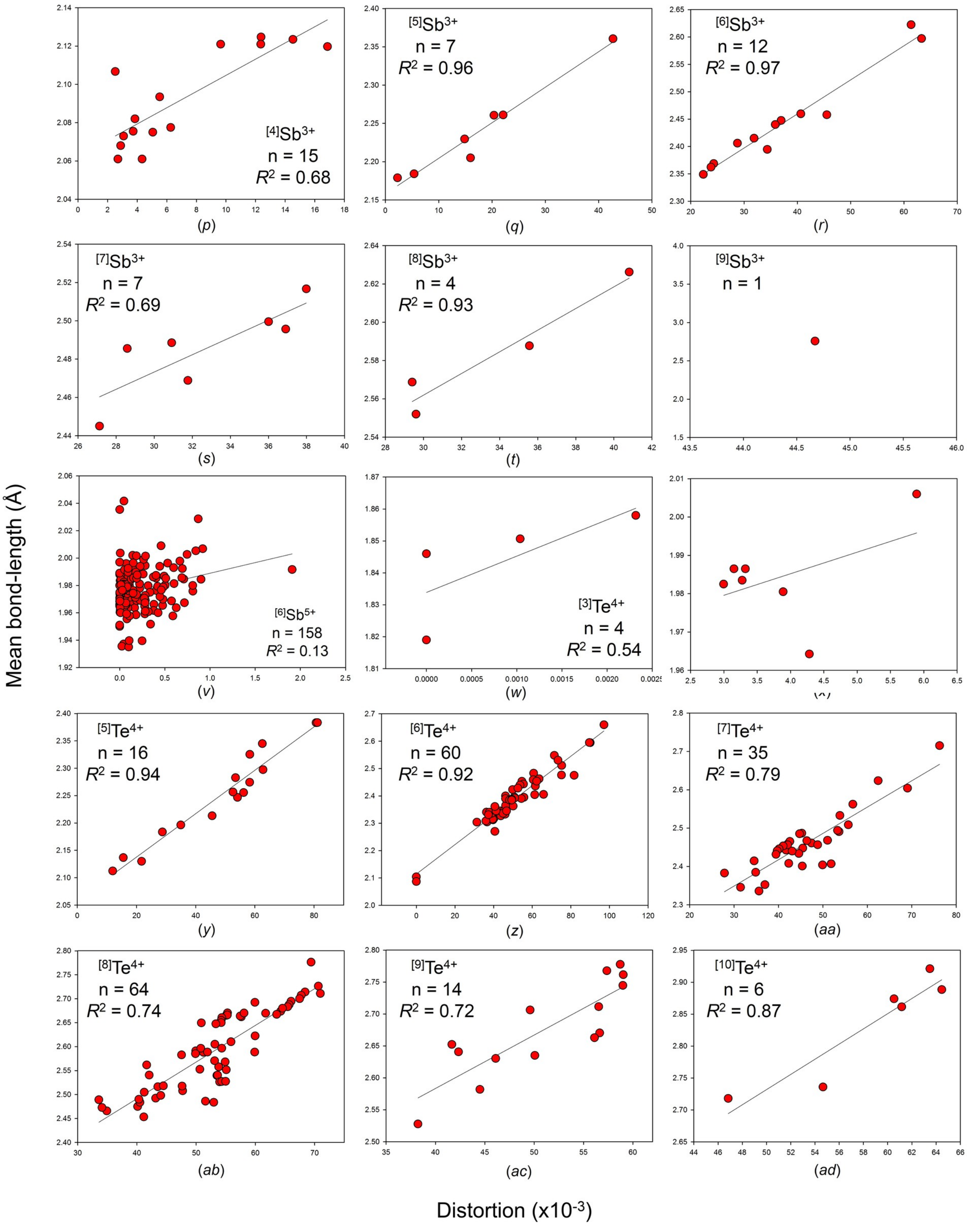

[4]Sb3+
n = 15
R2 = 0.68
(p)
[5]Sb3+
n = 7
R2 = 0.96
(q)
[6]Sb3+
n = 12
R2 = 0.97
(r)
[7]Sb3+
n = 7
R2 = 0.69
(s)
[8]Sb3+
n = 4
R2 = 0.93
(t)
[9]Sb3+
n = 1
[6]Sb5+
n = 158
R2 = 0.13
(v)
[3]Te4+
n = 4
R2 = 0.54
(w)
[5]Te4+
n = 16
R2 = 0.94
(y)
[6]Te4+
n = 60
R2 = 0.92
(z)
[7]Te4+
n = 35
R2 = 0.79
(aa)
[8]Te4+
n = 64
R2 = 0.74
(ab)
[9]Te4+
n = 14
R2 = 0.72
(ac)
[10]Te4+
n = 6
R2 = 0.87
(ad)
Mean bond-length (Å)
Distortion (x10-3)

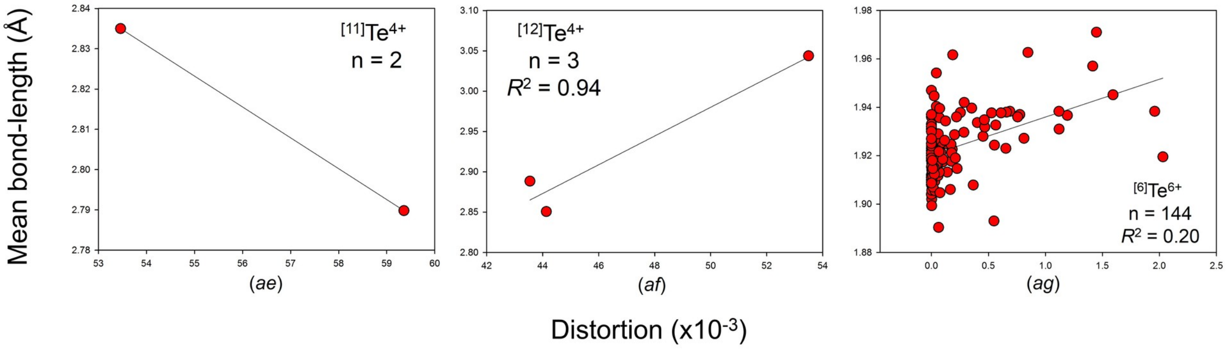
Mean bond-length (Å)
[11]Te4+
n = 2
(ae)
[12]Te4+
n = 3
R2 = 0.94
(af)
[6]Te6+
n = 144
R2 = 0.20
(ag)
Distortion (x10-3)

Figure S8 The effect of bond-length distortion on mean bond-length for all configurations of the post-transition-metal ions bonded to O^{2-}: (*a*) $^{[4]}Al^{3+}$, (*b*) $^{[5]}Al^{3+}$, (*c*) $^{[6]}Al^{3+}$, (*d*) $^{[4]}Ga^{3+}$, (*e*) $^{[5]}Ga^{3+}$, (*f*) $^{[6]}Ga^{3+}$, (*g*) $^{[6]}In^{3+}$, (*h*) $^{[7]}In^{3+}$, (*i*) $^{[8]}In^{3+}$, (*j*) $^{[3]}Sn^{2+}$, (*k*) $^{[4]}Sn^{2+}$, (*l*) $^{[5]}Sn^{2+}$, (*m*) $^{[6]}Sn^{2+}$, (*n*) $^{[7]}Sn^{2+}$, (*o*) $^{[8]}Sn^{2+}$, (*p*) $^{[9]}Sn^{2+}$, (*q*) $^{[4]}Sn^{4+}$, (*r*) $^{[6]}Sn^{4+}$, (*s*) $^{[7]}Sn^{4+}$, (*t*) $^{[3]}Tl^{+}$, (*u*) $^{[4]}Tl^{+}$, (*v*) $^{[5]}Tl^{+}$, (*w*) $^{[6]}Tl^{+}$, (*x*) $^{[7]}Tl^{+}$, (*y*) $^{[8]}Tl^{+}$, (*z*) $^{[9]}Tl^{+}$, (*aa*) $^{[10]}Tl^{+}$, (*ab*) $^{[11]}Tl^{+}$, (*ac*) $^{[12]}Tl^{+}$, (*ad*) $^{[6]}Tl^{3+}$, (*ae*) $^{[7]}Tl^{3+}$, (*af*) $^{[8]}Tl^{3+}$, (*ag*) $^{[3]}Pb^{2+}$, (*ah*) $^{[4]}Pb^{2+}$, (*ai*) $^{[5]}Pb^{2+}$, (*aj*) $^{[6]}Pb^{2+}$, (*ak*) $^{[7]}Pb^{2+}$, (*al*) $^{[8]}Pb^{2+}$, (*am*) $^{[9]}Pb^{2+}$, (*an*) $^{[10]}Pb^{2+}$, (*ao*) $^{[11]}Pb^{2+}$, (*ap*) $^{[12]}Pb^{2+}$, (*aq*) $^{[4]}Pb^{4+}$, (*ar*) $^{[5]}Pb^{4+}$, (*as*) $^{[6]}Pb^{4+}$, (*at*) $^{[3]}Bi^{3+}$, (*au*) $^{[4]}Bi^{3+}$, (*av*) $^{[5]}Bi^{3+}$, (*aw*) $^{[6]}Bi^{3+}$, (*ax*) $^{[7]}Bi^{3+}$, (*ay*) $^{[8]}Bi^{3+}$, (*az*) $^{[9]}Bi^{3+}$, (*ba*) $^{[10]}Bi^{3+}$, (*bb*) $^{[12]}Bi^{3+}$, (*bc*) $^{[4]}Bi^{5+}$, (*bd*) $^{[6]}Bi^{5+}$.

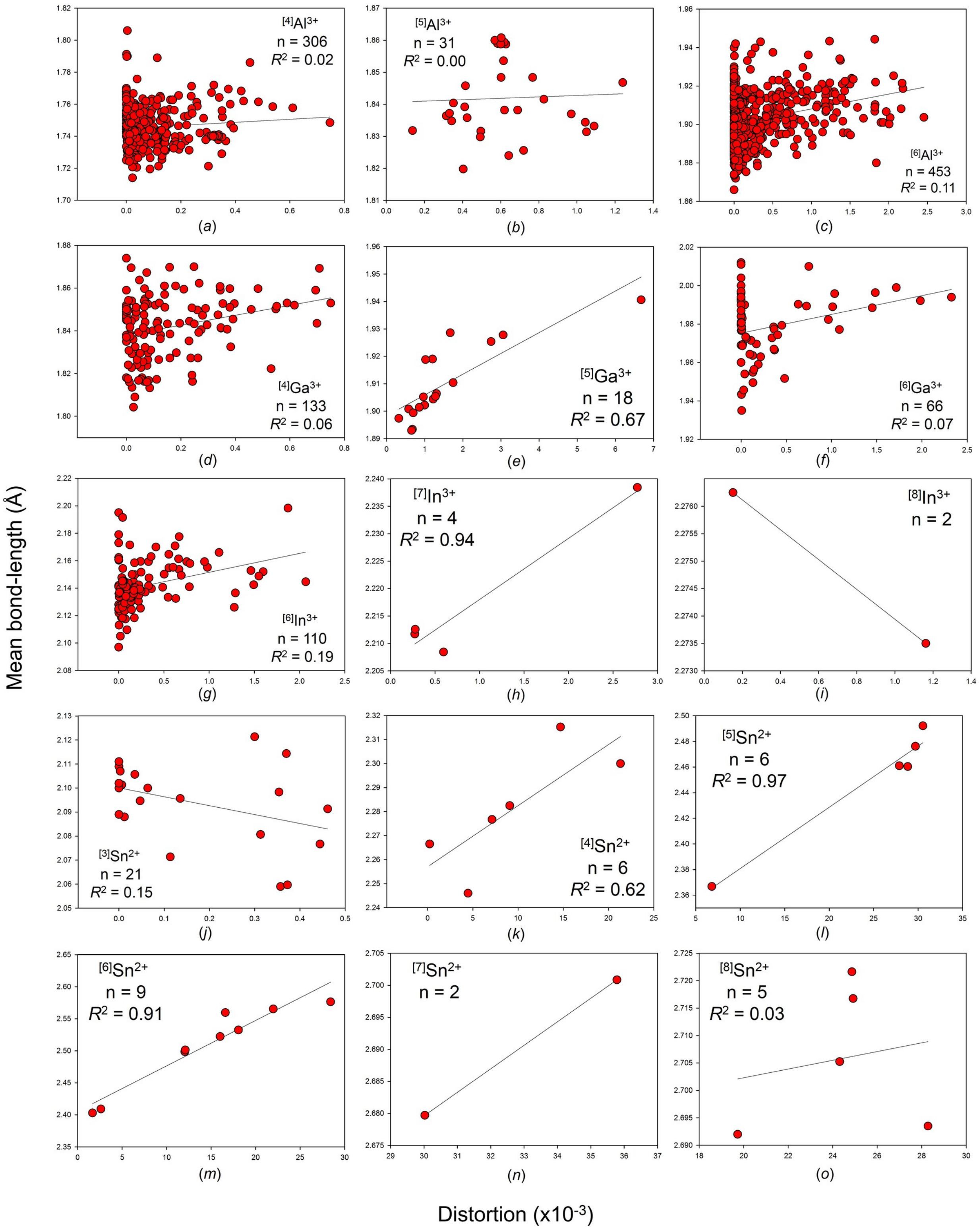

Mean bond-length (Å)
Distortion (x10-3)
[4]Al3+
n = 306
R2 = 0.02
(a)
[5]Al3+
n = 31
R2 = 0.00
(b)
[6]Al3+
n = 453
R2 = 0.11
(c)
[4]Ga3+
n = 133
R2 = 0.06
(d)
[5]Ga3+
n = 18
R2 = 0.67
(e)
[6]Ga3+
n = 66
R2 = 0.07
(f)
[6]In3+
n = 110
R2 = 0.19
(g)
[7]In3+
n = 4
R2 = 0.94
(h)
[8]In3+
n = 2
(i)
[3]Sn2+
n = 21
R2 = 0.15
(j)
[4]Sn2+
n = 6
R2 = 0.62
(k)
[5]Sn2+
n = 6
R2 = 0.97
(l)
[6]Sn2+
n = 9
R2 = 0.91
(m)
[7]Sn2+
n = 2
(n)
[8]Sn2+
n = 5
R2 = 0.03
(o)

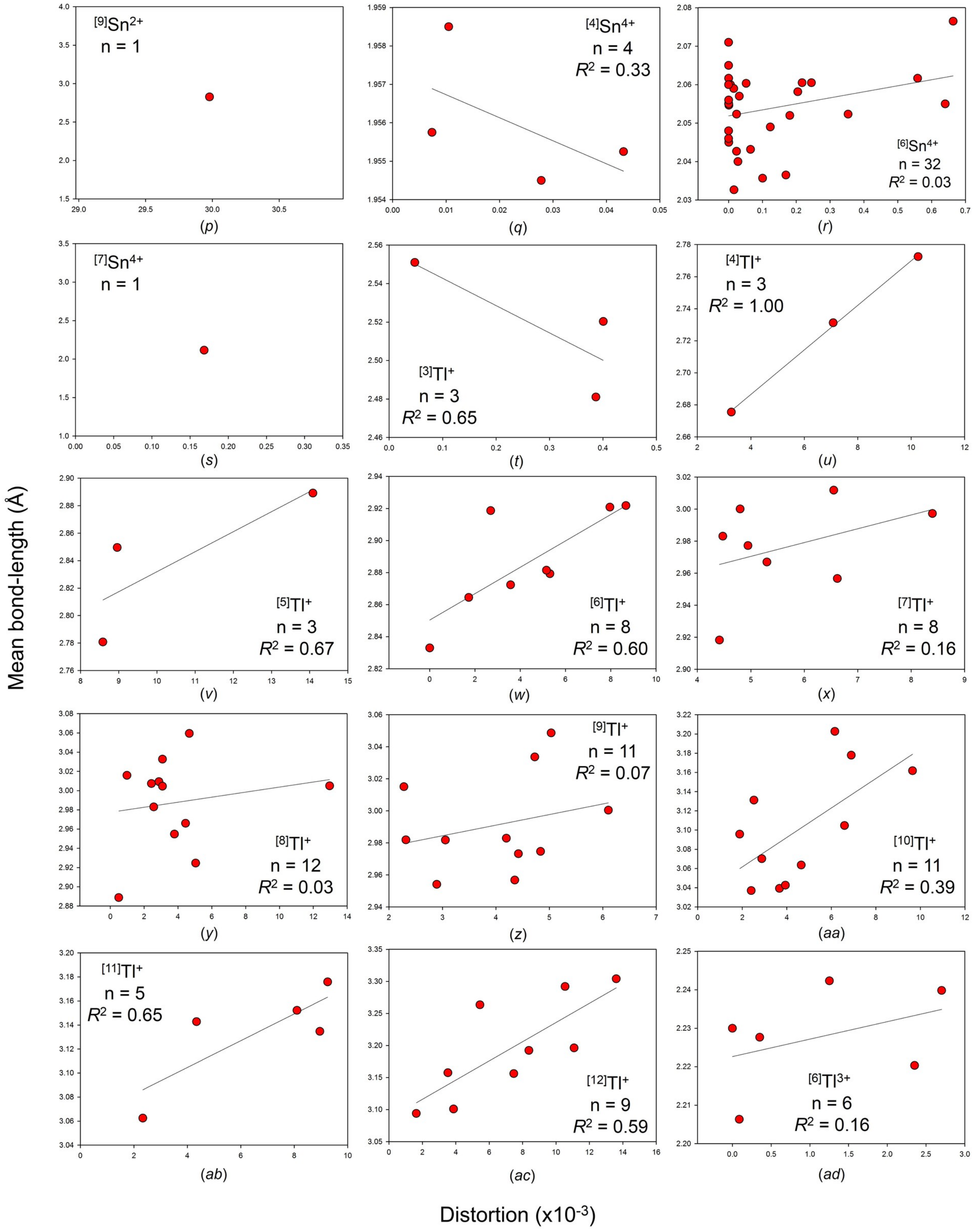

[9]Sn2+
n = 1
(p)
[4]Sn4+
n = 4
R2 = 0.33
(q)
[6]Sn4+
n = 32
R2 = 0.03
(r)
[7]Sn4+
n = 1
(s)
[3]Tl+
n = 3
R2 = 0.65
(t)
[4]Tl+
n = 3
R2 = 1.00
(u)
[5]Tl+
n = 3
R2 = 0.67
(v)
[6]Tl+
n = 8
R2 = 0.60
(w)
[7]Tl+
n = 8
R2 = 0.16
(x)
[8]Tl+
n = 12
R2 = 0.03
(y)
[9]Tl+
n = 11
R2 = 0.07
(z)
[10]Tl+
n = 11
R2 = 0.39
(aa)
[11]Tl+
n = 5
R2 = 0.65
(ab)
[12]Tl+
n = 9
R2 = 0.59
(ac)
[6]Tl3+
n = 6
R2 = 0.16
(ad)
Mean bond-length (Å)
Distortion (x10-3)

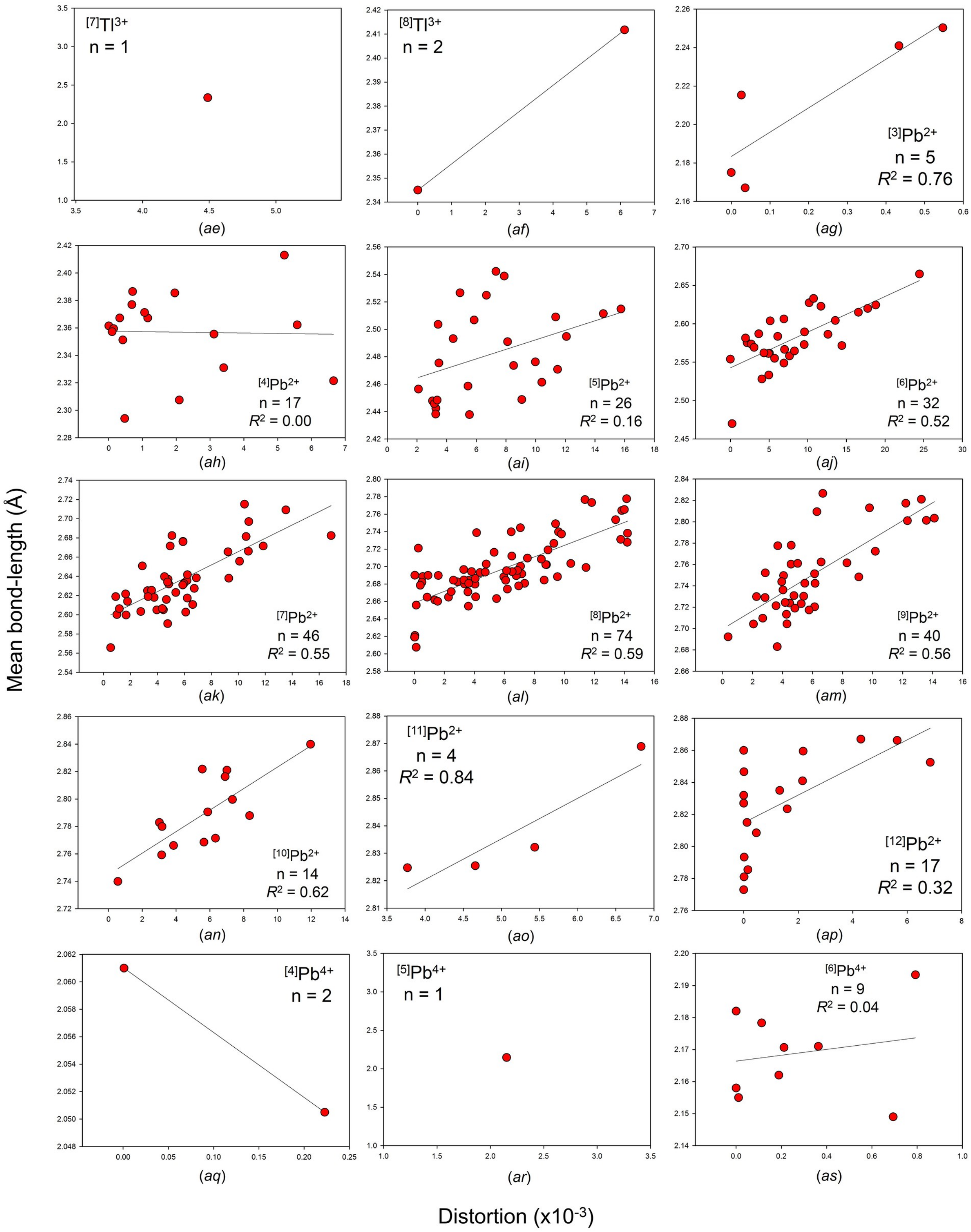
[7]Tl3+
n = 1
(ae)
[8]Tl3+
n = 2
(af)
[3]Pb2+
n = 5
R2 = 0.76
(ag)
[4]Pb2+
n = 17
R2 = 0.00
(ah)
[5]Pb2+
n = 26
R2 = 0.16
(ai)
[6]Pb2+
n = 32
R2 = 0.52
(aj)
[7]Pb2+
n = 46
R2 = 0.55
(ak)
[8]Pb2+
n = 74
R2 = 0.59
(al)
[9]Pb2+
n = 40
R2 = 0.56
(am)
[10]Pb2+
n = 14
R2 = 0.62
(an)
[11]Pb2+
n = 4
R2 = 0.84
(ao)
[12]Pb2+
n = 17
R2 = 0.32
(ap)
[4]Pb4+
n = 2
(aq)
[5]Pb4+
n = 1
(ar)
[6]Pb4+
n = 9
R2 = 0.04
(as)
Mean bond-length (Å)
Distortion (x10-3)

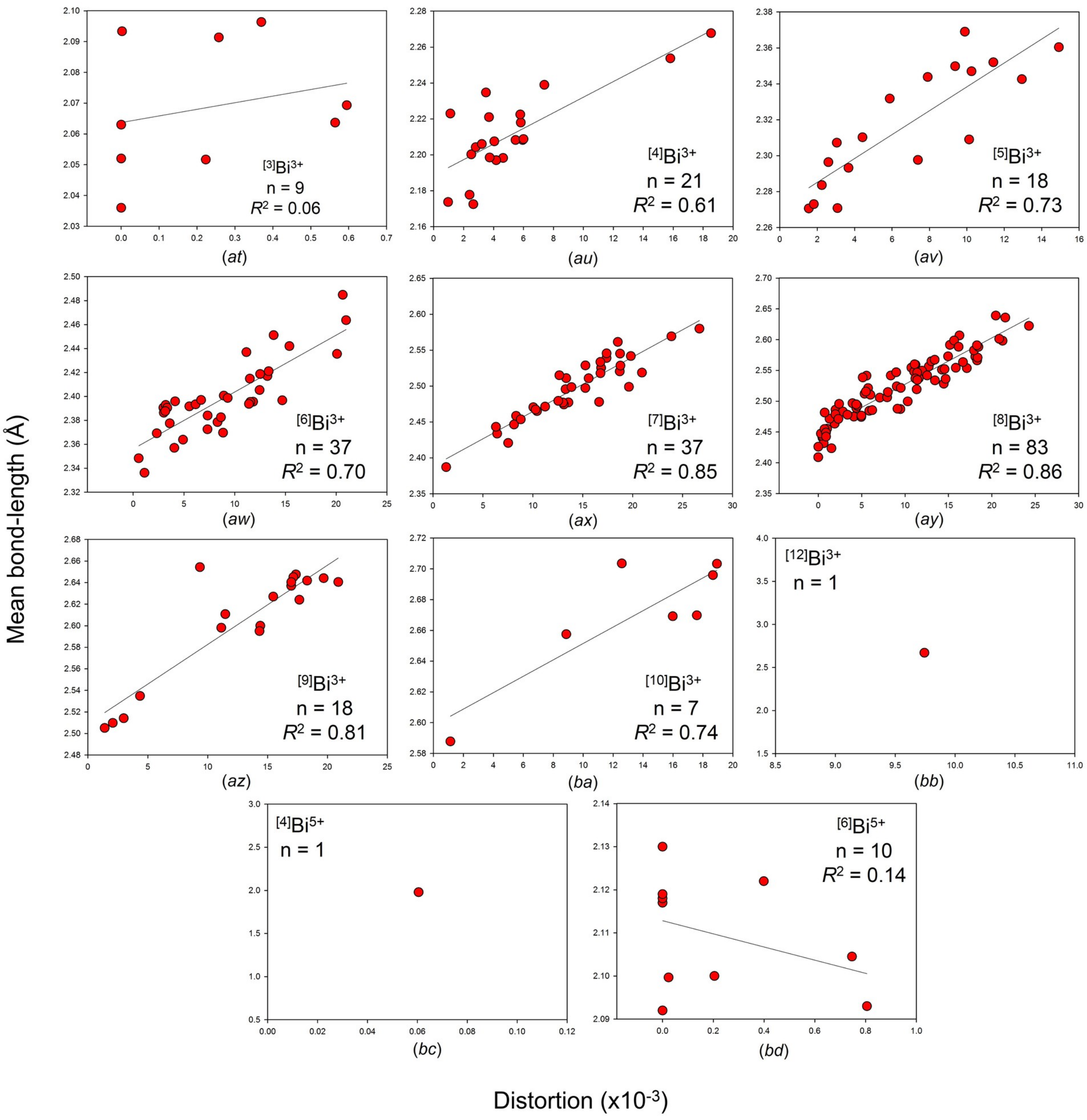

Mean bond-length (Å)
Distortion (x10-3)
[3]Bi3+ n = 9 R2 = 0.06
(at)
[4]Bi3+ n = 21 R2 = 0.61
(au)
[5]Bi3+ n = 18 R2 = 0.73
(av)
[6]Bi3+ n = 37 R2 = 0.70
(aw)
[7]Bi3+ n = 37 R2 = 0.85
(ax)
[8]Bi3+ n = 83 R2 = 0.86
(ay)
[9]Bi3+ n = 18 R2 = 0.81
(az)
[10]Bi3+ n = 7 R2 = 0.74
(ba)
[12]Bi3+ n = 1
(bb)
[4]Bi5+ n = 1
(bc)
[6]Bi5+ n = 10 R2 = 0.14
(bd)
